# Profiling associations of interactive ligand–receptors (HLA class I and KIR gene products) with the progression to type 1 diabetes among seroconverted participants

**DOI:** 10.1007/s00125-025-06520-5

**Published:** 2025-08-21

**Authors:** Lue Ping Zhao, George K. Papadopoulos, Benjamin J. McFarland, Jay S. Skyler, Hemang M. Parikh, William W. Kwok, Terry P. Lybrand, George P. Bondinas, Antonis K. Moustakas, Ruihan Wang, Chul-Woo Pyo, Wyatt C. Nelson, Daniel E. Geraghty, Åke Lernmark

**Affiliations:** 1 Public Health Sciences Division, Fred Hutchinson Cancer Research Centre, Seattle, WA USA; 2 School of Public Health, University of Washington, Seattle, WA USA; 3 Laboratory of Biophysics, Biochemistry, Biomaterials and Bioprocessing, Faculty of Agricultural Technology, Technological Educational Institute (TEI) of Epirus, Arta, Greece; 4 Department of Biochemistry, Seattle Pacific University, Seattle, USA; 5 Diabetes Research Institute and Division of Endocrinology, Diabetes & Metabolism, University of Miami Miler School of Medicine, Miami, Florida USA; 6 Health Informatics Institute, Morsani College of Medicine, University of South Florida, Tampa, FL USA; 7 Benaroya Research Institute, Seattle, WA USA; 8 Department of Chemistry, Vanderbilt University, Nashville, TN USA; 9 Department of Food Science and Technology, Faculty of Environmental Sciences, Ionian University, Argostoli, Cephalonia Greece; 10 Clinical Research Division, Fred Hutchinson Cancer Research Centre, Seattle, WA USA; 11 Department of Clinical Sciences, Lund University CRC, Skåne University Hospital, Malmö, Sweden

**Keywords:** HLA, Immunogenetics, Islet autoimmunity, Killer-cell immunoglobulin-like receptors, KIR, Natural killer (NK) cells, Progression, Seroconversion, Type 1 diabetes

## Abstract

**Aims/hypothesis:**

The aim of this work was to explore associations between type 1 diabetes progression from stages 1 or 2 to stage 3 and interacting ligand–receptor complexes of HLA class I (HLA-I) and KIR gene products.

**Methods:**

Applying next-generation sequencing technology to genotype HLA-I genes (*HLA-A*, *-B*, *-C*) and KIR genes (*KIR2DL1*, *KIR2DL2*, *KIR2DL3*, *KIR2DL4*, *KIR2DL5*, *KIR2DS1*, *KIR2DS2*, *KIR2DS3*, *KIR2DS4*, *KIR2DS5*, *KIR3DL1*, *KIR3DL3*, *KIR3DS1*, *KIR2DP1*, *KIR3DP1*) from 1215 participants in the Diabetes Prevention Trial-Type 1 (DPT-1) and the Diabetes Prevention Trial (TN07), we systematically explored associations of HLA-I–KIR ligand–receptor interactions (LRIs) with disease progression via a Cox regression model. We investigated the structural properties of identified LRI complexes.

**Results:**

*KIR* and *HLA-I* genes had no or sporadic associations with disease progression. Out of all possible LRIs, nine HLA-A Ligands and 14 HLA-B ligands with corresponding receptors had modest associations with progression (*p*<0.05). As an example, carriers of A*03:01-KIR2DS4 had slower progression (HR 0.36, *p*=3.06 × 10^−2^), as did B*07:02-KIR2DL3 carriers (HR 0.26, *p*=7.76 × 10^−3^). Structural investigations of KIR–HLA-I complexes via homology modelling based on already-solved respective complex structures suggested that the respective electrostatic and van der Waals interactions encoded in the protein sequences result in strong biophysical LRIs, which could alter the progression of type 1 diabetes.

**Conclusions/interpretation:**

These results reveal that LRIs of KIR–HLA-I gene products, rather than individual genes, contribute to type 1 diabetes progression, and such interactions are likely to be stabilised by electrostatic and van der Waals forces. As the KIR–HLA-I interactions involve part of the C-terminus of the antigen-binding groove of HLA-I, but may be affected by the respective bound peptide, this suggests a new mechanism for type 1 diabetes pathogenesis.

**Data availability:**

Clinical data on participants in DPT-1 and TN07 can be obtained from the NIDDK-Central Repository (https://repository.niddk.nih.gov/home) following the formal approval process.

**Graphical Abstract:**

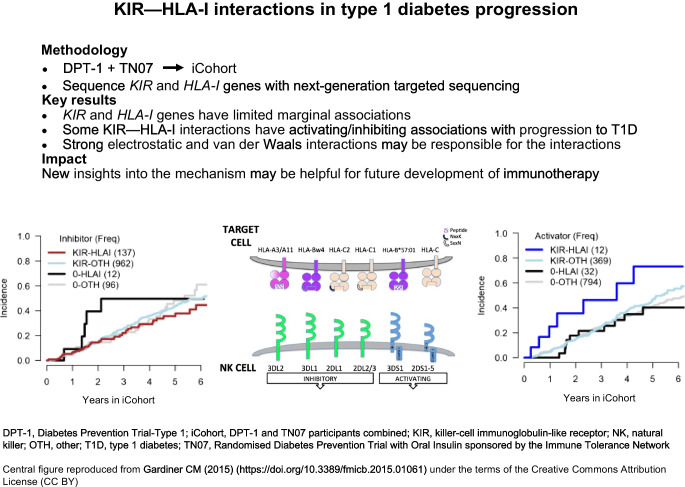

**Supplementary Information:**

The online version contains peer-reviewed but unedited supplementary material available at 10.1007/s00125-025-06520-5.


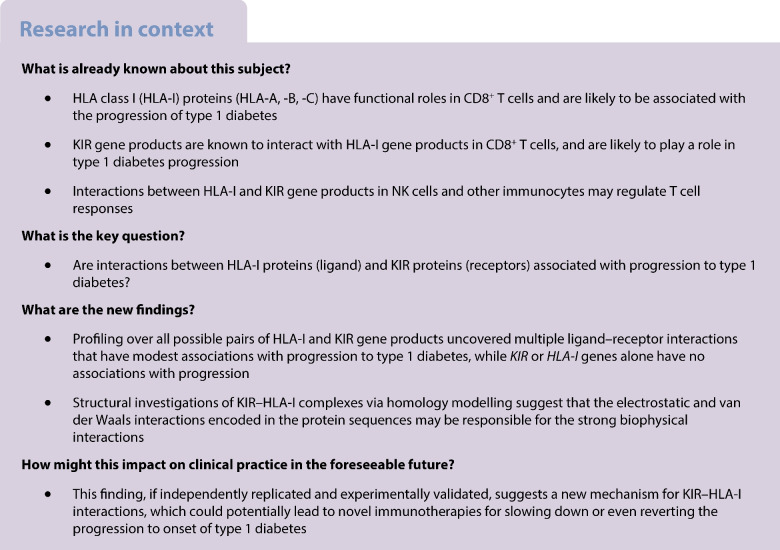


## Introduction

Type 1 diabetes is an autoimmune disease, resulting from destruction of functional beta cells by the host immune system [1, 2]. The course of the disease gradually evolves from homeostasis with normal cellular functions to seroconversion (stage 1), hyperglycaemia (stage 2) and finally onset of symptomatic type 1 diabetes (stage 3) [3]. While environmental factors can trigger the disease, host genetic factors play essential roles throughout type 1 diabetes development [3–9], especially *HLA class II* (HLA-II) genes, which account for over 50% of the genetic risk [10–15]. Recently, we have shown that HLA-II genes also contribute to disease progression [9, 16]. However, much less is known about *HLA class I* (HLA-I) genes and disease progression. Given their functions in CD8^+^ T cells, it is natural to hypothesise that *HLA-I* genes may influence type 1 diabetes progression.

Like CD8^+^ T cells, natural killer (NK) cells are responsible for eliminating ‘signalled cells’ (virally infected or malignantly transformed) during disease progression and are influenced by polymorphic killer-cell immunoglobulin-like receptors (KIRs) [17]. NK cells have rarely been found in infiltrated islets from autopsies of individuals who died soon after disease onset, yet this may not be a true reflection of the role of NK cells in progression from stage 1 disease to the high-grade insulitis observed in autopsies of islet tissue [18]. In a pioneering study of samples from inguinal lymph nodes of individuals newly diagnosed with type 1 diabetes, compared with control individuals, several disparities were noted between immunocyte types in peripheral blood and the said lymph nodes [18]. In particular, only the CD56^bright^ NK cell subset was noted in the lymph nodes. Several other cell subpopulation differences were recorded in peripheral blood and lymph node cells, in the percentage composition of such subgroups and in the presence of key markers of particular cell subpopulations that differentiate the function of such cells [18]. KIRs have been found to interact with micromolar affinity with HLA-I molecules as ligands, leveraging peptide-binding specificity to improve the ‘killing specificity’ [19]. In a recent longitudinal study of newborns, there was evidence to support the involvement of NK cells in type 1 diabetes progression [20]. In autopsies of islet tissue from individuals with type 1 diabetes, HLA-I molecules are hyper-expressed in islet beta cells [18, 21]. If NK cells are recruited to such hyper-expressing islets, then the chances of interaction are considerable [22]. It is therefore of interest to investigate whether KIR–HLA-I interactions, such as ligand–receptor interactions (LRIs), contribute to the progression of type 1 diabetes.

In this study, we accessed DNA samples from participants in two completed clinical trials (Diabetes Prevention Trial-Type 1 [DPT-1] and Randomised Diabetes Prevention Trial with Oral Insulin sponsored by TrialNet [TN07]) and sequenced 16 *KIR* genes in addition to *HLA-A, -B* and *-C* genes. Together with available clinical/demographic data from the National Institute of Diabetes and Digestive and Kidney Diseases (NIDDK) Central Repository (NIDDK-CR), we tested the hypothesis that KIR–HLA-I interactions may contribute towards progression to type 1 diabetes.

## Methods

### Diabetes prevention trials (DPT-1 and TN07)

Participants in two randomised prevention trials of insulin, DPT-1 and TN07, were combined into an integrated cohort (iCohort) to investigate the immunogenetic associations with disease progression [8, 23, 24]. Briefly, 670 participants in DPT-1 were recruited from multiple centres in the USA and were selected based on elevated autoantibody levels and positive family history, including those with high and modest risks of progressing to type 1 diabetes within 5 years [25]. Based on design considerations, TN07 launched a multicentre international clinical trial and recruited 546 individuals based on elevated autoantibody levels and positive family history [26]. One participant was excluded because of failure to genotype the DNA sample. The distributions of key demographic/study variables and their associations with type 1 diabetes onset are shown in electronic supplementary material (ESM) ESM Table 1. The iCohort comprises predominantly young male and female participants of European ancestry from Europe and the USA, in which sex was self-determined using a questionnaire.

### Genotyping HLA and KIR using next-generation targeted sequencing technology

For next-generation targeted sequencing (NGTS), HLA and KIR typing was carried out using the ScisGo-HLA-v6 and ScisGo-KIR-v3 typing kits (Scisco Genetics, Seattle, WA, USA) following the kit protocols. Briefly, the method employs an amplicon-based two-stage PCR, followed by sample pooling and sequencing using a MiSeq v2 PE500 (Illumina, San Diego, CA, USA). Each distinct lot and shipment of kits was tested using a control panel of DNAs and the results were validated in accordance with ASHI standards and practice (https://www.ashi-hla.org/page/standards, accessed 9 Sep 2024). Each 96-sample plate typed was populated with positive and negative controls placed strategically to identify the orientation and origin of the plate. Duplicate iCohort samples were arranged on different plates as additional confirmation of plate structures. All control and duplicate iCohort sample genotypes and their positions on the individual plates were validated upon typing.

The HLA protocol yielded three-field coverage of all HLA- genes *(HLA-A*, *-B* and *-C*) plus detection of all known four-field N, Q, L and S alleles. The phase within each gene was determined by bridging amplicons and database lookup for HLA-I [24]. The ScisGo-KIR-v3 kit was used to sequence eight exons and pseudo exons including flanking intron sequences of 15 KIR genes (*KIR2DL1*, *KIR2DL2*, *KIR2DL3*, *KIR2DL4*, *KIR2DL5*, *KIR2DS1*, *KIR2DS2*, *KIR2DS3*, *KIR2DS4*, *KIR2DS5*, *KIR3DL1*, *KIR3DL3*, *KIR3DS1*, *KIR2DP1* and *KIR3DP1*), allowing for the determination of KIR gene copy number and haplotype structure. Note that *KIR3DL3* is monomorphic at two copies and thus was excluded from the study.

### Modelling KIR–HLA-I interactions and general statistical evaluation

Cox regression has been used previously to model immunogenetic associations of HLA genes with progression to type 1 diabetes: one allele vs all other alleles while adjusting for possible confounders [9, 27]. The same Cox model was expanded to quantify KIR–HLA-I interactions through explicitly modelling interactions of KIR and HLA-I proteins, as detailed in ESM Methods. Briefly, considering a specific *HLA-I* gene, we transformed an *HLA-I* genotype observed from an individual to an allelic count (0, 1, 2) of a specific *HLA-I* allele, and the same was carried out for *KIR* genes. The cross-product of HLA-I allelic count and KIR indicator quantified their interactions, after adjusting for main HLA-I and KIR effects (model 1 [m1]). Where KIR and HLA-I were highly correlated and isolating main HLA-I or KIR effects was not feasible, we focused on the interaction, ignoring main effects (model 2 [m2]). All association analyses adjusted for possible confounders (race, sex, age, risk level, use of insulin and population sources). We also adjusted for *HLA-II* genes (*DRB1*, *DQA1*, *DQB1*) to account for possible confounding due to their high linkage disequilibrium (LD) with *HLA-I* genes. High LD also permitted us to infer their haplotypes and computing haplotypic frequencies (see ESM Table 2). Evidently, *DR3-DQ2* genes are linked to multiple *A*, *B* and *C* alleles, and so are *DR4-DQ8* genes.

Besides interaction analysis, all analyses in this project used statistical software R (version 4.4.3) and related packages [28]. For assessing genetic associations with the time-to-onset of type 1 diabetes, we used the ‘coxph’ function and tested the proportionality by ‘cox.zph’ in R. Statistical analysis generally centred on associations of every individual allele vs all others, to avoid choosing a reference allele, as the HLA system is highly polymorphic and variable across populations. The current investigation, using completed clinical trials, should be viewed as exploratory and hypothesis-generating, and used the significance threshold of *p*=0.05.

Pooling DPT-1 and TN07 participants into iCohort increased the sample size and hence improved the power to detect novel KIR–HLA-I interactions, at the expense of losing an independent replication opportunity. To assess the robustness of our results, we sampled 1215 individuals from iCohort with replacement, forming bootstrap samples and performing discovery analysis, and repeated this bootstrap discovery analysis 100,000 times to estimate frequencies (*P*_*1*_) at which selected KIR–HLA-I interactions were discovered. To quantify frequencies under the null hypothesis, we used a permutation strategy to generate, for example, 100,000 replicated studies and to estimate the frequencies (*P*_*0*_) that quantify rates of false discoveries. *P*_*1*_*/P*_*0*_ ratios >1 signified the robustness of discovered KIR–HLA-I interactions.

### Homology modelling and template selection for KIR–HLA-I complexes

KIR–HLA-I interactions were investigated by homology-based structural analyses, as detailed in ESM Methods. Briefly, SWISS-MODEL (https://swissmodel.expasy.org/, accessed 15 Sep 2024) was used to compare three KIR–HLA-I complex templates for 41 complexes, and the 4N8V.pdb ID template of KIR2DS2 bound to HLA-A*11:01 produced greater predicted interaction strengths and diversities of interactions for >70% of the complexes; also, this template’s predicted pseudo energies were most similar to the properties of six KIR–HLA-I crystal structures. Since 11 of the 15 KIRs had two domains, the two-domain high-scoring 4N8 V template matched the structure of the majority of our dataset and was used as a template for further analysis.

## Results

### Marginal associations of *HLA-A*, -*B* and *-C* with type 1 diabetes progression

*HLA-A, -B* and -*C* are highly polymorphic (for allelic frequencies, see ESM Tables 3–5) and alleles with fewer than ten observations in iCohort were grouped as rare alleles. Allelic associations were assessed through regressing type 1 diabetes onset time on the allelic count allele-by-allele. While unadjusted analyses (ESM Tables 3–5) regressed only on HLA-I alleles in the Cox models, in adjusted analyses there was an additional adjustment for potential risk factors and *HLA-II* genotypes through inclusion of a ‘confounding offset’. From both analyses, we estimated the regression coefficient, HR, SE, *z* score and *p* value for every *HLA-I* allele. An allele with a *p* value threshold of 0.05 and an HR >1 was considered either to accelerate type 1 diabetes progression (*p* value highlighted in blue) or to delay progression (*p* value highlighted in brown). The *HLA-A* locus had 18 common alleles in the iCohort, accounting for 97% of all observed alleles. Among them, HLA-A*24:02 was found to have an allelic association in the adjusted analysis (HR 1.42, *p*=0.005, ESM Table 3), while the same allele in the unadjusted analysis had a non-significant *p* value but in the same direction (HR 1.27, *p*=0.06). The *HLA-B* locus had 31 common alleles, accounting for 94% of observed alleles (ESM Table 4). While most of the alleles had no associations with type 1 diabetes progression, HLA-B*37:01 had a significant protective association in the adjusted analysis but not in the unadjusted analysis (HR 0.29 and 0.36, *p*=0.03 and 0.08, respectively, ESM Table 4). In the *HLA-C* locus with 18 common alleles, none was found to have a risk association in the adjusted analysis (ESM Table 5).

### Marginal associations of KIRs with type 1 diabetes progression

In total, 16 *KIR* genes have been sequenced (*KIR2DL1*, *KIR2DL2*, *KIR2DL3*, *KIR2DL4*, *KIR2DL5*, *KIR2DS1*, *KIR2DS2*, *KIR2DS3*, *KIR2DS4*, *KIR2DS5*, *KIR3DL1*, *KIR3DL2*, *KIR3DS1*, *KIR2DP1*, *KIR3DP1* and *KIR3DL3*), of which *KIR3DL3* is monomorphic and was excluded. KIRs have variable numbers of receptors, from 0 to 4 (ESM Table 6). Regressing type 1 diabetes onset time on the number of receptors, we found that none of the KIRs associated with type 1 diabetes progression according to the *p* value threshold of 0.05 in either the unadjusted or the adjusted analyses (ESM Table 6).

### Associations of KIR–HLA-A interactions with type 1 diabetes progression

*HLA-I* genes encode the α-chain of the HLA-I molecule (ligand), which is associated non-covalently with the invariant β_2_ microglobulin chain (see ESM Figs 1, 2), while KIRs have variable numbers of receptors for HLA-I molecules. A heterozygous *HLA-I* gene locus, with one or more KIR receptors, can have up to four distinct ligand–receptor pairs via KIR–HLA-I interactions (ESM Fig. 2). Here, we focus on the interaction of a specific HLA-A Ligand with KIR receptors as defined above. Through pairing 19 HLA-A proteins (including rare alleles merged into a single group) and 15 KIR receptors, we screened 285 possible KIR–HLA-I interactions after adjusting for the confounding effect (ESM Table 7, ESM Fig. 3a). Based on the *p*=0.05 threshold, we found four unique interactions (Table 1) from models A.1–A.4. Under model A.1, the A*03:01–KIR2DS4 interaction was found to negatively associate with progression to type 1 diabetes (HR 0.36, *p*=0.03) for 88 participants with disease with the interaction and 167 disease-free participants. The KIR2DS4 receptor is referred to as inhibitory. A similar observation was seen in the unadjusted analysis (HR 0.34, *p*=0.02, ESM Table 7). The bootstrap analysis indicated that this interaction was discovered in 58% of replicates (*P*_*1*_=0.58), with a 4% false discovery rate (Table 1). The discovery ratio of *P*_*1*_*/P*_*0*_=14.5 highly favoured the inhibitory receptor.

**Table 1:** Association results for KIR and HLA-A/B interactions with type 1 diabetes progression

seq	KIR–HLA-I	n0	n1	coef	HR	SE	*z* score	*p* value	*P*_*1*_	*P*_*0*_	*P*_*1*_*/P*_*0*_
A.1	A*03:01–KIR2DS4-m1^a^	167	88	−1.01	0.36	0.47	−2.16	3.06 × 10^−2^	0.58	0.04	14.25
A.2	A*24:02–KIR2DL1-m2^b^	108	64	0.38	1.47	0.14	2.80	5.18 × 10^−3^	0.73	0.07	10.11
A.3	A*24:02–KIR2DL4-m2^c^	111	64	0.38	1.46	0.14	2.77	5.56 × 10^−3^	0.79	0.05	16.86
A.4	A*25:01–KIR2DS5-m1	5	7	0.99	2.70	0.50	2.00	4.54 × 10^−2^	0.51	0.04	13.84
B.1	B*07:02–KIR2DL3-m1	96	41	−1.36	0.26	0.51	−2.66	7.76 × 10^−3^	0.69	0.04	15.74
B.2	B*15:18–KIR2DL3-m2	6	4	1.03	2.80	0.50	2.05	4.07 × 10^−2^	0.42	0.04	9.33
B.3	B*37:01–KIR2DL1-m2^d^	18	3	−1.17	0.31	0.58	−2.02	4.36 × 10^−2^	0.49	0.04	11.05
B.4	B*37:01–KIR2DL4-m2^e^	19	3	−1.25	0.29	0.58	−2.16	3.11 × 10^−2^	0.58	0.05	12.74
B.5	B*51:01–KIR2DS3-m1	14	12	0.84	2.32	0.42	2.02	4.33 × 10^−2^	0.55	0.04	12.43
B.6	B*55:01–KIR2DS1-m1	14	3	−1.34	0.26	0.67	−2.01	4.41 × 10^−2^	0.47	0.04	11.69
B.7	B*58:01–KIR2DL2-m1	6	2	−3.76	0.02	1.02	−3.69	2.25 × 10^−4^	0.42	0.05	9.21
B.8	B*58:01–KIR2DL5-m1	5	1	−2.36	0.09	1.17	−2.02	4.33 × 10^−2^	0.27	0.02	13.76
B.9	B*58:01–KIR2DS2-m1	6	2	−3.78	0.02	1.02	−3.72	2.01 × 10^−4^	0.42	0.05	9.31

Frequencies of disease-free (n0) and diseased individuals (n1) who carry a specific allele and are positive for a specific KIR, estimated coefficients, HRs, SEs, *z* scores and *p* values for the adjusted analysis are shown. Separately estimated are 100,000 bootstrap-based proportions (*P*_*1*_) to quantify the robustness of the identified interactions, 100,000 permutation-based proportions (*P*_*0*_) and their ratio (*P*_*1*_*/P*_*0*_). Full association results are listed in ESM Tables 7–9

^a^A*03:01-KIR3DL1

^b^A*24:02-KIR2DP1, A*24:02-KIR3DP1

^c^A*24:02-KIR3DL2, A*24:02-KIR3DP1

^d^B*37:01-KIR2DP17

^e^B*37:01-KIR2DS4, B*37:01-KIR3DL1, B*37:01-KIR3DL2, B*37:01-KIR3DP1

The multiple interactions of A*24:02 with two unique KIRs (KIR2DL1~2DP1~3DP1, 2DL4~3DL2~3DP1, where ‘~’ indicates equivalent KIR interactions) were found to have positive associations with type 1 diabetes progression. These receptors are referred to as activating to the ligand A*24:02.

Finally, the interaction A*25:01–KIR2DS5 had a positive association with progression in the adjusted analysis (HR 2.70, *p*=0.05). This interaction was identified favourably 14 times more frequently in the observed data than in the permutation data.

**Fig. 1:**
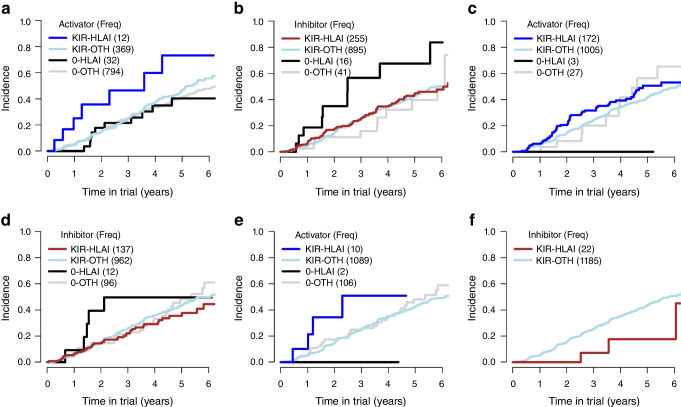
Incidence curves of type 1 diabetes among seroconverted individuals in the DPT-1 and TN07 clinical trials who carried at least one KIR and a specific HLA-I allele (KIR-HLAI), at least one KIR but other HLAI alleles (KIR-OTH), a specific HLA-I allele only (0-HLAI) and other HLA-I alleles only (0-OTH) for six selected LRIs: A*25:01-KIR2DS5 (**a**); A*03:01−KIR2DS4 (**b**); A*24:02−KIR2DL1 (**c**); B*07:02–KIR2DL3 (**d**); B*15:18–KIR2DL3 (**e**); and B*37:01-KIR2DL4 (**f**)

For simplicity of interpreting interaction results, we grouped participants by carrier status of none or at least one KIR (0 vs KIR) and by a specific HLA-I allelic protein vs other HLA-I molecules (HLA-I vs other) and computed their incidence curves (six selected KIR–HLA-I interactions are shown in Fig. 1). The LRI of activating KIR2DS5 with A*25:01 is shown in Fig. 1a (*p*=0.045); 12 carriers appeared to have accelerated progression vs those with KIR but other HLA-I alleles, HLA-I without KIR, or other HLA-I alleles without KIR. Examining the LRI of A*03:01 with inhibitory KIR2DS4 (*p*=0.031, Fig. 1b), HLA-I alone associated with accelerated progression but the interaction with the receptor renders the incidence curve comparable with those of participants without KIR or with other HLA-I alleles. Considering the activating KIR2DL1 with ligand A*24:02 (Fig. 1c), carriers of the HLA-I allele without KIR showed no progression to type 1 diabetes but those who carried the interaction experienced an incidence comparable with that in the two other groups.

### Associations of KIR–HLA-B interactions with type 1 diabetes progression

Pairing 31 *HLA-B* alleles (ESM Table 4) and 15 KIRs (ESM Table 6), we screened for 480 KIR–HLA-B interactions and found nine unique interactions exceeding the *p* value threshold (Table 1 and ESM Table 8, ESM Fig. 3b). Among them, receptor KIR2DL3 interacting with ligands B*07:02 and B*15:18, played both inhibitory and activating roles (HR 0.26 and 2.80, *p*=0.007 and 0.04, respectively). Ligand B*37:01, interacting with receptors KIR2DL1 and KIR2DL4, has a negative association with progression (HR=0.31 and 0.29, *p*=0.04 and 0.03, respectively). Similarly, ligand B*58:01, interacting with KIR2DL2, KIR2DL5 and KIR2DS2, had negative associations with progression (HR 0.02, 0.09 and 0.02, *p*=2.25×10^−4^, *p*=0.04 and *p*=2.01×10^−4^, respectively). The LRI B*51:01–KIR2DS3 was positively associated with progression to type 1 diabetes (HR=2.32, *p*=0.04), while the B*55:01–KIR2DS1 interaction had a negative association (HR 0.26, *p*=0.04).

To gain an insight into these KIR–HLA-I interactions, we selected three LRIs (B*07:02–KIR2DL3, B*15:18–KIR2DL3, B*37:01–KIR2DL4) and computed individual incidence curves in four groups representing the different ligand–receptor combinations (Fig. 1d–f). As noted above, 12 individuals with ligand B*07:02, without the KIR receptor, appeared to have accelerated progression risk (Fig. 1d). For the 137 individuals who carried both HLA-B ligand and KIR2DL3 receptor, the incidence curve was now comparable with those in the two other groups (Fig. 1d). On the other hand, in the ten carriers of the B*15:18–KIR2DL3 interaction, there was accelerated progression (Fig. 1e), while the two carriers of HLA-I alone did not show progression. In the third example, the 22 carriers of the KIR–HLA-I interaction had a much lower incidence curve than the other group (Fig. 1f).

There were no associated interactions of HLA-C and KIR by the pre-established threshold, other than three possible interactions with rare *HLA-C* alleles in the adjusted analysis (ESM Table 9).

### Landscape of progression-associated KIR–HLA-B interactions

**Fig. 2:**
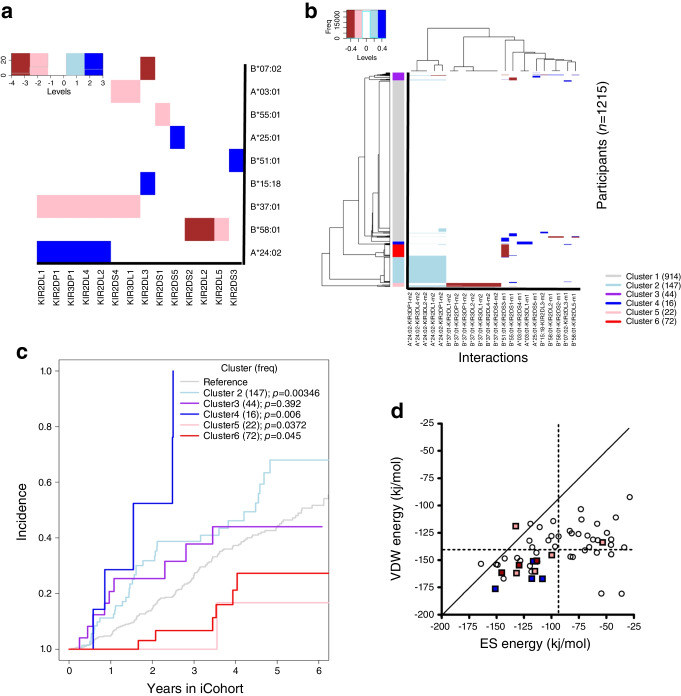
(**a**) Heatmap of *z* scores estimated for all selected LRIs, ranging from −4 to 3. Through hierarchically organising rows and columns, the unsupervised learning grouped *HLA-I* genes and *KIR* genes with similar associations with disease progression closer together. (**b**) Heatmap of predicted values for every individual with 22 KIR–HLA-I interactions, with values ranging from −0.4 to 0.4. Through hierarchically organising columns and rows, the unsupervised learning grouped the LRIs and the individuals with similar associations with disease progression closer together. Participants were organised into six clusters, indicated by the sidebar (sample sizes are shown in the key). (**c**) Incidence curves for the six hierarchically organised clusters of individuals were computed and each cluster was compared with cluster 1 (reference, with no or limited LRIs) with *p* values shown. (**d**) Predicted strengths of van der Waals (VDW) vs electrostatic (ES) binding interactions among KIR–HLA-I complexes modelled on the 4N8V.pdb template. Predicted energies are given in kJ/mol, as calculated by the PPCheck server. Electrostatic and van der Waals interactions together accounted for 80–95% of the total binding energy. The 12 KIR2D–HLA-A/B complexes formed by the nine KIRs that matched the structural template used (according to the criteria described in Results) are represented as squares coloured brown, pink or blue, as for the heatmap shown in (**a**). Other modelled complexes are shown as white circles. Mean values for VDW and ES energies are shown as dashed Lines, so that more stable complexes with more negative predicted energies are located in the lower left quadrant. Ten of the 12 KIR2D–HLA complexes, and all of those shown in brown or blue in the heatmap, are predicted to be more stable than average in both dimensions

Evidently, KIR–HLA-I interactions can involve one ligand with multiple receptors, or one receptor for multiple ligands. To establish their pairing concordances, we created a matrix of *z* scores quantifying for selected LRIs with ligands as rows and receptors as columns for all identified interaction pairs, in which *z* scores ranged from −4 to 3 and were set to zero for insignificant scores falling in the interval (−1.96, 1.96). Hierarchical clustering [29] was applied to organise ligands and receptors by their similarities and to represent them in a heatmap (Fig. 2a). From the Ligand perspective, nine Ligands can interact with one or more receptors, and associations of their LRIs with progression are either consistently positive or consistently negative. On the other hand, 14 receptors could interact with one or more ligands, and they could be either activating or inhibitory, depending on which ligands are interacting (Fig. 2a). For example, the receptor KIR2DL3 is an activator (HR 2.80) when interacting with B*15:18, but is switched to an inhibitor if interacting with B*07:02 (HR 0.26).

In the iCohort, some of the 1215 participants carried one or more of the identified 22 KIR–HLA-I interactions, while most participants probably did not. To gain a holistic view of these 22 LRIs in the iCohort, we used interaction models (A.1–A.4, B.1–B.9) and computed ‘linear predictive values’ based on the Cox regression model, creating a matrix of predictive values (1215 by 22), with values ranging from −0.4 to 0.4. Applying the unsupervised learning technique, we hierarchically organised and displayed these predictive values in a heatmap (Fig. 2b). Noticeably, 147 participants consistently carried five interactions (light blue in the heatmap) and 22 participants consistently carried the same set of interactions (brown in the heatmap). For the rest, interaction occurrences were largely sporadic. The hierarchically organised rows led to identification of six clusters of participants (clusters 1–6), of which the cluster 1 was the largest (914 participants), and most had no or limited LRIs. The second largest cluster (cluster 2, 147 participants) appeared to carry five specific interactions (blue block in the heatmap) and cluster 5 (22 participants) carried seven specific interactions. Another large cluster with 72 participants seemed to uniquely carry the B*51:01–KIR2DS3 LRI.

Taking the six clusters obtained from the unsupervised learning, we computed and displayed the incidence curves for each cluster (Fig. 2c). Treating cluster 1 as the reference, we tested whether the incidence curve in each cluster was different from that in the reference group. The incidence curve of cluster 4 displayed a sharp increase from year 1 to year 2.5 and was significantly different from the reference (*p*=0.006), although there were only 16 participants. The incidence curve of cluster 2, with 147 participants, was generally higher than the reference incidence curve (*p*=0.003). Forty-four participants in cluster 3 seemed to experience higher incidence over years 0–5 than the reference group, although the two incidence curves crossed at around year 5 (*p*=0.39). On the other hand, the incidence curves for clusters 5 and 6 were consistently below the reference incidence curve, showing that individuals in these two clusters had significantly slower type 1 diabetes progression (*p*=0.04 and 0.05, respectively).

### Comparing predicted binding energies of KIR–HLA-I interfaces

Nine of the 14 KIRs on the heatmap shown in Fig. 2a had two receptor domains (D1 and D2) and are reported to bind HLA-A, -B, -C, or unknown ligands [30]. These properties are consistent with the template of two-domain KIR2DS2 bound to HLA-A*11:01 that produced more stable interactions as evaluated by PPCheck [31]. Of the other five KIRs on the heatmap, two are products of pseudogenes (*KIR2DP1* and *KIR3DP1*), one gene product is only known to bind HLA-G (KIR2DL4), and two are inhibitory three-domain KIRs (KIR3DL1 and KIR3DL2) for which binding may be influenced by their D0 domain [30]. This set of nine KIR2Ds forms 12 KIR2D–HLA-I interactions that are related to disease progression in the Fig. 2a heatmap.

PPCheck reported predicted binding energies partitioned into contributions from hydrogen bonds, electrostatics and van der Waals interactions for each modelled complex. For all models on all templates in this study and for six KIR–HLA-I crystal structures (the three templates and Protein Data Bank [PDB] IDs 1IM9, 1EFX, and 7K80; www.rcsb.org), the hydrogen bond contributes only about 5–20% of the total binding energy and is therefore not a determinant category overall. When we plotted the other two categories (predicted van der Waals energy vs electrostatic interactions) for the KIR–HLA-I complexes modelled on the 4N8V template, we observed that modelled complexes for KIR2D on the heatmap were calculated to be significantly stabilised by both types of interactions relative to other complexes (Fig. 2d). Above-mean binding energies are predicted for KIR2D heatmap complexes whether the associations with disease progression risk are positive or negative, and whether the KIRs are activating or inhibitory, with the exception of the KIR2DS4 complex with HLA-A*03:01 and the KIR2DL5 complex with HLA-B*37:01 (both of which are pink on the heatmap). Overall, these computational models and predictions suggest that type 1 diabetes progression risk (brown and blue on the heatmap) is associated with KIR–HLA-I complexes stabilised by electrostatic and van der Waals interactions encoded in the protein sequences, resulting in strong biophysical interactions between receptor and ligand that could alter the progression of disease.

## Discussion

The initial association analysis documented Limited HLA-I associations with type 1 diabetes progression. However, the KIR–HLA-I interaction analysis uncovered 22 interactions, with nine HLA-I (three *HLA-A* and six *HLA-B* allele products) and 14 KIRs affecting progression to type 1 diabetes (Table 1, Fig. 1 and ESM Fig. 1). Related homology models predicted that most KIR–HLA-I combinations associated with disease progression risk exhibited strong electrostatic and van der Waals interactions. For example, the activating receptor KIR2DS2 binds HLA-C*01:02 with six salt bridges [32] and the two activating receptors associated with type 1 diabetes progression, KIR2DS3 and KIR2DS5, are predicted to form four or five additional salt bridges with HLA-B*51:01 and HLA-A*25:01, respectively, substantially enhancing electrostatic binding energy. Similar receptors that may adopt a similar binding mode include the leukocyte immunoglobulin-like receptors, such as LILRB1, which are formed from two domains similar to KIRs and bind to a different surface of HLA-I molecules (i.e. at one edge of the antigen-binding groove), driven by ‘critical’ electrostatic interactions [33, 34]. Other well-studied inhibitory KIRs bind HLA-I molecules without as many favourable electrostatic interactions [35], for which association is likely driven by different types of interactions.

As the crystal structure of a two-domain KIR–HLA-B complex is not yet known, these homology models can be used to predict the nature of that interaction. All KIR2D–HLA-B complexes in the heatmap (with the exception of the HLA-G-specific KIR2DL4) are predicted to bind with a strong electrostatic interaction energy: their mean electrostatic component is −125 kJ/mol, compared with an overall electrostatic component of −90 kJ/mol for all 4N8V template models and −60 kJ/mol for six KIR–HLA-I crystal structures. Most HLA-B proteins contain the negatively charged αGlu76 in the middle of the binding footprint, possibly forming salt bridges with, for example, a distinctive positively charged Arg70 in KIR2DS3. The 4N8V template used in this study allows for an orientation of these residues that might strengthen the interaction and trigger disease progression. Interestingly, a very recent study has shown that in type 1 diabetes pancreatic lymph nodes there is a proportional increase in the frequency of cytotoxic CD56^dim^CD16^+^ NK cells, with an increase in the expression of NK cytotoxicity-related genes [36]. However, the role of KIR^+^ CD8^+^ regulatory T cells in the pathogenesis of type 1 diabetes has yet to be explored [37].

Despite insightful results and potential mechanisms, it is important to recognise that this is an exploratory investigation with modest *p* values and limited sample sizes associated with the respective interactions. Independent replication is necessary to validate these findings, which can then be used as a basis for further experimental studies. In addition, we did not stratify our analyses over sex or age because of limited sample sizes within groups. The LRIs discovered should be viewed as averaged associations with progression.

### Conclusions

A number of KIR–HLA-I interactions may be associated with evolution to stage 3 type 1 diabetes in stage 1/2 individuals. Such interactions could be important additions for predicting disease progression, as most risk prediction models to date have been developed for predicting disease susceptibility in the general population. In particular, individuals with significantly accelerated (cluster 2 and cluster 4) or deaccelerated (cluster 5 and cluster 6) progression may benefit from these predictions. Another implication from this study is the suggestion of additional roles for NK and CD8^+^ T cells in type 1 diabetes progression, thus opening new avenues for research on *KIR* and *HLA-I* genes.

## Supplementary Information

Below is the link to the electronic supplementary material.

Supplementary file1 (PDF 2.07 MB)

## Data Availability

Clinical data on participants in DPT-1 and TN07 can be obtained from the NIDDK-Central Repository (https://repository.niddk.nih.gov/home) following the formal approval process. Genotype data for *HLA* and *KIR* genes are available from the corresponding authors. Interested investigators are encouraged to contact the authors to collaborate.

## Abbreviations

DPT-1: Diabetes Prevention Trial-Type 1
HLA-I: HLA class I
HLA-II: HLA class II
iCohort: DPT-1 and TN07 participants, combined into an integrated cohort
KIR: Killer-cell immunoglobulin-like receptors
LD: Linkage disequilibrium
LRI: Ligand–receptor interaction
m1: Model 1
m2: Model 2
NGTS: Next-generation targeted sequencing
NK: Natural killer
TN07: Randomised Diabetes Prevention Trial with Oral Insulin sponsored by TrialNet
