# Correction: Profiling associations of interactive ligand–receptors (HLA class I and KIR gene products) with the progression to type 1 diabetes among seroconverted participants

**DOI:** 10.1007/s00125-025-06557-6

**Published:** 2025-09-27

**Authors:** Lue Ping Zhao, George K. Papadopoulos, Benjamin J. McFarland, Jay S. Skyler, Hemang M. Parikh, William W. Kwok, Terry P. Lybrand, George P. Bondinas, Antonis K. Moustakas, Ruihan Wang, Chul‑Woo Pyo, Wyatt C. Nelson, Daniel E. Geraghty, Åke Lernmark

**Affiliations:** 1 Public Health Sciences Division, Fred Hutchinson Cancer Research Centre, Seattle, WA USA; 2 School of Public Health, University of Washington, Seattle, WA USA; 3 Laboratory of Biophysics, Biochemistry, Biomaterials and Bioprocessing, Faculty of Agricultural Technology, Technological Educational Institute (TEI) of Epirus, Arta, Greece; 4 Department of Biochemistry, Seattle Pacific University, Seattle, USA; 5 Diabetes Research Institute and Division of Endocrinology, Diabetes & Metabolism, University of Miami Miler School of Medicine, Miami, Florida USA; 6 Health Informatics Institute, Morsani College of Medicine, University of South Florida, Tampa, FL USA; 7 Benaroya Research Institute, Seattle, WA USA; 8 Department of Chemistry, Vanderbilt University, Nashville, TN USA; 9 Department of Food Science and Technology, Faculty of Environmental Sciences, Ionian University, Argostoli, Cephalonia Greece; 10 Clinical Research Division, Fred Hutchinson Cancer Research Centre, Seattle, WA USA; 11 Department of Clinical Sciences, Lund University CRC, Skane University Hospital, Malmo, Sweden

**Correction: Diabetologia**

10.1007/s00125-025-06520-5

The trial ‘Randomised Diabetes Prevention Trial with Oral Insulin sponsored by TrialNet (TN07)’ was incorrectly attributed to the Immune Tolerance Network. The original article has been corrected.

