# Supplementary material for: Profiling associations of interactive ligand–receptors (HLA class I and KIR gene products) with the progression to type 1 diabetes among seroconverted participants
Source: Diabetologia. 2025 Aug 21;68(12):2743–53. doi: 10.1007/s00125-025-06520-5 (PMC12594725; doi:10.1007/s00125-025-06520-5)
Supplement: Supplementary file 1 — Supplementary file1 (PDF 2.07 MB) [file 125_2025_6520_MOESM1_ESM.pdf]

Online Supplementary Materials

Modeling KIR—HLA-I interactions and statistical evaluation

Given the focus on the KIR—HLA-I interactions, we expand the Cox regression to explicitly model these interactions. Consider J alleles of a *HLA-I* gene observed in the iCohort of n subjects, denoted as ($a_1, a_2, \ldots, a_J$). We create a covariate matrix ($I=||G_{ij}||_{n \text{ by } J}$), where $i=1,2,\ldots,n$ for the ith subject, and each G_{ij} takes a value of (0, 1, 2) for zero, one or two copies of the allele j, respectively. Further, let K_i be an indicator for presence or absence of KIR receptor observed on the ith subject. We model KIR—HLA-I interaction via the following Cox regression of the hazard function

$$\lambda(t, G_{ij}, K_i, C_i) = \lambda_0(t)\exp(\alpha_j G_{ij} + \beta_j K_i + \gamma_j G_{ij} K_i + C_i), \quad \text{[m1]}$$

in which $\lambda_0(t)$ is a nonparametrically estimated baseline hazard function. The term $\alpha_j G_{ij}$ is used to quantify possible main effect of the jth allele of an HLA-I gene over other alleles. The term $\beta_j K_i$, corresponding to main effect of a KIR, is used to quantify possible effect of the receptor. The critical HLA-I–KIR interactions are quantified by $\gamma_j G_{ij} K_i$, if the estimated γ_j does not equal zero. Hence, estimated $(\gamma_1, \gamma_2, \ldots, \gamma_J)$ coefficients, standard errors, Z-scores and p-values are used to report HLA-I–KIR interactions. Since prior information on main effects of ligands and receptors are limited, a conventional interaction analysis of (ligand, receptor) would prefer to estimate the interaction effect $\gamma_j G_{ij} K_i$, by isolating main effects of the ligand and receptor by estimating $\alpha_j G_{ij}$ and $\beta_j K_i$ via the model [m1], if computationally possible. Note that the analysis focuses on presence or absence of ligand and receptor for clarity and consistency in visualizing interaction effects, even though the above model [m1] allows us to carry out a quantitative analysis with number of ligands and number of receptors.

Due to excessive polymorphisms and high correlation of KIR and HLA-I, isolating main effects, when estimating interaction effects, becomes challenged for some of HLA-I–KIR pairs. In such cases, one could assume that ligand without receptor or receptor

without ligand have no association with the progression outcome, for the purpose of quantifying ligand-receptor interaction. This assumption would lead to absence of HLA-I and KIR main effects, i.e., $\alpha_j G_{ij} = 0$ and $\beta_j K_i = 0$, and corresponding model maybe written as

$$\lambda(t, G_{ij}, K_i, C_i) = \lambda_0(t)\exp(\gamma_j G_{ij} K_i + C_i). \qquad [m2]$$

Note that C_i is included in model [m1] and [m2] as an "offset" that captures confounding effects from multiple covariates, including HLA-II genes (*DQ, DR*), sources of study participants, age, sex, ethnicity, disease severity and treatment indicator. The offset is constructed from regressing time to T1D on all these confounding covariates and is then treated as a fixed variable to capture confounding effects across all association analyses, unless noted otherwise. Interaction analysis without or with C_i represents an unadjusted and adjusted interaction analysis, respectively.

When profiling HLA-I–KIR interactions, *p*-values are best viewed as "empirical quantification of interactions", due to variable numbers of comparisons concerning three HLA-I genes (*A, B, C*), variable alleles in each gene, and 15 *KIR* receptors. To provide a holistic evaluation of reproducibility in assessing HLA-I–KIR interactions, we consider two computational strategies: bootstrap for assessing stabilities of identified interactions, and permutation for assessing possibly inflated false error rates. To carry out a bootstrap analysis, we randomly select a sample of 1215 subjects from iCohort with replicates and repeat the whole KIR-HLA-I interaction analysis with estimated p-values to identify those interactions with p-values less than 0.05, especially recording those selected interactions. As we repeat the bootstrap process for, saying 100,000 time, we estimate frequencies that discovered KIR—HLA-I interactions have been repeated identified in all bootstrap samples. The greater the frequencies, the more stable discovered interactions will be. On the other hand, the permutation scheme is to permute progressions outcome randomly from genotypes, and then to carry out the whole HLA-I–KIR interaction analysis under the null hypothesis. Since there is no HLA-I–KIR interaction, any identified interactions are false discoveries. Repeating this permutation for, say, 100,000 times, we estimate frequencies that specific interactions are falsely discovered, i.e., false discovery rates in the context of existing data structures.

The HLA-I–KIR interaction analysis screens all KIR receptors, three HLA-I genes, and their individual alleles with over 1000 statistical tests, even though ligand-receptor interactions are well biologically motivated. Since conventional multiple comparison adjustments [1] are inevitably too conservative, we have chosen to report unadjusted p-values (Table 1 and 2). To assess stability of discovering HLA-I–KIR interactions, we carried out the bootstrap analysis to estimate frequencies that discovered interactions would be identified in 100,000 replications, and the permutation analysis to estimate frequencies that corresponding interactions could be identified in 100,000 permutations. Estimated permutation frequencies are around 0.04~0.06. By ratios of bootstrap over permutation frequencies, we assess the likelihood of discovered interactions over the chance finding, which provide empirical evidence in support of discovered interactions. Numerical assessment of bootstrap or permutation is not a replacement of independent replication study on these interactions.

During the computational screening with all HLA-I ligands and KIR receptors, less than 3% ligand-receptor pairs are deemed to have significant associations with the disease progression, leaving over 96% pairs to have negative associations. However, negative associations do not imply that such ligand-receptors are functionally significant, for the following reasons. First some of pairs have marginal associations with the progression, but associated sample sizes are too small to lead empirically observed associations with the progression. Second, some ligand-receptors are functionally important in the host immunity, but such functions are simply not impacting on the progression. For the rest, HLA-I molecules may not bind the KIR receptors. It is also important to remember that autoimmune reactions leading gradually to human T1D are probably initiated and propagated in the proximal lymph nodes with dendritic cells carrying beta cell autoantigens from the to be inspected by thereby circulating CD4 and CD8 T cells (Res 1a, 1b, 1h). The presence of $CD56^{bright}$ NK cells in human inguinal lymph nodes hints at another possibility of HLA-I–KIR interactions that might contribute T1D pathogenesis that must be also examined by immunohistochemistry/confocal microscopy besides flow cytometry [2].

Homology modeling and template selection for HLA-I–KIR complexes

HLA-I–KIR interactions, like TCR-MHC interactions, bind with moderate, micromolar-range affinities, which are mediated structurally by general regions of conserved biophysical compatibility, rather than by conserved individual sidechain interactions [3]. Because of this, longer-range electrostatic interactions, if present, are expected to be more important than shorter-range, more orientation-dependent hydrogen bond interactions. These considerations led to the choice of SWISS-MODEL for homology modeling because of its speed and demonstrated success in accuracy for ~85% identity ("lDDT easy") modeling tasks [4].

Inhibitory KIR receptors, especially those interacting with HLA-C, have been studied more than their activating counterparts and/or interactions with HLA-A and HLA-B [5]. There are currently two distinct crystal structures of activating HLA-I–KIR complexes: KIR2DS2 bound to HLA-A*11:01 [6] and HLA-C*01:02 [7]. These were selected as user templates for homology modelling, as well as an inhibitory KIR-HLA-B complex, KIR3DL1 bound to HLA-B*57:01-TW10 [8], to provide an example of a KIR bound to HLA-B. An additional D0-HLA interface is present in three-domain KIR sequences modeled on this three-domain template, which adds extraneous values to the energy interaction totals calculated by PPCheck [9]. These three templates (PDB IDs 4N8V for HLA-A, 7DUU for HLA-C, and 5T6Z for HLA-B, respectively) were used to generate three 3D protein models from each of the KIR and HLA sequences in the 22 KIR-HLA receptor-ligand complexes associated with progression risk and presented as a heat-map (see Figure 2.1). We also generated three 3D protein models (one for each template) for 19 additional HLA-I–KIR complexes not significantly associated with progression risk and not present on the heat-map. Peptides were added by structural alignment of the template KIR-HLA-peptide complex with the modeled HLA-KIR complex, then superimposing the crystal structure's peptide coordinates into the open groove of the model structure. When residue-residue interactions in these models were analyzed with PPCheck, the complexes modeled on the KIR2DS2-HLA-A (4N8V) template were predicted to have total interaction strengths stronger than or equal to (within <5%) two-domain models from other templates in 35 out of 41 cases. In addition, for the set of two-domain KIR models, complexes modeled on the 4N8V template on average scored better in 8 of 11 structural categories than those modeled on the other two templates. Since 11 of the 14 KIRs on the heat-map have two domains, the two-domain high-scoring 4N8V template best matched the majority of our dataset and was used for comparisons.

ESM Table 1. Demographic and clinical risk factors in the iCohort, computed frequencies, and association results include coefficient, hazard ratio, standard error, Z-score and p-value, from univariate analysis with one covariate a time (left panel) and multivariate analysis with all covariates (right panel). P-values, if less than 0.05, are highlighted brown and light blue, if corresponding HR is less than 1 or greater than 1, respectively.

			Univariate Analysis					Multivariate Analysis				
**Variable**	**Value**	**n**	**coef**	**HR**	**SE**	**Z**	**p**	**coef**	**HR**	**SE**	**Z**	**p**
**Race**	White	1097	0.00	1.00				0.00	1.00			
	Others	119	-0.03	0.97	0.18	-0.17	8.65E-01	0.07	1.07	0.18	0.37	7.09E-01
**Sex**	Male	708	0.00	1.00				0.00	1.00			
	Female	508	0.12	1.13	0.10	1.21	2.26E-01	0.14	1.16	0.10	1.44	1.50E-01
**Age**	in 10 years	1216	-0.36	0.70	0.01	-4.70	2.65E-06	-0.53	0.59	0.08	-6.33	2.44E-10
**Risk Level**	Low	900	0.00	1.00				0.00	1.00			
	High	316	0.61	1.83	0.08	5.82	5.92E-09	0.87	2.39	0.13	6.49	8.54E-11
**insulin**	Placebo	606	0.00	1.00				0.00	1.00			
	Tretment	610	-0.18	0.83	0.10	1.83	6.76E-02	0.17	1.19	0.10	1.76	7.90E-02
**Study**	DPT1	670	0.00	1.00				0.00	1.00			
	TN07	546	-0.18	0.83	0.10	-1.77	7.71E-02	0.00	1.00	0.13	-0.02	9.84E-01

ESM Table 2. Estimated haplotype frequencies of HLA-I genes (*A, B, C*) with *DRB1-DQA1-DQB1* haplotypes, in which *DR3* haplotypes are highlighted in light blue and *DR4* haplotypes in light brown.

DRB1-DQA1-DQB1-A	n	Freq	DRB1-DQA1-DQB1-B	n	Freq	DRB1-DQA1-DQB1-C	n	Freq
*01:01-*01:01-*05:01-A*02:01	31	0.013	*01:01-*01:01-*05:01-B*07:02	15	0.006	*01:01-*01:01-*05:01-C*01:02	25	0.01
*01:01-*01:01-*05:01-A*03:01	48	0.02	*01:01-*01:01-*05:01-B*27:05	16	0.007	*01:01-*01:01-*05:01-C*04:01	23	0.009
*01:01-*01:01-*05:01-A*24:02	13	0.005	*01:01-*01:01-*05:01-B*35:01	20	0.008	*01:01-*01:01-*05:01-C*05:01	11	0.005
*03:01-*05:01-*02:01-A*01:01	288	0.119	*01:01-*01:01-*05:01-B*44:02	12	0.005	*01:01-*01:01-*05:01-C*07:01	10	0.004
*03:01-*05:01-*02:01-A*02:01	118	0.049	*01:01-*01:01-*05:01-B*51:01	16	0.007	*01:01-*01:01-*05:01-C*07:02	27	0.011
*03:01-*05:01-*02:01-A*03:01	48	0.02	*01:02-*01:01-*05:01-B*14:02	16	0.007	*01:02-*01:01-*05:01-C*08:02	14	0.006
*03:01-*05:01-*02:01-A*11:01	13	0.005	*03:01-*05:01-*02:01-B*07:02	22	0.009	*03:01-*05:01-*02:01-C*03:04	16	0.007
*03:01-*05:01-*02:01-A*24:02	12	0.005	*03:01-*05:01-*02:01-B*08:01	386	0.159	*03:01-*05:01-*02:01-C*04:01	12	0.005
*03:01-*05:01-*02:01-A*30:02	32	0.013	*03:01-*05:01-*02:01-B*14:02	11	0.005	*03:01-*05:01-*02:01-C*05:01	82	0.034
*04:01-*03:01-*03:02-A*01:01	33	0.014	*03:01-*05:01-*02:01-B*18:01	69	0.028	*03:01-*05:01-*02:01-C*06:02	15	0.006
*04:01-*03:01-*03:02-A*02:01	347	0.143	*03:01-*05:01-*02:01-B*40:01	11	0.005	*03:01-*05:01-*02:01-C*07:01	384	0.158
*04:01-*03:01-*03:02-A*03:01	65	0.027	*03:01-*05:01-*02:01-B*44:02	16	0.007	*03:01-*05:01-*02:01-C*07:02	28	0.012
*04:01-*03:01-*03:02-A*11:01	25	0.01	*04:01-*03:01-*03:02-B*07:02	34	0.014	*03:01-*05:01-*02:01-C*08:02	13	0.005
*04:01-*03:01-*03:02-A*24:02	29	0.012	*04:01-*03:01-*03:02-B*08:01	16	0.007	*04:01-*03:01-*03:02-C*02:02	11	0.005
*04:01-*03:01-*03:02-A*25:01	17	0.007	*04:01-*03:01-*03:02-B*15:01	278	0.114	*04:01-*03:01-*03:02-C*03:03	104	0.043
*04:01-*03:01-*03:02-A*68:01	18	0.007	*04:01-*03:01-*03:02-B*18:01	35	0.014	*04:01-*03:01-*03:02-C*03:04	244	0.1
*04:01-*03:03-*03:01-A*01:01	15	0.006	*04:01-*03:01-*03:02-B*35:01	11	0.005	*04:01-*03:01-*03:02-C*04:01	21	0.009
*04:01-*03:03-*03:01-A*02:01	74	0.03	*04:01-*03:01-*03:02-B*40:01	49	0.02	*04:01-*03:01-*03:02-C*05:01	43	0.018
*04:01-*03:03-*03:02-A*02:01	36	0.015	*04:01-*03:01-*03:02-B*44:02	44	0.018	*04:01-*03:01-*03:02-C*06:02	13	0.005
*04:01-*03:03-*03:02-A*24:02	12	0.005	*04:01-*03:01-*03:02-B*51:01	25	0.01	*04:01-*03:01-*03:02-C*07:01	33	0.014
*04:02-*03:01-*03:02-A*02:01	10	0.004	*04:01-*03:01-*03:02-B*55:01	11	0.005	*04:01-*03:01-*03:02-C*07:02	31	0.013
*04:02-*03:01-*03:02-A*26:01	20	0.008	*04:01-*03:03-*03:01-B*40:01	12	0.005	*04:01-*03:01-*03:02-C*12:03	27	0.011
*04:04-*03:01-*03:02-A*01:01	11	0.005	*04:01-*03:03-*03:01-B*44:02	60	0.025	*04:01-*03:03-*03:01-C*03:04	16	0.007
*04:04-*03:01-*03:02-A*02:01	47	0.019	*04:01-*03:03-*03:02-B*27:05	24	0.01	*04:01-*03:03-*03:01-C*05:01	63	0.026
*04:04-*03:01-*03:02-A*03:01	31	0.013	*04:01-*03:03-*03:02-B*44:02	10	0.004	*04:01-*03:03-*03:02-C*02:02	18	0.007
*04:04-*03:01-*03:02-A*11:01	10	0.004	*04:02-*03:01-*03:02-B*35:01	11	0.005	*04:01-*03:03-*03:02-C*03:03	13	0.005
*04:04-*03:01-*03:02-A*24:02	24	0.01	*04:02-*03:01-*03:02-B*38:01	22	0.009	*04:02-*03:01-*03:02-C*04:01	11	0.005
*04:04-*03:01-*03:02-A*31:01	15	0.006	*04:04-*03:01-*03:02-B*07:02	28	0.012	*04:02-*03:01-*03:02-C*12:03	23	0.009
*04:04-*03:01-*03:02-A*68:01	10	0.004	*04:04-*03:01-*03:02-B*35:01	13	0.005	*04:04-*03:01-*03:02-C*03:04	47	0.019
*04:05-*03:03-*03:02-A*02:01	11	0.005	*04:04-*03:01-*03:02-B*40:01	44	0.018	*04:04-*03:01-*03:02-C*04:01	14	0.006
*04:05-*03:03-*03:02-A*02:05	12	0.005	*04:04-*03:01-*03:02-B*44:02	17	0.007	*04:04-*03:01-*03:02-C*05:01	11	0.005
*04:08-*03:03-*03:04-A*11:01	10	0.004	*04:05-*03:03-*03:02-B*49:01	20	0.008	*04:04-*03:01-*03:02-C*07:01	14	0.006
*07:01-*02:01-*02:02-A*02:01	15	0.006	*04:08-*03:03-*03:04-B*35:03	12	0.005	*04:04-*03:01-*03:02-C*07:02	31	0.013
*07:01-*02:01-*02:02-A*02:05	11	0.005	*07:01-*02:01-*02:02-B*13:02	19	0.008	*04:05-*03:03-*03:02-C*07:01	20	0.008
*07:01-*02:01-*02:02-A*03:01	16	0.007	*07:01-*02:01-*02:02-B*44:03	34	0.014	*04:07-*03:01-*03:02-C*07:02	13	0.005
*07:01-*02:01-*02:02-A*29:02	25	0.01	*07:01-*02:01-*02:02-B*50:01	14	0.006	*04:08-*03:03-*03:04-C*12:03	13	0.005
*08:01-*04:01-*04:02-A*02:01	18	0.007	*13:01-*01:03-*06:03-B*44:02	14	0.006	*07:01-*02:01-*02:02-C*04:01	11	0.005
*09:01-*03:02-*03:03-A*02:01	10	0.004	*13:02-*01:02-*06:04-B*40:01	40	0.016	*07:01-*02:01-*02:02-C*06:02	37	0.015
*11:01-*05:05-*03:01-A*03:01	12	0.005	Rare haplotypes (<10 copies)	923	0.38	*07:01-*02:01-*02:02-C*08:02	10	0.004
*13:01-*01:03-*06:03-A*02:01	21	0.009				*07:01-*02:01-*02:02-C*16:01	29	0.012
*13:01-*01:03-*06:03-A*03:01	14	0.006				*08:01-*04:01-*04:02-C*07:02	16	0.007
*13:01-*01:03-*06:03-A*24:02	14	0.006				*08:02-*07:01-*02:01-*02:02	10	0.004
*13:02-*01:02-*06:04-A*01:01	12	0.005				*13:01-*01:03-*06:03-C*03:03	10	0.004
*13:02-*01:02-*06:04-A*02:01	48	0.02				*13:01-*01:03-*06:03-C*05:01	12	0.005
Rare haplotypes (<10 copies)	729	0.3				*13:02-*01:02-*06:04-C*03:04	41	0.017
						*13:02-*01:02-*06:04-C*07:01	10	0.004
						*13:02-*01:02-*06:04-C*07:02	10	0.004
						Rare haplotypes (<10 copies)	738	0.304

ESM Table 3. Allelic associations of HLA-A with the disease progression: allelic frequency, estimated coefficient, hazard ratio, standard error, Z-score and p-value without (left panel) and with (right panel) adjustment of HLA-II and other confounding variables. P-values, if less than 0.05, are highlighted brown and light blue, if corresponding HR is less than 1 or greater than 1, respectively.

seq	HLA-A	n	Unadjusted					Adjusted				
			coef	HR	SE	z	p	coef	HR	SE	z	p
1	A*01:01	420	-0.02	0.98	0.09	-0.22	8.27E-01	-0.05	0.95	0.09	-0.53	5.98E-01
2	A*02:01	842	-0.15	0.86	0.08	-1.97	4.83E-02	-0.13	0.88	0.08	-1.78	7.59E-02
3	A*02:05	40	-0.29	0.75	0.34	-0.86	3.89E-01	-0.36	0.70	0.34	-1.08	2.80E-01
4	A*02:06	17	-0.95	0.39	0.71	-1.34	1.80E-01	-0.83	0.44	0.71	-1.17	2.42E-01
5	A*03:01	292	0.06	1.06	0.11	0.55	5.83E-01	0.09	1.09	0.11	0.81	4.21E-01
6	A*11:01	96	0.17	1.19	0.17	1.02	3.06E-01	0.07	1.07	0.17	0.40	6.86E-01
7	A*23:01	31	0.16	1.17	0.32	0.48	6.28E-01	0.28	1.33	0.32	0.88	3.79E-01
8	A*24:02	180	0.24	1.27	0.13	1.86	6.27E-02	0.35	1.42	0.12	2.83	4.71E-03
9	A*25:01	44	0.20	1.22	0.24	0.82	4.12E-01	0.12	1.13	0.24	0.50	6.20E-01
10	A*26:01	61	-0.17	0.84	0.23	-0.74	4.59E-01	-0.27	0.76	0.23	-1.18	2.38E-01
11	A*29:02	66	0.38	1.46	0.20	1.85	6.49E-02	0.28	1.32	0.21	1.34	1.80E-01
12	A*30:01	18	-0.12	0.89	0.45	-0.27	7.87E-01	0.03	1.03	0.45	0.06	9.55E-01
13	A*30:02	41	0.14	1.15	0.26	0.56	5.75E-01	-0.05	0.95	0.26	-0.18	8.56E-01
14	A*31:01	42	0.10	1.10	0.25	0.39	6.95E-01	-0.13	0.88	0.25	-0.53	5.98E-01
15	A*32:01	48	-0.43	0.65	0.32	-1.34	1.80E-01	-0.11	0.90	0.32	-0.33	7.43E-01
16	A*33:01	13	0.49	1.63	0.38	1.28	2.00E-01	0.41	1.51	0.38	1.07	2.82E-01
17	A*68:01	70	-0.04	0.96	0.20	-0.20	8.41E-01	0.10	1.11	0.20	0.51	6.12E-01
18	A*68:02	26	-0.12	0.89	0.36	-0.33	7.44E-01	0.05	1.05	0.36	0.14	8.85E-01
	rare (<10)	81	0.16	1.17	0.18	0.88	3.79E-01	0.07	1.07	0.18	0.39	6.99E-01

ESM Table 4. Allelic associations of HLA-B with the disease progression: allelic frequency, estimated coefficient, hazard ratio, standard error, Z-score and p-value without (left panel) and with (right panel) adjustment of HLA-II and other confounding variables. P-values, if less than 0.05, are highlighted brown and light blue, if corresponding HR is less than 1 or greater than 1, respectively.

			Unadjusted					Adjusted				
seq	HLA-B	n	coef	HR	SE	z	p	coef	HR	SE	z	p
1	B*07:02	158	-0.08	0.93	0.15	-0.52	6.04E-01	-0.14	0.87	0.15	-0.94	3.45E-01
2	B*08:01	437	0.10	1.10	0.09	1.07	2.85E-01	0.06	1.06	0.09	0.62	5.33E-01
3	B*13:02	30	-0.24	0.78	0.36	-0.69	4.93E-01	0.44	1.55	0.36	1.22	2.23E-01
4	B*14:01	16	-0.09	0.92	0.45	-0.19	8.46E-01	-0.32	0.73	0.45	-0.71	4.81E-01
5	B*14:02	51	-0.01	0.99	0.26	-0.02	9.81E-01	-0.11	0.90	0.26	-0.42	6.77E-01
6	B*15:01	328	-0.02	0.98	0.10	-0.23	8.22E-01	-0.14	0.87	0.10	-1.37	1.71E-01
7	B*15:18	12	0.45	1.56	0.50	0.89	3.74E-01	0.77	2.17	0.50	1.54	1.24E-01
8	B*18:01	153	0.42	1.52	0.13	3.27	1.07E-03	0.19	1.21	0.13	1.50	1.35E-01
9	B*27:05	72	-0.15	0.86	0.20	-0.75	4.51E-01	-0.11	0.89	0.20	-0.56	5.76E-01
10	B*35:01	85	-0.05	0.95	0.18	-0.27	7.88E-01	0.07	1.07	0.18	0.39	6.94E-01
11	B*35:03	32	0.11	1.11	0.26	0.40	6.89E-01	0.04	1.04	0.27	0.16	8.76E-01
12	B*35:08	11	0.27	1.31	0.45	0.60	5.47E-01	0.51	1.67	0.45	1.14	2.56E-01
13	B*37:01	22	-1.01	0.36	0.58	-1.74	8.18E-02	-1.25	0.29	0.58	-2.15	3.12E-02
14	B*38:01	44	0.16	1.17	0.21	0.75	4.51E-01	-0.01	0.99	0.22	-0.05	9.57E-01
15	B*39:01	28	-0.05	0.95	0.34	-0.15	8.81E-01	0.51	1.66	0.34	1.50	1.35E-01
16	B*39:06	34	0.12	1.13	0.27	0.44	6.63E-01	0.22	1.25	0.27	0.81	4.17E-01
17	B*40:01	187	0.04	1.04	0.12	0.29	7.72E-01	0.09	1.10	0.12	0.76	4.50E-01
18	B*40:02	28	-0.78	0.46	0.50	-1.55	1.20E-01	-0.07	0.93	0.51	-0.15	8.83E-01
19	B*41:01	11	-0.10	0.90	0.58	-0.18	8.59E-01	0.04	1.04	0.58	0.07	9.42E-01
20	B*44:02	201	-0.09	0.91	0.13	-0.71	4.80E-01	0.09	1.10	0.13	0.69	4.93E-01
21	B*44:03	57	0.08	1.09	0.23	0.36	7.20E-01	0.13	1.14	0.23	0.56	5.72E-01
22	B*45:01	17	-0.14	0.87	0.50	-0.27	7.87E-01	-0.46	0.63	0.50	-0.91	3.64E-01
23	B*49:01	27	0.32	1.37	0.36	0.89	3.75E-01	0.21	1.23	0.36	0.58	5.65E-01
24	B*50:01	31	0.24	1.27	0.31	0.78	4.34E-01	0.03	1.03	0.31	0.10	9.24E-01
25	B*51:01	101	-0.36	0.70	0.21	-1.74	8.20E-02	-0.31	0.73	0.21	-1.50	1.33E-01
26	B*52:01	12	-0.50	0.61	0.71	-0.70	4.83E-01	0.12	1.13	0.71	0.17	8.66E-01
27	B*53:01	12	0.31	1.36	0.50	0.61	5.39E-01	0.26	1.29	0.50	0.51	6.10E-01
28	B*55:01	41	-0.02	0.98	0.28	-0.07	9.44E-01	0.03	1.03	0.28	0.11	9.09E-01
29	B*56:01	17	-0.15	0.86	0.50	-0.30	7.61E-01	0.55	1.74	0.50	1.09	2.74E-01
30	B*57:01	21	-0.11	0.89	0.41	-0.27	7.86E-01	0.05	1.05	0.41	0.13	8.98E-01
31	B*58:01	12	-0.02	0.98	0.44	-0.04	9.65E-01	-0.06	0.94	0.43	-0.14	8.92E-01
	rare (<10)	140	-0.16	0.85	0.16	-1.03	3.02E-01	-0.14	0.87	0.16	-0.86	3.88E-01

ESM Table 5. Allelic associations of HLA-C with the disease progression: allelic frequency, estimated coefficient, hazard ratio, standard error, Z-score and p-value without (left panel) and with (right panel) adjustment of HLA-I, HLA-II and other confounding variables. P-values, if less than 0.05, are highlighted brown and light blue, if corresponding HR is less than 1 or greater than 1, respectively.

			Unadjusted					Adjusted				
seq	HLA-C	n	coef	HR	SE	z	p	coef	HR	SE	z	p
1	C*01:02	75	-0.24	0.78	0.21	-1.14	2.53E-01	0.00	1.00	0.22	0.00	9.97E-01
2	C*02:02	82	-0.45	0.64	0.24	-1.91	5.58E-02	-0.36	0.70	0.24	-1.49	1.37E-01
3	C*03:03	161	0.04	1.04	0.14	0.28	7.76E-01	-0.03	0.97	0.14	-0.22	8.24E-01
4	C*03:04	398	-0.01	0.99	0.09	-0.10	9.24E-01	-0.05	0.95	0.09	-0.55	5.81E-01
5	C*04:01	146	-0.17	0.84	0.14	-1.22	2.21E-01	-0.09	0.91	0.15	-0.61	5.40E-01
6	C*05:01	255	0.06	1.06	0.11	0.54	5.87E-01	-0.02	0.98	0.11	-0.22	8.27E-01
7	C*06:02	131	-0.08	0.93	0.17	-0.46	6.43E-01	-0.12	0.89	0.17	-0.71	4.80E-01
8	C*07:01	517	0.14	1.15	0.09	1.60	1.11E-01	0.12	1.13	0.09	1.38	1.67E-01
9	C*07:02	220	-0.07	0.93	0.12	-0.59	5.57E-01	0.01	1.01	0.13	0.08	9.39E-01
10	C*07:04	36	-0.09	0.92	0.31	-0.29	7.75E-01	0.28	1.32	0.31	0.91	3.64E-01
11	C*08:02	64	-0.01	0.99	0.23	-0.03	9.80E-01	-0.13	0.88	0.23	-0.58	5.62E-01
12	C*12:02	11	-0.39	0.68	0.71	-0.54	5.86E-01	0.16	1.17	0.71	0.22	8.24E-01
13	C*12:03	116	0.32	1.37	0.14	2.30	2.16E-02	0.22	1.24	0.14	1.52	1.29E-01
14	C*14:02	26	0.14	1.15	0.34	0.41	6.82E-01	0.17	1.18	0.34	0.49	6.25E-01
15	C*15:02	48	-0.46	0.63	0.28	-1.65	9.83E-02	-0.25	0.78	0.28	-0.89	3.72E-01
16	C*15:05	13	-0.27	0.77	0.50	-0.53	5.96E-01	-0.69	0.50	0.50	-1.37	1.70E-01
17	C*16:01	55	0.01	1.01	0.25	0.05	9.58E-01	0.04	1.04	0.25	0.17	8.61E-01
18	C*16:02	11	0.61	1.83	0.41	1.47	1.41E-01	0.59	1.81	0.41	1.44	1.50E-01
	rare (<10)	63	0.11	1.12	0.21	0.52	6.03E-01	0.11	1.12	0.21	0.54	5.87E-01

ESM Table 6. Ligand associations of KIR genes with the disease progression: ligand numbers and their frequency, estimated coefficient, hazard ratio, standard error, Z-score and p-value without (left panel) and with (right panel) adjustment of HLA-I, HLA-II and other confounding variables

seq	KIR	receptor number|frequency	Unadjusted					Adjusted				
			coef	HR	SE	z	p	coef	HR	SE	z	p
1	KIR2DL1	0|30, 1|365, 2|800, 3|13	-0.06	0.94	0.09	-0.69	4.93E-01	-0.03	0.97	0.09	-0.30	7.66E-01
2	KIR2DL2	0|607, 1|495, 2|106	0.03	1.03	0.08	0.39	7.00E-01	0.02	1.02	0.08	0.25	8.03E-01
3	KIR2DL3	0|108, 1|503, 2|597	-0.04	0.96	0.08	-0.53	5.98E-01	-0.03	0.97	0.08	-0.43	6.66E-01
4	KIR2DL4	1|38, 2|1132, 3|38	-0.24	0.79	0.20	-1.18	2.38E-01	0.03	1.04	0.19	0.18	8.59E-01
5	KIR2DL5	0|615, 1|406, 2|150, 3|35, 4|2	0.05	1.06	0.06	0.89	3.75E-01	0.06	1.06	0.06	0.90	3.68E-01
6	KIR2DS1	0|748, 1|404, 2|56	0.11	1.12	0.08	1.33	1.84E-01	0.11	1.11	0.08	1.25	2.13E-01
7	KIR2DS2	0|600, 1|502, 2|106	0.04	1.04	0.08	0.57	5.67E-01	0.04	1.04	0.08	0.56	5.73E-01
8	KIR2DS3	0|890, 1|244, 2|70, 3|2, 4|2	0.02	1.02	0.08	0.25	8.04E-01	0.02	1.02	0.08	0.21	8.36E-01
9	KIR2DS4	0|57, 1|408, 2|743	-0.10	0.90	0.08	-1.22	2.23E-01	-0.10	0.91	0.09	-1.14	2.53E-01
10	KIR2DS5	0|827, 1|343, 2|37, 3|1	0.11	1.11	0.09	1.14	2.54E-01	0.10	1.11	0.09	1.12	2.63E-01
11	KIR3DL1	0|56, 1|404, 2|748	-0.11	0.90	0.08	-1.26	2.06E-01	-0.10	0.90	0.09	-1.17	2.41E-01
12	KIR3DL2	1|14, 2|1193, 3|1	0.28	1.32	0.55	0.50	6.16E-01	0.30	1.35	0.54	0.56	5.77E-01
13	KIR3DS1	0|763, 1|377, 2|67, 3|1	0.06	1.07	0.08	0.77	4.42E-01	0.11	1.11	0.08	1.25	2.10E-01
14	KIR2DP1	0|28, 1|358, 2|822	-0.06	0.94	0.09	-0.67	5.05E-01	-0.04	0.96	0.09	-0.38	7.03E-01
15	KIR3DP1	1|39, 2|1132, 3|37	-0.21	0.81	0.20	-1.06	2.91E-01	0.06	1.06	0.19	0.32	7.48E-01

ESM Table 7. Interaction of HLA-A with KIR ligand numbers with the disease progression without (left panel) and with (right panel) adjustment for confounding effects: allelic frequency (left panel) or interactive frequency (right panel), estimated coefficient, hazard ratio, standard error, Z-score and p-value. P-values, if less than 0.05, are highlighted brown and light blue, if corresponding HR is less than 1 or greater than 1, respectively.

seq	A-KIR-model	Eff. Size		Unadjusted					Adjusted				
		n0	n1	coef	HR	SE	z	p	coef	HR	SE	z	p
1	A*01:01-KIR2DL1-m2	251	121	-0.01	0.99	0.11	-0.11	9.15E-01	-0.06	0.94	0.11	-0.55	5.79E-01
2	A*02:01-KIR2DL1-m2	459	224	-0.17	0.84	0.10	-1.73	8.34E-02	-0.16	0.85	0.10	-1.64	1.01E-01
3	A*02:05-KIR2DL1-m2	29	8	-0.34	0.71	0.36	-0.94	3.46E-01	-0.43	0.65	0.36	-1.19	2.34E-01
4	A*02:06-KIR2DL1-m2	14	2	-0.93	0.40	0.71	-1.31	1.91E-01	-0.82	0.44	0.71	-1.16	2.48E-01
5	A*03:01-KIR2DL1-m2	171	95	0.07	1.07	0.12	0.60	5.50E-01	0.07	1.07	0.12	0.57	5.71E-01
6	A*11:01-KIR2DL1-m2	57	36	0.26	1.29	0.18	1.46	1.43E-01	0.13	1.14	0.18	0.74	4.56E-01
7	A*23:01-KIR2DL1-m2	20	10	0.18	1.19	0.32	0.55	5.80E-01	0.31	1.36	0.32	0.96	3.35E-01
8	A*24:02-KIR2DL1-m2	108	64	0.23	1.25	0.14	1.66	9.64E-02	0.38	1.47	0.14	2.80	5.18E-03
9	A*25:01-KIR2DL1-m2	26	17	0.17	1.19	0.25	0.70	4.84E-01	0.14	1.15	0.25	0.56	5.77E-01
10	A*26:01-KIR2DL1-m2	41	16	-0.26	0.77	0.26	-1.00	3.16E-01	-0.29	0.75	0.26	-1.13	2.58E-01
11	A*29:02-KIR2DL1-m2	37	20	0.34	1.41	0.23	1.48	1.39E-01	0.27	1.31	0.23	1.18	2.38E-01
12	A*30:01-KIR2DL1-m2	13	5	-0.12	0.89	0.45	-0.27	7.87E-01	0.03	1.03	0.45	0.07	9.46E-01
13	A*30:02-KIR2DL1-m2	25	15	0.11	1.11	0.26	0.40	6.87E-01	-0.07	0.93	0.26	-0.27	7.87E-01
14	A*31:01-KIR2DL1-m2	25	17	0.10	1.10	0.25	0.39	6.99E-01	-0.13	0.88	0.25	-0.53	5.99E-01
15	A*32:01-KIR2DL1-m2	37	10	-0.41	0.66	0.32	-1.28	1.99E-01	-0.10	0.91	0.32	-0.30	7.63E-01
16	A*33:01-KIR2DL1-m2	4	7	0.72	2.06	0.38	1.90	5.77E-02	0.58	1.79	0.38	1.53	1.26E-01
17	A*68:01-KIR2DL1-m2	42	25	0.01	1.01	0.21	0.06	9.54E-01	0.13	1.13	0.21	0.61	5.44E-01
18	A*68:02-KIR2DL1-m2	18	8	-0.12	0.89	0.36	-0.33	7.42E-01	0.05	1.05	0.36	0.14	8.88E-01
19	R10-KIR2DL1-m1	46	27	-0.75	0.47	1.07	-0.70	4.86E-01	-1.15	0.32	1.07	-1.07	2.83E-01
20	A*01:01-KIR2DL2-m2	126	62	0.00	1.00	0.14	0.03	9.80E-01	-0.06	0.94	0.14	-0.46	6.46E-01

21	A*02:01-KIR2DL2-m2	230	113	-0.10	0.91	0.11	-0.89	3.74E-01	-0.09	0.91	0.11	-0.85	3.93E-01
22	A*02:05-KIR2DL2-m2	17	4	-0.38	0.68	0.50	-0.76	4.47E-01	-0.52	0.60	0.50	-1.03	3.04E-01
23	A*02:06-KIR2DL2-m2	10	2	-0.40	0.67	0.71	-0.56	5.77E-01	-0.15	0.86	0.71	-0.21	8.36E-01
24	A*03:01-KIR2DL2-m1	95	48	-0.19	0.83	0.23	-0.81	4.19E-01	-0.15	0.86	0.23	-0.63	5.29E-01
25	A*11:01-KIR2DL2-m2	32	20	0.32	1.38	0.23	1.40	1.61E-01	0.14	1.15	0.23	0.59	5.56E-01
26	A*23:01-KIR2DL2-m2	13	6	0.12	1.13	0.41	0.29	7.70E-01	0.37	1.45	0.41	0.90	3.66E-01
27	A*24:02-KIR2DL2-m1	54	29	-0.28	0.75	0.27	-1.03	3.04E-01	-0.33	0.72	0.27	-1.22	2.22E-01
28	A*25:01-KIR2DL2-m1	14	9	-0.16	0.85	0.48	-0.34	7.35E-01	-0.13	0.88	0.48	-0.27	7.88E-01
29	A*26:01-KIR2DL2-m1	24	11	0.17	1.18	0.48	0.35	7.24E-01	0.28	1.32	0.48	0.58	5.61E-01
30	A*29:02-KIR2DL2-m2	20	12	0.46	1.59	0.29	1.58	1.13E-01	0.18	1.20	0.29	0.63	5.31E-01
31	A*30:01-KIR2DL2-m1	4	1	0.39	1.47	1.12	0.34	7.31E-01	1.48	4.38	1.13	1.31	1.90E-01
32	A*30:02-KIR2DL2-m1	9	10	0.42	1.53	0.53	0.81	4.20E-01	0.11	1.12	0.53	0.22	8.29E-01
33	A*31:01-KIR2DL2-m1	9	9	0.59	1.81	0.50	1.20	2.31E-01	0.66	1.93	0.50	1.32	1.86E-01
34	A*32:01-KIR2DL2-m1	18	6	0.48	1.61	0.65	0.73	4.66E-01	0.30	1.35	0.65	0.46	6.46E-01
35	A*33:01-KIR2DL2-m2	2	2	0.84	2.32	0.71	1.19	2.35E-01	0.79	2.19	0.71	1.11	2.68E-01
36	A*68:01-KIR2DL2-m1	23	10	-0.43	0.65	0.42	-1.03	3.04E-01	-0.20	0.82	0.42	-0.46	6.43E-01
37	A*68:02-KIR2DL2-m2	8	5	0.12	1.12	0.45	0.26	7.95E-01	0.08	1.09	0.45	0.18	8.56E-01
38	R10-KIR2DL2-m2	25	18	0.24	1.27	0.24	1.00	3.16E-01	0.07	1.08	0.24	0.30	7.61E-01
39	A*01:01-KIR2DL3-m2	238	113	-0.03	0.97	0.11	-0.27	7.84E-01	-0.08	0.93	0.11	-0.70	4.84E-01
40	A*02:01-KIR2DL3-m2	430	210	-0.15	0.86	0.10	-1.50	1.34E-01	-0.14	0.87	0.10	-1.40	1.60E-01
41	A*02:05-KIR2DL3-m2	26	7	-0.32	0.73	0.38	-0.84	4.04E-01	-0.52	0.59	0.38	-1.36	1.73E-01
42	A*02:06-KIR2DL3-m1	13	1	-1.72	0.18	1.42	-1.21	2.26E-01	-2.07	0.13	1.42	-1.45	1.46E-01
43	A*03:01-KIR2DL3-m1	163	87	-0.52	0.59	0.39	-1.35	1.76E-01	-0.24	0.79	0.39	-0.62	5.36E-01
44	A*11:01-KIR2DL3-m1	50	35	1.51	4.55	1.03	1.47	1.41E-01	1.36	3.90	1.03	1.32	1.86E-01
45	A*23:01-KIR2DL3-m1	18	8	-0.44	0.64	0.81	-0.55	5.84E-01	-0.65	0.52	0.81	-0.80	4.22E-01
46	A*24:02-KIR2DL3-m2	103	59	0.20	1.22	0.14	1.40	1.60E-01	0.35	1.42	0.14	2.47	1.36E-02
47	A*25:01-KIR2DL3-m1	24	15	-0.84	0.43	0.66	-1.28	2.00E-01	-0.39	0.67	0.66	-0.60	5.49E-01
48	A*26:01-KIR2DL3-m1	35	15	0.13	1.13	0.59	0.21	8.31E-01	0.50	1.65	0.59	0.84	3.98E-01
49	A*29:02-KIR2DL3-m2	36	19	0.37	1.45	0.24	1.59	1.13E-01	0.30	1.35	0.24	1.26	2.09E-01
50	A*30:01-KIR2DL3-m2	12	4	-0.31	0.74	0.50	-0.61	5.42E-01	-0.19	0.83	0.50	-0.38	7.07E-01
51	A*30:02-KIR2DL3-m1	24	14	-0.39	0.68	0.78	-0.50	6.15E-01	-1.02	0.36	0.78	-1.32	1.87E-01

52	A*31:01-KIR2DL3-m2	25	15	0.02	1.02	0.26	0.07	9.44E-01	-0.22	0.80	0.26	-0.82	4.11E-01
53	A*32:01-KIR2DL3-m1	34	9	0.01	1.01	1.07	0.01	9.93E-01	-0.57	0.57	1.07	-0.53	5.94E-01
54	A*33:01-KIR2DL3-m2	4	6	0.75	2.11	0.41	1.81	7.04E-02	0.55	1.73	0.41	1.33	1.83E-01
55	A*68:01-KIR2DL3-m2	38	23	0.05	1.05	0.21	0.23	8.19E-01	0.11	1.12	0.21	0.53	5.93E-01
56	A*68:02-KIR2DL3-m2	16	8	-0.03	0.97	0.36	-0.07	9.41E-01	0.14	1.15	0.36	0.39	6.99E-01
57	R10-KIR2DL3-m2	42	27	0.23	1.25	0.20	1.13	2.59E-01	0.09	1.09	0.20	0.45	6.54E-01
58	A*01:01-KIR2DL4-m2	258	125	-0.02	0.98	0.11	-0.21	8.37E-01	-0.05	0.95	0.11	-0.51	6.10E-01
59	A*02:01-KIR2DL4-m2	470	228	-0.18	0.83	0.10	-1.82	6.90E-02	-0.16	0.85	0.10	-1.63	1.03E-01
60	A*02:05-KIR2DL4-m2	31	8	-0.41	0.67	0.36	-1.14	2.54E-01	-0.48	0.62	0.36	-1.34	1.80E-01
61	A*02:06-KIR2DL4-m2	15	2	-0.95	0.39	0.71	-1.34	1.80E-01	-0.83	0.44	0.71	-1.17	2.41E-01
62	A*03:01-KIR2DL4-m2	174	97	0.08	1.09	0.12	0.70	4.84E-01	0.07	1.08	0.12	0.64	5.24E-01
63	A*11:01-KIR2DL4-m2	58	36	0.23	1.26	0.18	1.31	1.90E-01	0.11	1.12	0.18	0.63	5.32E-01
64	A*23:01-KIR2DL4-m2	21	10	0.16	1.17	0.32	0.49	6.27E-01	0.28	1.32	0.32	0.88	3.81E-01
65	A*24:02-KIR2DL4-m2	111	64	0.21	1.23	0.14	1.52	1.28E-01	0.38	1.46	0.14	2.77	5.56E-03
66	A*25:01-KIR2DL4-m2	26	18	0.20	1.22	0.24	0.82	4.14E-01	0.12	1.13	0.24	0.50	6.17E-01
67	A*26:01-KIR2DL4-m2	41	19	-0.15	0.86	0.24	-0.65	5.14E-01	-0.25	0.78	0.24	-1.05	2.93E-01
68	A*29:02-KIR2DL4-m2	41	22	0.33	1.40	0.22	1.52	1.30E-01	0.24	1.27	0.22	1.08	2.80E-01
69	A*30:01-KIR2DL4-m2	13	5	-0.12	0.89	0.45	-0.27	7.87E-01	0.03	1.03	0.45	0.07	9.46E-01
70	A*30:02-KIR2DL4-m2	25	16	0.14	1.15	0.26	0.56	5.79E-01	-0.05	0.96	0.26	-0.18	8.57E-01
71	A*31:01-KIR2DL4-m2	25	17	0.10	1.10	0.25	0.39	6.99E-01	-0.13	0.88	0.25	-0.53	5.99E-01
72	A*32:01-KIR2DL4-m2	38	10	-0.43	0.65	0.32	-1.34	1.79E-01	-0.11	0.90	0.32	-0.34	7.35E-01
73	A*33:01-KIR2DL4-m2	5	7	0.67	1.95	0.38	1.75	8.08E-02	0.56	1.75	0.38	1.46	1.45E-01
74	A*68:01-KIR2DL4-m2	43	25	0.00	1.00	0.21	0.02	9.85E-01	0.12	1.12	0.21	0.56	5.77E-01
75	A*68:02-KIR2DL4-m2	18	8	-0.12	0.89	0.36	-0.33	7.42E-01	0.05	1.05	0.36	0.14	8.88E-01
76	R10-KIR2DL4-m2	48	28	0.19	1.21	0.20	0.98	3.28E-01	0.10	1.11	0.20	0.53	5.93E-01
77	A*01:01-KIR2DL5-m1	128	63	0.05	1.06	0.22	0.25	8.02E-01	0.14	1.15	0.22	0.64	5.20E-01
78	A*02:01-KIR2DL5-m1	224	111	-0.10	0.91	0.20	-0.48	6.29E-01	-0.23	0.80	0.20	-1.13	2.57E-01
79	A*02:05-KIR2DL5-m1	14	6	0.99	2.70	0.82	1.21	2.27E-01	0.95	2.58	0.82	1.15	2.50E-01
80	A*02:06-KIR2DL5-m2	8	2	-0.38	0.69	0.71	-0.53	5.94E-01	-0.12	0.88	0.71	-0.17	8.61E-01
81	A*03:01-KIR2DL5-m2	79	49	0.18	1.20	0.15	1.21	2.26E-01	0.20	1.23	0.15	1.34	1.82E-01
82	A*11:01-KIR2DL5-m2	31	18	0.15	1.16	0.24	0.63	5.27E-01	0.09	1.09	0.24	0.37	7.09E-01

83	A*23:01-KIR2DL5-m1	13	5	-0.24	0.79	0.64	-0.38	7.07E-01	-0.55	0.58	0.64	-0.86	3.90E-01
84	A*24:02-KIR2DL5-m2	52	39	0.34	1.40	0.17	1.99	4.62E-02	0.50	1.65	0.17	2.97	3.00E-03
85	A*25:01-KIR2DL5-m2	13	8	0.25	1.28	0.36	0.70	4.84E-01	0.13	1.14	0.36	0.37	7.15E-01
86	A*26:01-KIR2DL5-m2	21	10	-0.16	0.85	0.32	-0.51	6.09E-01	-0.32	0.72	0.32	-1.01	3.14E-01
87	A*29:02-KIR2DL5-m2	17	8	0.16	1.18	0.36	0.46	6.46E-01	0.17	1.18	0.36	0.46	6.42E-01
88	A*30:01-KIR2DL5-m1	6	1	-1.00	0.37	1.12	-0.89	3.73E-01	-0.22	0.80	1.13	-0.20	8.42E-01
89	A*30:02-KIR2DL5-m1	11	9	-0.02	0.98	0.51	-0.03	9.76E-01	0.32	1.37	0.51	0.62	5.38E-01
90	A*31:01-KIR2DL5-m1	13	12	0.44	1.56	0.54	0.81	4.15E-01	0.19	1.21	0.54	0.36	7.22E-01
91	A*32:01-KIR2DL5-m1	16	7	0.79	2.20	0.70	1.13	2.58E-01	0.81	2.26	0.70	1.17	2.43E-01
92	A*33:01-KIR2DL5-m1	4	3	-0.61	0.54	0.77	-0.79	4.28E-01	-0.51	0.60	0.77	-0.67	5.06E-01
93	A*68:01-KIR2DL5-m1	24	11	-0.57	0.56	0.42	-1.37	1.70E-01	-0.12	0.89	0.42	-0.29	7.75E-01
94	A*68:02-KIR2DL5-m2	4	4	0.37	1.45	0.50	0.73	4.63E-01	0.41	1.51	0.50	0.81	4.16E-01
95	R10-KIR2DL5-m1	28	16	-0.40	0.67	0.40	-1.00	3.16E-01	-0.33	0.72	0.40	-0.84	4.03E-01
96	A*01:01-KIR2DS1-m1	103	52	0.01	1.01	0.22	0.06	9.51E-01	0.21	1.24	0.22	0.96	3.35E-01
97	A*02:01-KIR2DS1-m1	165	88	0.07	1.07	0.20	0.34	7.32E-01	-0.07	0.93	0.20	-0.34	7.36E-01
98	A*02:05-KIR2DS1-m1	12	5	0.42	1.52	0.74	0.56	5.72E-01	0.43	1.54	0.74	0.59	5.58E-01
99	A*02:06-KIR2DS1-m2	6	1	-0.75	0.47	1.00	-0.75	4.56E-01	-0.55	0.58	1.00	-0.55	5.84E-01
100	A*03:01-KIR2DS1-m2	60	37	0.17	1.18	0.17	0.97	3.31E-01	0.22	1.24	0.17	1.25	2.10E-01
101	A*11:01-KIR2DS1-m1	23	13	-0.37	0.69	0.36	-1.01	3.14E-01	-0.28	0.76	0.36	-0.77	4.41E-01
102	A*23:01-KIR2DS1-m2	8	3	0.34	1.41	0.58	0.59	5.54E-01	0.25	1.28	0.58	0.42	6.73E-01
103	A*24:02-KIR2DS1-m2	40	32	0.33	1.39	0.18	1.78	7.45E-02	0.46	1.58	0.18	2.49	1.29E-02
104	A*25:01-KIR2DS1-m2	7	8	0.64	1.90	0.36	1.80	7.26E-02	0.39	1.48	0.36	1.09	2.75E-01
105	A*26:01-KIR2DS1-m2	18	10	-0.01	0.99	0.32	-0.03	9.76E-01	-0.31	0.73	0.32	-0.98	3.28E-01
106	A*29:02-KIR2DS1-m2	12	5	0.06	1.06	0.45	0.13	8.97E-01	0.53	1.70	0.45	1.18	2.39E-01
107	A*30:01-KIR2DS1-m2	4	0										
108	A*30:02-KIR2DS1-m1	8	9	0.32	1.37	0.51	0.61	5.39E-01	0.51	1.67	0.52	0.99	3.21E-01
109	A*31:01-KIR2DS1-m2	11	11	0.26	1.30	0.31	0.86	3.88E-01	-0.09	0.91	0.31	-0.29	7.71E-01
110	A*32:01-KIR2DS1-m1	13	7	1.07	2.93	0.70	1.54	1.24E-01	1.05	2.86	0.70	1.51	1.32E-01
111	A*33:01-KIR2DS1-m2	4	4	0.63	1.89	0.50	1.26	2.07E-01	0.56	1.75	0.50	1.11	2.66E-01
112	A*68:01-KIR2DS1-m1	19	6	-0.95	0.39	0.48	-1.98	4.72E-02	-0.75	0.47	0.48	-1.56	1.18E-01
113	A*68:02-KIR2DS1-m2	2	3	0.52	1.68	0.58	0.89	3.71E-01	0.21	1.24	0.58	0.37	7.14E-01

114	R10-KIR2DS1-m2	20	13	0.27	1.31	0.28	0.96	3.39E-01	0.14	1.15	0.28	0.48	6.31E-01
115	A*01:01-KIR2DS2-m2	128	64	0.03	1.03	0.14	0.22	8.25E-01	-0.03	0.97	0.14	-0.24	8.14E-01
116	A*02:01-KIR2DS2-m2	231	115	-0.08	0.92	0.11	-0.77	4.41E-01	-0.08	0.93	0.11	-0.68	4.94E-01
117	A*02:05-KIR2DS2-m2	17	4	-0.38	0.68	0.50	-0.76	4.47E-01	-0.52	0.60	0.50	-1.03	3.04E-01
118	A*02:06-KIR2DS2-m2	9	2	-0.32	0.72	0.71	-0.46	6.48E-01	-0.09	0.92	0.71	-0.12	9.03E-01
119	A*03:01-KIR2DS2-m1	95	48	-0.22	0.80	0.23	-0.94	3.50E-01	-0.19	0.83	0.23	-0.80	4.26E-01
120	A*11:01-KIR2DS2-m2	33	21	0.34	1.40	0.22	1.50	1.34E-01	0.14	1.15	0.22	0.63	5.29E-01
121	A*23:01-KIR2DS2-m2	14	6	0.02	1.02	0.41	0.05	9.57E-01	0.26	1.30	0.41	0.64	5.25E-01
122	A*24:02-KIR2DS2-m1	54	29	-0.30	0.74	0.27	-1.10	2.69E-01	-0.34	0.71	0.27	-1.25	2.12E-01
123	A*25:01-KIR2DS2-m2	16	10	0.17	1.19	0.32	0.54	5.91E-01	0.11	1.11	0.32	0.33	7.39E-01
124	A*26:01-KIR2DS2-m2	25	11	-0.09	0.91	0.31	-0.30	7.68E-01	-0.14	0.87	0.31	-0.47	6.41E-01
125	A*29:02-KIR2DS2-m2	20	12	0.46	1.59	0.29	1.58	1.13E-01	0.18	1.20	0.29	0.63	5.31E-01
126	A*30:01-KIR2DS2-m2	4	1	0.22	1.25	1.00	0.22	8.25E-01	1.31	3.72	1.00	1.31	1.91E-01
127	A*30:02-KIR2DS2-m1	9	10	0.40	1.49	0.53	0.76	4.46E-01	0.08	1.09	0.53	0.16	8.74E-01
128	A*31:01-KIR2DS2-m1	9	9	0.45	1.56	0.50	0.90	3.67E-01	0.62	1.87	0.50	1.25	2.10E-01
129	A*32:01-KIR2DS2-m1	18	6	0.45	1.57	0.65	0.69	4.87E-01	0.27	1.31	0.65	0.41	6.80E-01
130	A*33:01-KIR2DS2-m2	2	2	0.84	2.32	0.71	1.19	2.35E-01	0.79	2.19	0.71	1.11	2.68E-01
131	A*68:01-KIR2DS2-m1	23	10	-0.46	0.63	0.42	-1.08	2.79E-01	-0.23	0.80	0.42	-0.54	5.90E-01
132	A*68:02-KIR2DS2-m2	8	5	0.12	1.12	0.45	0.26	7.95E-01	0.08	1.09	0.45	0.18	8.56E-01
133	R10-KIR2DS2-m2	25	19	0.27	1.31	0.24	1.16	2.47E-01	0.11	1.12	0.24	0.47	6.40E-01
134	A*01:01-KIR2DS3-m1	67	34	0.03	1.03	0.24	0.13	8.96E-01	-0.10	0.90	0.24	-0.42	6.74E-01
135	A*02:01-KIR2DS3-m1	123	59	-0.30	0.74	0.22	-1.36	1.75E-01	-0.39	0.68	0.22	-1.75	7.99E-02
136	A*02:05-KIR2DS3-m1	7	2	0.34	1.41	0.82	0.42	6.76E-01	0.96	2.61	0.83	1.16	2.45E-01
137	A*02:06-KIR2DS3-m2	3	2	0.36	1.43	0.71	0.50	6.15E-01	0.88	2.42	0.71	1.24	2.14E-01
138	A*03:01-KIR2DS3-m2	42	30	0.24	1.27	0.19	1.27	2.03E-01	0.33	1.39	0.19	1.72	8.46E-02
139	A*11:01-KIR2DS3-m2	18	10	0.24	1.27	0.32	0.76	4.49E-01	0.32	1.38	0.32	1.01	3.14E-01
140	A*23:01-KIR2DS3-m2	8	4	0.25	1.29	0.50	0.50	6.17E-01	0.24	1.27	0.50	0.48	6.34E-01
141	A*24:02-KIR2DS3-m2	26	22	0.25	1.28	0.22	1.13	2.57E-01	0.33	1.39	0.22	1.51	1.30E-01
142	A*25:01-KIR2DS3-m1	9	5	0.05	1.05	0.54	0.09	9.31E-01	-0.37	0.69	0.54	-0.68	4.98E-01
143	A*26:01-KIR2DS3-m2	15	4	-0.48	0.62	0.50	-0.95	3.44E-01	-0.71	0.49	0.50	-1.41	1.59E-01
144	A*29:02-KIR2DS3-m1	11	6	-0.13	0.88	0.49	-0.26	7.97E-01	-0.30	0.74	0.49	-0.62	5.38E-01

145	A*30:01-KIR2DS3-m2	3	1	0.81	2.25	1.00	0.81	4.19E-01	1.95	7.03	1.01	1.94	5.25E-02
146	A*30:02-KIR2DS3-m1	2	6	0.53	1.70	0.53	1.00	3.16E-01	0.46	1.59	0.53	0.87	3.82E-01
147	A*31:01-KIR2DS3-m1	3	6	0.57	1.76	0.52	1.09	2.75E-01	0.83	2.29	0.52	1.59	1.11E-01
148	A*32:01-KIR2DS3-m1	9	5	0.82	2.26	0.64	1.27	2.04E-01	0.68	1.97	0.64	1.06	2.91E-01
149	A*33:01-KIR2DS3-m2	1	2	1.11	3.04	0.71	1.57	1.17E-01	0.91	2.49	0.71	1.29	1.98E-01
150	A*68:01-KIR2DS3-m2	14	6	-0.39	0.67	0.41	-0.96	3.38E-01	0.15	1.16	0.41	0.36	7.18E-01
151	A*68:02-KIR2DS3-m2	4	1	-0.56	0.57	1.00	-0.56	5.77E-01	0.11	1.11	1.00	0.11	9.16E-01
152	R10-KIR2DS3-m1	12	4	-1.07	0.34	0.55	-1.93	5.38E-02	-0.75	0.47	0.55	-1.36	1.73E-01
153	A*01:01-KIR2DS4-m2	245	120	-0.01	0.99	0.11	-0.13	8.98E-01	-0.06	0.94	0.11	-0.58	5.65E-01
154	A*02:01-KIR2DS4-m2	447	218	-0.17	0.84	0.10	-1.74	8.19E-02	-0.16	0.85	0.10	-1.60	1.09E-01
155	A*02:05-KIR2DS4-m2	29	7	-0.43	0.65	0.38	-1.12	2.65E-01	-0.54	0.58	0.38	-1.41	1.58E-01
156	A*02:06-KIR2DS4-m2	14	2	-0.91	0.40	0.71	-1.28	2.00E-01	-0.83	0.44	0.71	-1.17	2.43E-01
157	A*03:01-KIR2DS4-m1	167	88	-1.07	0.34	0.47	-2.29	2.22E-02	-1.01	0.36	0.47	-2.16	3.06E-02
158	A*11:01-KIR2DS4-m2	53	34	0.23	1.26	0.18	1.30	1.94E-01	0.11	1.11	0.18	0.59	5.57E-01
159	A*23:01-KIR2DS4-m2	20	10	0.16	1.18	0.32	0.51	6.07E-01	0.30	1.35	0.32	0.94	3.48E-01
160	A*24:02-KIR2DS4-m2	107	62	0.21	1.23	0.14	1.50	1.32E-01	0.38	1.47	0.14	2.77	5.66E-03
161	A*25:01-KIR2DS4-m2	26	17	0.14	1.15	0.25	0.58	5.64E-01	0.11	1.12	0.25	0.46	6.46E-01
162	A*26:01-KIR2DS4-m2	40	19	-0.14	0.87	0.24	-0.61	5.43E-01	-0.25	0.78	0.24	-1.05	2.93E-01
163	A*29:02-KIR2DS4-m2	40	21	0.33	1.40	0.22	1.48	1.38E-01	0.21	1.24	0.23	0.94	3.49E-01
164	A*30:01-KIR2DS4-m2	13	5	-0.12	0.89	0.45	-0.27	7.87E-01	0.03	1.03	0.45	0.07	9.46E-01
165	A*30:02-KIR2DS4-m2	25	16	0.14	1.15	0.26	0.56	5.79E-01	-0.05	0.96	0.26	-0.18	8.57E-01
166	A*31:01-KIR2DS4-m2	25	16	0.05	1.05	0.26	0.19	8.52E-01	-0.17	0.84	0.26	-0.68	4.95E-01
167	A*32:01-KIR2DS4-m2	36	10	-0.39	0.68	0.32	-1.22	2.21E-01	-0.10	0.90	0.32	-0.31	7.54E-01
168	A*33:01-KIR2DS4-m2	5	7	0.67	1.95	0.38	1.75	8.08E-02	0.56	1.75	0.38	1.46	1.45E-01
169	A*68:01-KIR2DS4-m1	37	23	0.90	2.46	0.78	1.16	2.46E-01	1.13	3.11	0.78	1.46	1.44E-01
170	A*68:02-KIR2DS4-m2	17	8	-0.04	0.96	0.36	-0.10	9.20E-01	0.13	1.14	0.36	0.37	7.12E-01
171	R10-KIR2DS4-m2	46	27	0.18	1.20	0.20	0.92	3.60E-01	0.10	1.10	0.20	0.50	6.20E-01
172	A*01:01-KIR2DS5-m2	83	46	0.17	1.18	0.16	1.07	2.87E-01	0.26	1.30	0.16	1.66	9.74E-02
173	A*02:01-KIR2DS5-m2	139	70	0.05	1.05	0.13	0.38	7.03E-01	0.04	1.04	0.13	0.29	7.70E-01
174	A*02:05-KIR2DS5-m2	11	4	-0.26	0.77	0.50	-0.51	6.11E-01	-0.41	0.67	0.50	-0.81	4.21E-01
175	A*02:06-KIR2DS5-m2	7	1	-0.84	0.43	1.00	-0.84	4.02E-01	-0.62	0.54	1.00	-0.62	5.33E-01

176	A*03:01-KIR2DS5-m2	50	30	0.19	1.21	0.19	1.02	3.09E-01	0.21	1.23	0.19	1.09	2.74E-01
177	A*11:01-KIR2DS5-m1	17	11	-0.26	0.77	0.38	-0.69	4.92E-01	-0.30	0.74	0.38	-0.80	4.25E-01
178	A*23:01-KIR2DS5-m1	7	2	-0.50	0.60	0.80	-0.63	5.27E-01	-0.54	0.58	0.80	-0.67	5.01E-01
179	A*24:02-KIR2DS5-m2	33	22	0.36	1.43	0.22	1.64	1.01E-01	0.48	1.62	0.22	2.20	2.77E-02
180	A*25:01-KIR2DS5-m1	5	7	0.83	2.29	0.50	1.67	9.41E-02	0.99	2.70	0.50	2.00	4.54E-02
181	A*26:01-KIR2DS5-m2	13	9	0.12	1.13	0.34	0.37	7.12E-01	-0.10	0.91	0.34	-0.29	7.69E-01
182	A*29:02-KIR2DS5-m2	11	5	0.08	1.09	0.45	0.19	8.51E-01	0.50	1.65	0.45	1.11	2.66E-01
183	A*30:01-KIR2DS5-m2	4	0										
184	A*30:02-KIR2DS5-m1	9	4	-0.55	0.58	0.59	-0.94	3.48E-01	-0.09	0.91	0.59	-0.16	8.75E-01
185	A*31:01-KIR2DS5-m2	11	9	0.25	1.28	0.34	0.74	4.58E-01	-0.16	0.85	0.34	-0.47	6.35E-01
186	A*32:01-KIR2DS5-m1	9	5	0.85	2.35	0.64	1.33	1.84E-01	0.90	2.45	0.64	1.40	1.62E-01
187	A*33:01-KIR2DS5-m1	4	2	-1.22	0.30	0.84	-1.44	1.49E-01	-0.92	0.40	0.84	-1.09	2.76E-01
188	A*68:01-KIR2DS5-m1	16	5	-0.96	0.38	0.51	-1.87	6.09E-02	-0.73	0.48	0.51	-1.43	1.53E-01
189	A*68:02-KIR2DS5-m2	1	3	0.56	1.75	0.58	0.96	3.35E-01	0.24	1.28	0.58	0.42	6.73E-01
190	R10-KIR2DS5-m2	19	13	0.33	1.39	0.28	1.17	2.41E-01	0.20	1.22	0.28	0.71	4.79E-01
191	A*01:01-KIR3DL1-m2	245	120	-0.01	0.99	0.11	-0.13	8.98E-01	-0.06	0.94	0.11	-0.58	5.65E-01
192	A*02:01-KIR3DL1-m2	447	218	-0.17	0.84	0.10	-1.74	8.19E-02	-0.16	0.85	0.10	-1.60	1.09E-01
193	A*02:05-KIR3DL1-m2	29	7	-0.43	0.65	0.38	-1.12	2.65E-01	-0.54	0.58	0.38	-1.41	1.58E-01
194	A*02:06-KIR3DL1-m2	14	2	-0.91	0.40	0.71	-1.28	2.00E-01	-0.83	0.44	0.71	-1.17	2.43E-01
195	A*03:01-KIR3DL1-m1	167	88	-1.05	0.35	0.47	-2.26	2.40E-02	-1.00	0.37	0.47	-2.16	3.11E-02
196	A*11:01-KIR3DL1-m2	53	34	0.23	1.26	0.18	1.30	1.94E-01	0.11	1.11	0.18	0.59	5.57E-01
197	A*23:01-KIR3DL1-m2	20	10	0.16	1.18	0.32	0.51	6.07E-01	0.30	1.35	0.32	0.94	3.48E-01
198	A*24:02-KIR3DL1-m2	108	62	0.20	1.23	0.14	1.48	1.40E-01	0.38	1.47	0.14	2.76	5.77E-03
199	A*25:01-KIR3DL1-m2	26	17	0.14	1.15	0.25	0.58	5.64E-01	0.11	1.12	0.25	0.46	6.46E-01
200	A*26:01-KIR3DL1-m2	40	19	-0.14	0.87	0.24	-0.61	5.43E-01	-0.25	0.78	0.24	-1.05	2.93E-01
201	A*29:02-KIR3DL1-m2	40	21	0.33	1.40	0.22	1.48	1.38E-01	0.21	1.24	0.23	0.94	3.49E-01
202	A*30:01-KIR3DL1-m2	13	5	-0.12	0.89	0.45	-0.27	7.87E-01	0.03	1.03	0.45	0.07	9.46E-01
203	A*30:02-KIR3DL1-m2	25	16	0.14	1.15	0.26	0.56	5.79E-01	-0.05	0.96	0.26	-0.18	8.57E-01
204	A*31:01-KIR3DL1-m2	25	16	0.05	1.05	0.26	0.19	8.52E-01	-0.17	0.84	0.26	-0.68	4.95E-01
205	A*32:01-KIR3DL1-m2	36	10	-0.39	0.68	0.32	-1.22	2.21E-01	-0.10	0.90	0.32	-0.31	7.54E-01
206	A*33:01-KIR3DL1-m2	5	7	0.67	1.95	0.38	1.75	8.08E-02	0.56	1.75	0.38	1.46	1.45E-01

207	A*68:01-KIR3DL1-m1	38	23	0.84	2.31	0.78	1.08	2.81E-01	1.12	3.07	0.78	1.44	1.49E-01
208	A*68:02-KIR3DL1-m2	17	8	-0.04	0.96	0.36	-0.10	9.20E-01	0.13	1.14	0.36	0.37	7.12E-01
209	R10-KIR3DL1-m2	46	27	0.18	1.20	0.20	0.92	3.60E-01	0.10	1.10	0.20	0.50	6.20E-01
210	A*01:01-KIR3DL2-m2	258	125	-0.02	0.98	0.11	-0.21	8.37E-01	-0.05	0.95	0.11	-0.51	6.10E-01
211	A*02:01-KIR3DL2-m2	470	228	-0.18	0.83	0.10	-1.82	6.90E-02	-0.16	0.85	0.10	-1.63	1.03E-01
212	A*02:05-KIR3DL2-m2	31	8	-0.41	0.67	0.36	-1.14	2.54E-01	-0.48	0.62	0.36	-1.34	1.80E-01
213	A*02:06-KIR3DL2-m2	15	2	-0.95	0.39	0.71	-1.34	1.80E-01	-0.83	0.44	0.71	-1.17	2.41E-01
214	A*03:01-KIR3DL2-m2	174	97	0.08	1.09	0.12	0.70	4.84E-01	0.07	1.08	0.12	0.64	5.24E-01
215	A*11:01-KIR3DL2-m2	58	36	0.23	1.26	0.18	1.31	1.90E-01	0.11	1.12	0.18	0.63	5.32E-01
216	A*23:01-KIR3DL2-m2	21	10	0.16	1.17	0.32	0.49	6.27E-01	0.28	1.32	0.32	0.88	3.81E-01
217	A*24:02-KIR3DL2-m2	111	64	0.21	1.23	0.14	1.52	1.28E-01	0.38	1.46	0.14	2.77	5.56E-03
218	A*25:01-KIR3DL2-m2	26	18	0.20	1.22	0.24	0.82	4.14E-01	0.12	1.13	0.24	0.50	6.17E-01
219	A*26:01-KIR3DL2-m2	41	19	-0.15	0.86	0.24	-0.65	5.14E-01	-0.25	0.78	0.24	-1.05	2.93E-01
220	A*29:02-KIR3DL2-m2	41	22	0.33	1.40	0.22	1.52	1.30E-01	0.24	1.27	0.22	1.08	2.80E-01
221	A*30:01-KIR3DL2-m2	13	5	-0.12	0.89	0.45	-0.27	7.87E-01	0.03	1.03	0.45	0.07	9.46E-01
222	A*30:02-KIR3DL2-m2	25	16	0.14	1.15	0.26	0.56	5.79E-01	-0.05	0.96	0.26	-0.18	8.57E-01
223	A*31:01-KIR3DL2-m2	25	17	0.10	1.10	0.25	0.39	6.99E-01	-0.13	0.88	0.25	-0.53	5.99E-01
224	A*32:01-KIR3DL2-m2	38	10	-0.43	0.65	0.32	-1.34	1.79E-01	-0.11	0.90	0.32	-0.34	7.35E-01
225	A*33:01-KIR3DL2-m2	5	7	0.67	1.95	0.38	1.75	8.08E-02	0.56	1.75	0.38	1.46	1.45E-01
226	A*68:01-KIR3DL2-m2	43	25	0.00	1.00	0.21	0.02	9.85E-01	0.12	1.12	0.21	0.56	5.77E-01
227	A*68:02-KIR3DL2-m2	18	8	-0.12	0.89	0.36	-0.33	7.42E-01	0.05	1.05	0.36	0.14	8.88E-01
228	R10-KIR3DL2-m2	48	28	0.19	1.21	0.20	0.98	3.28E-01	0.10	1.11	0.20	0.53	5.93E-01
229	A*01:01-KIR3DS1-m2	102	49	0.02	1.02	0.15	0.15	8.84E-01	0.07	1.08	0.15	0.49	6.24E-01
230	A*02:01-KIR3DS1-m1	161	84	0.11	1.12	0.21	0.55	5.79E-01	-0.11	0.90	0.21	-0.51	6.10E-01
231	A*02:05-KIR3DS1-m1	12	3	-0.18	0.84	0.74	-0.24	8.09E-01	0.62	1.86	0.74	0.84	4.02E-01
232	A*02:06-KIR3DS1-m2	6	1	-0.78	0.46	1.00	-0.78	4.35E-01	-0.53	0.59	1.00	-0.53	5.98E-01
233	A*03:01-KIR3DS1-m2	60	38	0.16	1.17	0.17	0.93	3.52E-01	0.19	1.21	0.17	1.12	2.64E-01
234	A*11:01-KIR3DS1-m1	22	12	-0.35	0.71	0.37	-0.94	3.47E-01	-0.17	0.85	0.37	-0.45	6.55E-01
235	A*23:01-KIR3DS1-m2	8	3	0.34	1.41	0.58	0.59	5.54E-01	0.25	1.28	0.58	0.42	6.73E-01
236	A*24:02-KIR3DS1-m2	41	31	0.29	1.34	0.19	1.56	1.19E-01	0.46	1.59	0.19	2.48	1.32E-02
237	A*25:01-KIR3DS1-m2	6	8	0.81	2.26	0.36	2.28	2.26E-02	0.41	1.51	0.36	1.15	2.50E-01

238	A*26:01-KIR3DS1-m2	16	9	0.02	1.02	0.34	0.07	9.45E-01	-0.16	0.85	0.34	-0.47	6.36E-01
239	A*29:02-KIR3DS1-m1	13	4	-0.74	0.48	0.56	-1.32	1.87E-01	-0.21	0.81	0.57	-0.38	7.07E-01
240	A*30:01-KIR3DS1-m2	4	0										
241	A*30:02-KIR3DS1-m1	8	7	0.15	1.17	0.51	0.30	7.67E-01	0.39	1.48	0.52	0.76	4.45E-01
242	A*31:01-KIR3DS1-m2	12	10	0.18	1.20	0.32	0.58	5.65E-01	-0.11	0.89	0.32	-0.35	7.23E-01
243	A*32:01-KIR3DS1-m1	12	7	1.25	3.50	0.70	1.80	7.24E-02	1.11	3.03	0.70	1.59	1.12E-01
244	A*33:01-KIR3DS1-m1	4	3	-0.61	0.54	0.77	-0.79	4.27E-01	-0.54	0.58	0.77	-0.70	4.86E-01
245	A*68:01-KIR3DS1-m1	21	6	-0.98	0.38	0.48	-2.04	4.13E-02	-0.76	0.47	0.48	-1.58	1.14E-01
246	A*68:02-KIR3DS1-m1	2	2	0.48	1.62	0.82	0.59	5.57E-01	-0.14	0.87	0.82	-0.17	8.66E-01
247	R10-KIR3DS1-m2	18	10	0.13	1.14	0.32	0.40	6.89E-01	0.15	1.17	0.32	0.48	6.29E-01
248	A*01:01-KIR2DP1-m2	252	121	-0.02	0.98	0.11	-0.18	8.57E-01	-0.06	0.94	0.11	-0.56	5.76E-01
249	A*02:01-KIR2DP1-m2	461	224	-0.18	0.83	0.10	-1.82	6.92E-02	-0.17	0.85	0.10	-1.65	9.89E-02
250	A*02:05-KIR2DP1-m2	29	8	-0.34	0.71	0.36	-0.94	3.46E-01	-0.43	0.65	0.36	-1.19	2.34E-01
251	A*02:06-KIR2DP1-m2	14	2	-0.93	0.40	0.71	-1.31	1.91E-01	-0.82	0.44	0.71	-1.16	2.48E-01
252	A*03:01-KIR2DP1-m2	171	95	0.07	1.07	0.12	0.60	5.50E-01	0.07	1.07	0.12	0.57	5.71E-01
253	A*11:01-KIR2DP1-m2	57	36	0.26	1.29	0.18	1.46	1.43E-01	0.13	1.14	0.18	0.74	4.56E-01
254	A*23:01-KIR2DP1-m2	20	10	0.18	1.19	0.32	0.55	5.80E-01	0.31	1.36	0.32	0.96	3.35E-01
255	A*24:02-KIR2DP1-m2	108	64	0.23	1.25	0.14	1.66	9.64E-02	0.38	1.47	0.14	2.80	5.18E-03
256	A*25:01-KIR2DP1-m2	26	17	0.17	1.19	0.25	0.70	4.84E-01	0.14	1.15	0.25	0.56	5.77E-01
257	A*26:01-KIR2DP1-m2	41	16	-0.26	0.77	0.26	-1.00	3.16E-01	-0.29	0.75	0.26	-1.13	2.58E-01
258	A*29:02-KIR2DP1-m2	37	20	0.34	1.41	0.23	1.48	1.39E-01	0.27	1.31	0.23	1.18	2.38E-01
259	A*30:01-KIR2DP1-m2	13	5	-0.12	0.89	0.45	-0.27	7.87E-01	0.03	1.03	0.45	0.07	9.46E-01
260	A*30:02-KIR2DP1-m2	25	15	0.11	1.11	0.26	0.40	6.87E-01	-0.07	0.93	0.26	-0.27	7.87E-01
261	A*31:01-KIR2DP1-m2	25	17	0.10	1.10	0.25	0.39	6.99E-01	-0.13	0.88	0.25	-0.53	5.99E-01
262	A*32:01-KIR2DP1-m2	37	10	-0.41	0.66	0.32	-1.28	1.99E-01	-0.10	0.91	0.32	-0.30	7.63E-01
263	A*33:01-KIR2DP1-m2	4	7	0.72	2.06	0.38	1.90	5.77E-02	0.58	1.79	0.38	1.53	1.26E-01
264	A*68:01-KIR2DP1-m2	42	25	0.01	1.01	0.21	0.06	9.54E-01	0.13	1.13	0.21	0.61	5.44E-01
265	A*68:02-KIR2DP1-m2	18	8	-0.12	0.89	0.36	-0.33	7.42E-01	0.05	1.05	0.36	0.14	8.88E-01
266	R10-KIR2DP1-m1	46	27	-0.65	0.52	1.07	-0.61	5.43E-01	-1.14	0.32	1.07	-1.06	2.87E-01
267	A*01:01-KIR3DP1-m2	258	125	-0.02	0.98	0.11	-0.21	8.37E-01	-0.05	0.95	0.11	-0.51	6.10E-01
268	A*02:01-KIR3DP1-m2	470	228	-0.18	0.83	0.10	-1.82	6.90E-02	-0.16	0.85	0.10	-1.63	1.03E-01

269	A*02:05-KIR3DP1-m2	31	8	-0.41	0.67	0.36	-1.14	2.54E-01	-0.48	0.62	0.36	-1.34	1.80E-01
270	A*02:06-KIR3DP1-m2	15	2	-0.95	0.39	0.71	-1.34	1.80E-01	-0.83	0.44	0.71	-1.17	2.41E-01
271	A*03:01-KIR3DP1-m2	174	97	0.08	1.09	0.12	0.70	4.84E-01	0.07	1.08	0.12	0.64	5.24E-01
272	A*11:01-KIR3DP1-m2	58	36	0.23	1.26	0.18	1.31	1.90E-01	0.11	1.12	0.18	0.63	5.32E-01
273	A*23:01-KIR3DP1-m2	21	10	0.16	1.17	0.32	0.49	6.27E-01	0.28	1.32	0.32	0.88	3.81E-01
274	A*24:02-KIR3DP1-m2	111	64	0.21	1.23	0.14	1.52	1.28E-01	0.38	1.46	0.14	2.77	5.56E-03
275	A*25:01-KIR3DP1-m2	26	18	0.20	1.22	0.24	0.82	4.14E-01	0.12	1.13	0.24	0.50	6.17E-01
276	A*26:01-KIR3DP1-m2	41	19	-0.15	0.86	0.24	-0.65	5.14E-01	-0.25	0.78	0.24	-1.05	2.93E-01
277	A*29:02-KIR3DP1-m2	41	22	0.33	1.40	0.22	1.52	1.30E-01	0.24	1.27	0.22	1.08	2.80E-01
278	A*30:01-KIR3DP1-m2	13	5	-0.12	0.89	0.45	-0.27	7.87E-01	0.03	1.03	0.45	0.07	9.46E-01
279	A*30:02-KIR3DP1-m2	25	16	0.14	1.15	0.26	0.56	5.79E-01	-0.05	0.96	0.26	-0.18	8.57E-01
280	A*31:01-KIR3DP1-m2	25	17	0.10	1.10	0.25	0.39	6.99E-01	-0.13	0.88	0.25	-0.53	5.99E-01
281	A*32:01-KIR3DP1-m2	38	10	-0.43	0.65	0.32	-1.34	1.79E-01	-0.11	0.90	0.32	-0.34	7.35E-01
282	A*33:01-KIR3DP1-m2	5	7	0.67	1.95	0.38	1.75	8.08E-02	0.56	1.75	0.38	1.46	1.45E-01
283	A*68:01-KIR3DP1-m2	43	25	0.00	1.00	0.21	0.02	9.85E-01	0.12	1.12	0.21	0.56	5.77E-01
284	A*68:02-KIR3DP1-m2	18	8	-0.12	0.89	0.36	-0.33	7.42E-01	0.05	1.05	0.36	0.14	8.88E-01
285	R10-KIR3DP1-m2	48	28	0.19	1.21	0.20	0.98	3.28E-01	0.10	1.11	0.20	0.53	5.93E-01

ESM Table 8. Interaction of HLA-B with KIR ligand numbers with the disease progression without (left panel) and with (right panel) adjustment for confounding effects: allelic frequency (left panel) or interactive frequency (right panel), estimated coefficient, hazard ratio, standard error, Z-score and p-value. P-values, if less than 0.05, are highlighted brown and light blue, if corresponding HR is less than 1 or greater than 1, respectively.

seq	B-KIR-model	Eff. Size		Unadjusted					Adjusted				
		n0	n1	coef	HR	SE	z	p	coef	HR	SE	z	p
1	B*07:02-KIR2DL1-m2	102	46	-0.13	0.87	0.16	-0.86	3.91E-01	-0.21	0.81	0.16	-1.35	1.77E-01
2	B*08:01-KIR2DL1-m1	257	135	-0.27	0.76	0.65	-0.41	6.78E-01	-0.61	0.54	0.65	-0.94	3.47E-01
3	B*13:02-KIR2DL1-m2	21	8	-0.23	0.79	0.36	-0.65	5.18E-01	0.46	1.58	0.36	1.27	2.03E-01
4	B*14:01-KIR2DL1-m2	11	5	-0.09	0.92	0.45	-0.20	8.45E-01	-0.32	0.73	0.45	-0.70	4.82E-01
5	B*14:02-KIR2DL1-m2	30	15	0.10	1.10	0.26	0.38	7.05E-01	0.00	1.00	0.26	0.01	9.94E-01
6	B*15:01-KIR2DL1-m2	199	100	-0.02	0.98	0.12	-0.18	8.56E-01	-0.15	0.86	0.12	-1.29	1.98E-01
7	B*15:18-KIR2DL1-m2	7	4	0.52	1.69	0.50	1.04	2.99E-01	0.89	2.44	0.50	1.77	7.64E-02
8	B*18:01-KIR2DL1-m2	82	62	0.39	1.48	0.14	2.84	4.54E-03	0.15	1.16	0.14	1.09	2.76E-01
9	B*27:05-KIR2DL1-m2	47	21	-0.18	0.83	0.22	-0.81	4.16E-01	-0.13	0.88	0.22	-0.58	5.63E-01
10	B*35:01-KIR2DL1-m2	47	30	-0.05	0.96	0.19	-0.24	8.10E-01	0.06	1.06	0.19	0.32	7.52E-01
11	B*35:03-KIR2DL1-m2	17	14	0.14	1.15	0.27	0.52	6.06E-01	0.07	1.07	0.27	0.25	8.06E-01
12	B*35:08-KIR2DL1-m2	6	5	0.27	1.31	0.45	0.60	5.47E-01	0.51	1.67	0.45	1.13	2.59E-01
13	B*37:01-KIR2DL1-m2	18	3	-0.94	0.39	0.58	-1.62	1.04E-01	-1.17	0.31	0.58	-2.02	4.36E-02
14	B*38:01-KIR2DL1-m1	22	17	-1.07	0.34	0.69	-1.55	1.22E-01	-0.25	0.78	0.69	-0.37	7.14E-01
15	B*39:01-KIR2DL1-m2	19	9	-0.05	0.95	0.34	-0.15	8.78E-01	0.50	1.65	0.34	1.48	1.39E-01
16	B*39:06-KIR2DL1-m2	19	13	0.11	1.12	0.28	0.40	6.91E-01	0.16	1.18	0.28	0.58	5.61E-01
17	B*40:01-KIR2DL1-m2	113	63	0.03	1.03	0.14	0.22	8.29E-01	0.09	1.10	0.14	0.68	4.98E-01
18	B*40:02-KIR2DL1-m2	24	4	-0.78	0.46	0.50	-1.56	1.20E-01	-0.08	0.92	0.51	-0.16	8.71E-01
19	B*41:01-KIR2DL1-m2	6	3	0.17	1.18	0.58	0.29	7.71E-01	0.30	1.35	0.58	0.52	6.05E-01
20	B*44:02-KIR2DL1-m2	130	61	-0.12	0.89	0.14	-0.84	4.03E-01	0.05	1.06	0.14	0.39	6.94E-01

21	B*44:03-KIR2DL1-m1	34	18	0.40	1.49	1.08	0.37	7.11E-01	0.82	2.27	1.08	0.76	4.50E-01
22	B*45:01-KIR2DL1-m2	12	3	-0.34	0.71	0.58	-0.59	5.56E-01	-0.65	0.52	0.58	-1.12	2.64E-01
23	B*49:01-KIR2DL1-m2	18	8	0.34	1.40	0.36	0.95	3.42E-01	0.22	1.24	0.36	0.61	5.44E-01
24	B*50:01-KIR2DL1-m2	19	10	0.24	1.26	0.32	0.73	4.63E-01	0.10	1.10	0.32	0.30	7.64E-01
25	B*51:01-KIR2DL1-m2	71	25	-0.34	0.71	0.21	-1.63	1.03E-01	-0.30	0.74	0.21	-1.47	1.42E-01
26	B*52:01-KIR2DL1-m2	9	2	-0.39	0.68	0.71	-0.54	5.86E-01	0.15	1.16	0.71	0.21	8.31E-01
27	B*53:01-KIR2DL1-m2	8	4	0.31	1.36	0.50	0.61	5.39E-01	0.27	1.30	0.50	0.53	5.99E-01
28	B*55:01-KIR2DL1-m2	27	13	0.04	1.05	0.28	0.16	8.75E-01	0.09	1.09	0.28	0.30	7.61E-01
29	B*56:01-KIR2DL1-m2	12	4	-0.04	0.96	0.50	-0.07	9.43E-01	0.76	2.14	0.50	1.51	1.32E-01
30	B*57:01-KIR2DL1-m2	15	6	-0.11	0.89	0.41	-0.28	7.83E-01	0.05	1.05	0.41	0.12	9.03E-01
31	B*58:01-KIR2DL1-m2	7	4	0.15	1.16	0.50	0.29	7.69E-01	0.16	1.18	0.50	0.32	7.48E-01
32	R10-KIR2DL1-m2	91	40	-0.14	0.87	0.17	-0.83	4.07E-01	-0.14	0.87	0.17	-0.82	4.12E-01
33	B*07:02-KIR2DL2-m2	45	23	-0.12	0.89	0.21	-0.56	5.72E-01	-0.24	0.78	0.22	-1.14	2.55E-01
34	B*08:01-KIR2DL2-m2	129	79	0.16	1.17	0.13	1.23	2.17E-01	0.07	1.07	0.13	0.53	5.97E-01
35	B*13:02-KIR2DL2-m1	9	2	-1.28	0.28	0.82	-1.56	1.19E-01	-1.17	0.31	0.82	-1.42	1.56E-01
36	B*14:01-KIR2DL2-m1	7	2	-1.78	0.17	0.92	-1.94	5.26E-02	-1.13	0.32	0.92	-1.23	2.19E-01
37	B*14:02-KIR2DL2-m1	17	4	-0.23	0.79	0.59	-0.39	6.94E-01	0.12	1.13	0.59	0.21	8.35E-01
38	B*15:01-KIR2DL2-m1	93	52	0.22	1.25	0.23	0.96	3.36E-01	0.28	1.32	0.23	1.20	2.31E-01
39	B*15:18-KIR2DL2-m1	7	1	-2.29	0.10	1.16	-1.97	4.87E-02	-1.55	0.21	1.16	-1.33	1.82E-01
40	B*18:01-KIR2DL2-m1	37	30	-0.01	0.99	0.27	-0.05	9.58E-01	-0.14	0.87	0.27	-0.52	6.05E-01
41	B*27:05-KIR2DL2-m1	28	9	-0.60	0.55	0.45	-1.34	1.81E-01	-0.59	0.55	0.45	-1.33	1.83E-01
42	B*35:01-KIR2DL2-m1	26	14	-0.37	0.69	0.38	-0.98	3.26E-01	-0.25	0.78	0.38	-0.65	5.19E-01
43	B*35:03-KIR2DL2-m2	8	10	0.41	1.51	0.32	1.29	1.97E-01	0.12	1.13	0.32	0.36	7.15E-01
44	B*35:08-KIR2DL2-m2	1	3	0.56	1.74	0.58	0.96	3.37E-01	0.56	1.76	0.58	0.97	3.30E-01
45	B*37:01-KIR2DL2-m2	11	1	-1.58	0.21	1.00	-1.58	1.14E-01	-1.62	0.20	1.00	-1.62	1.05E-01
46	B*38:01-KIR2DL2-m1	14	12	0.22	1.25	0.45	0.49	6.24E-01	0.24	1.27	0.46	0.52	6.04E-01
47	B*39:01-KIR2DL2-m2	10	6	0.34	1.40	0.41	0.82	4.10E-01	0.85	2.35	0.41	2.06	3.95E-02
48	B*39:06-KIR2DL2-m1	12	6	-0.53	0.59	0.55	-0.97	3.32E-01	-0.34	0.71	0.55	-0.61	5.41E-01
49	B*40:01-KIR2DL2-m2	56	27	-0.06	0.94	0.20	-0.29	7.73E-01	0.05	1.06	0.20	0.27	7.86E-01
50	B*40:02-KIR2DL2-m1	11	1	-0.79	0.45	1.16	-0.68	4.96E-01	-0.57	0.57	1.16	-0.49	6.23E-01
51	B*41:01-KIR2DL2-m1	6	1	-1.85	0.16	1.23	-1.50	1.33E-01	-1.19	0.30	1.23	-0.97	3.32E-01

52	B*44:02-KIR2DL2-m2	62	34	0.03	1.03	0.18	0.16	8.77E-01	0.07	1.08	0.18	0.42	6.78E-01
53	B*44:03-KIR2DL2-m1	18	12	0.86	2.37	0.49	1.77	7.69E-02	0.74	2.09	0.49	1.51	1.30E-01
54	B*45:01-KIR2DL2-m1	7	2	-0.38	0.68	1.01	-0.38	7.03E-01	-1.89	0.15	1.01	-1.88	6.03E-02
55	B*49:01-KIR2DL2-m2	8	3	0.72	2.06	0.58	1.24	2.15E-01	0.33	1.39	0.58	0.57	5.70E-01
56	B*50:01-KIR2DL2-m2	13	6	0.31	1.36	0.41	0.75	4.54E-01	0.05	1.05	0.41	0.11	9.09E-01
57	B*51:01-KIR2DL2-m2	35	13	-0.23	0.79	0.28	-0.83	4.07E-01	-0.22	0.80	0.28	-0.78	4.35E-01
58	B*52:01-KIR2DL2-m2	8	2	-0.33	0.72	0.71	-0.46	6.44E-01	0.42	1.53	0.71	0.59	5.53E-01
59	B*53:01-KIR2DL2-m2	5	3	0.34	1.41	0.58	0.59	5.54E-01	0.54	1.72	0.58	0.93	3.53E-01
60	B*55:01-KIR2DL2-m1	16	5	-0.81	0.44	0.58	-1.40	1.60E-01	-0.42	0.66	0.58	-0.73	4.68E-01
61	B*56:01-KIR2DL2-m2	5	1	-0.15	0.86	1.00	-0.15	8.82E-01	1.36	3.89	1.01	1.35	1.77E-01
62	B*57:01-KIR2DL2-m1	7	2	-0.74	0.48	0.87	-0.85	3.94E-01	-0.77	0.46	0.87	-0.88	3.77E-01
63	B*58:01-KIR2DL2-m1	6	2	-2.09	0.12	1.01	-2.07	3.81E-02	-3.76	0.02	1.02	-3.69	2.25E-04
64	R10-KIR2DL2-m2	47	20	-0.03	0.97	0.23	-0.15	8.81E-01	-0.14	0.87	0.23	-0.59	5.56E-01
65	B*07:02-KIR2DL3-m1	96	41	-0.53	0.59	0.51	-1.05	2.94E-01	-1.36	0.26	0.51	-2.66	7.76E-03
66	B*08:01-KIR2DL3-m1	243	128	-0.06	0.94	0.36	-0.17	8.65E-01	0.13	1.14	0.36	0.36	7.22E-01
67	B*13:02-KIR2DL3-m2	20	7	-0.33	0.72	0.38	-0.87	3.83E-01	0.35	1.42	0.38	0.91	3.65E-01
68	B*14:01-KIR2DL3-m2	11	5	-0.09	0.92	0.45	-0.20	8.45E-01	-0.32	0.73	0.45	-0.70	4.82E-01
69	B*14:02-KIR2DL3-m1	28	13	-0.17	0.85	0.78	-0.21	8.30E-01	-0.29	0.75	0.78	-0.37	7.14E-01
70	B*15:01-KIR2DL3-m1	193	91	-0.47	0.63	0.39	-1.22	2.24E-01	-0.69	0.50	0.39	-1.78	7.56E-02
71	B*15:18-KIR2DL3-m2	6	4	0.74	2.11	0.50	1.48	1.39E-01	1.03	2.80	0.50	2.05	4.07E-02
72	B*18:01-KIR2DL3-m1	77	56	-0.30	0.74	0.42	-0.71	4.80E-01	-0.59	0.56	0.42	-1.39	1.65E-01
73	B*27:05-KIR2DL3-m1	46	19	-1.15	0.32	0.65	-1.78	7.53E-02	-0.94	0.39	0.65	-1.46	1.45E-01
74	B*35:01-KIR2DL3-m2	45	30	-0.02	0.98	0.19	-0.09	9.31E-01	0.07	1.07	0.19	0.36	7.19E-01
75	B*35:03-KIR2DL3-m1	14	12	0.14	1.15	0.78	0.18	8.57E-01	0.59	1.80	0.79	0.75	4.53E-01
76	B*35:08-KIR2DL3-m2	6	5	0.27	1.31	0.45	0.60	5.47E-01	0.51	1.67	0.45	1.13	2.59E-01
77	B*37:01-KIR2DL3-m2	16	3	-0.76	0.47	0.58	-1.32	1.88E-01	-0.95	0.39	0.58	-1.63	1.03E-01
78	B*38:01-KIR2DL3-m1	18	15	-0.07	0.93	0.52	-0.13	8.93E-01	0.40	1.48	0.52	0.76	4.46E-01
79	B*39:01-KIR2DL3-m2	17	8	-0.07	0.94	0.36	-0.18	8.54E-01	0.48	1.62	0.36	1.33	1.83E-01
80	B*39:06-KIR2DL3-m1	18	11	-0.21	0.81	0.67	-0.31	7.57E-01	-0.54	0.58	0.68	-0.80	4.24E-01
81	B*40:01-KIR2DL3-m2	107	59	0.03	1.03	0.14	0.23	8.20E-01	0.10	1.10	0.14	0.70	4.82E-01
82	B*40:02-KIR2DL3-m2	21	4	-0.66	0.52	0.50	-1.31	1.90E-01	0.01	1.01	0.51	0.01	9.91E-01

83	B*41:01-KIR2DL3-m1	5	2	0.69	1.99	1.24	0.56	5.79E-01	0.06	1.06	1.24	0.05	9.61E-01
84	B*44:02-KIR2DL3-m1	121	59	0.92	2.51	0.62	1.49	1.36E-01	1.04	2.82	0.62	1.68	9.37E-02
85	B*44:03-KIR2DL3-m1	29	18	1.15	3.16	1.04	1.10	2.69E-01	1.29	3.64	1.04	1.24	2.15E-01
86	B*45:01-KIR2DL3-m2	12	3	-0.34	0.71	0.58	-0.59	5.56E-01	-0.65	0.52	0.58	-1.12	2.64E-01
87	B*49:01-KIR2DL3-m2	18	8	0.34	1.40	0.36	0.95	3.42E-01	0.22	1.24	0.36	0.61	5.44E-01
88	B*50:01-KIR2DL3-m1	18	9	0.23	1.25	0.80	0.28	7.79E-01	0.11	1.12	0.80	0.14	8.87E-01
89	B*51:01-KIR2DL3-m2	68	23	-0.36	0.70	0.21	-1.67	9.42E-02	-0.31	0.73	0.21	-1.44	1.49E-01
90	B*52:01-KIR2DL3-m2	9	1	-1.08	0.34	1.00	-1.08	2.82E-01	-0.51	0.60	1.00	-0.51	6.11E-01
91	B*53:01-KIR2DL3-m2	7	4	0.34	1.40	0.50	0.67	5.05E-01	0.28	1.32	0.50	0.55	5.79E-01
92	B*55:01-KIR2DL3-m2	24	13	0.14	1.15	0.28	0.49	6.21E-01	0.24	1.28	0.28	0.86	3.90E-01
93	B*56:01-KIR2DL3-m2	11	4	0.11	1.12	0.50	0.22	8.23E-01	0.79	2.21	0.50	1.57	1.16E-01
94	B*57:01-KIR2DL3-m2	14	6	-0.11	0.90	0.41	-0.26	7.91E-01	0.05	1.05	0.41	0.12	9.03E-01
95	B*58:01-KIR2DL3-m2	6	4	0.28	1.32	0.50	0.55	5.84E-01	0.20	1.22	0.51	0.39	6.94E-01
96	R10-KIR2DL3-m2	84	36	-0.16	0.85	0.17	-0.92	3.58E-01	-0.18	0.84	0.17	-1.03	3.03E-01
97	B*07:02-KIR2DL4-m2	103	46	-0.14	0.87	0.16	-0.87	3.85E-01	-0.21	0.81	0.16	-1.35	1.77E-01
98	B*08:01-KIR2DL4-m2	262	141	0.09	1.09	0.10	0.85	3.98E-01	0.05	1.05	0.10	0.46	6.43E-01
99	B*13:02-KIR2DL4-m2	21	8	-0.23	0.79	0.36	-0.65	5.18E-01	0.46	1.58	0.36	1.27	2.03E-01
100	B*14:01-KIR2DL4-m2	11	5	-0.09	0.92	0.45	-0.20	8.45E-01	-0.32	0.73	0.45	-0.70	4.82E-01
101	B*14:02-KIR2DL4-m2	34	15	0.06	1.06	0.26	0.21	8.31E-01	-0.04	0.96	0.26	-0.15	8.79E-01
102	B*15:01-KIR2DL4-m2	203	101	-0.02	0.98	0.11	-0.21	8.38E-01	-0.15	0.86	0.11	-1.28	2.02E-01
103	B*15:18-KIR2DL4-m2	8	4	0.45	1.57	0.50	0.89	3.73E-01	0.77	2.16	0.50	1.53	1.26E-01
104	B*18:01-KIR2DL4-m2	83	64	0.40	1.49	0.14	2.93	3.35E-03	0.16	1.18	0.14	1.20	2.30E-01
105	B*27:05-KIR2DL4-m2	47	22	-0.15	0.86	0.22	-0.68	4.99E-01	-0.10	0.91	0.22	-0.45	6.50E-01
106	B*35:01-KIR2DL4-m2	48	30	-0.06	0.94	0.19	-0.30	7.65E-01	0.04	1.04	0.19	0.22	8.25E-01
107	B*35:03-KIR2DL4-m2	17	14	0.14	1.15	0.27	0.52	6.06E-01	0.07	1.07	0.27	0.25	8.06E-01
108	B*35:08-KIR2DL4-m2	6	5	0.27	1.31	0.45	0.60	5.47E-01	0.51	1.67	0.45	1.13	2.59E-01
109	B*37:01-KIR2DL4-m2	19	3	-1.01	0.36	0.58	-1.74	8.13E-02	-1.25	0.29	0.58	-2.16	3.11E-02
110	B*38:01-KIR2DL4-m2	22	21	0.22	1.24	0.22	0.97	3.31E-01	0.00	1.00	0.23	0.00	9.98E-01
111	B*39:01-KIR2DL4-m2	19	9	-0.05	0.95	0.34	-0.15	8.78E-01	0.50	1.65	0.34	1.48	1.39E-01
112	B*39:06-KIR2DL4-m2	20	14	0.12	1.12	0.27	0.43	6.66E-01	0.22	1.25	0.27	0.81	4.20E-01
113	B*40:01-KIR2DL4-m2	114	63	0.02	1.02	0.14	0.12	9.05E-01	0.09	1.10	0.14	0.67	5.02E-01

114	B*40:02-KIR2DL4-m2	24	4	-0.78	0.46	0.50	-1.56	1.20E-01	-0.08	0.92	0.51	-0.16	8.71E-01
115	B*41:01-KIR2DL4-m2	8	3	-0.10	0.90	0.58	-0.18	8.58E-01	0.04	1.04	0.58	0.07	9.46E-01
116	B*44:02-KIR2DL4-m2	134	62	-0.12	0.89	0.14	-0.88	3.78E-01	0.06	1.06	0.14	0.42	6.77E-01
117	B*44:03-KIR2DL4-m2	37	19	0.03	1.03	0.24	0.13	8.98E-01	0.10	1.10	0.24	0.41	6.80E-01
118	B*45:01-KIR2DL4-m2	13	4	-0.14	0.87	0.50	-0.27	7.88E-01	-0.45	0.64	0.50	-0.90	3.68E-01
119	B*49:01-KIR2DL4-m2	19	8	0.32	1.38	0.36	0.89	3.73E-01	0.21	1.23	0.36	0.58	5.59E-01
120	B*50:01-KIR2DL4-m2	20	11	0.24	1.27	0.31	0.78	4.34E-01	0.03	1.03	0.31	0.11	9.12E-01
121	B*51:01-KIR2DL4-m2	75	25	-0.35	0.70	0.21	-1.72	8.59E-02	-0.31	0.73	0.21	-1.50	1.34E-01
122	B*52:01-KIR2DL4-m2	10	2	-0.50	0.61	0.71	-0.70	4.82E-01	0.11	1.12	0.71	0.16	8.73E-01
123	B*53:01-KIR2DL4-m2	8	4	0.31	1.36	0.50	0.61	5.39E-01	0.27	1.30	0.50	0.53	5.99E-01
124	B*55:01-KIR2DL4-m2	28	13	-0.02	0.98	0.28	-0.07	9.41E-01	0.03	1.03	0.28	0.11	9.16E-01
125	B*56:01-KIR2DL4-m2	12	4	-0.04	0.96	0.50	-0.07	9.43E-01	0.76	2.14	0.50	1.51	1.32E-01
126	B*57:01-KIR2DL4-m2	15	6	-0.11	0.89	0.41	-0.28	7.83E-01	0.05	1.05	0.41	0.12	9.03E-01
127	B*58:01-KIR2DL4-m2	7	4	0.15	1.16	0.50	0.29	7.69E-01	0.16	1.18	0.50	0.32	7.48E-01
128	R10-KIR2DL4-m2	92	40	-0.14	0.87	0.17	-0.84	4.02E-01	-0.14	0.87	0.17	-0.83	4.09E-01
129	B*07:02-KIR2DL5-m2	41	22	0.03	1.03	0.22	0.15	8.83E-01	-0.12	0.89	0.22	-0.53	5.96E-01
130	B*08:01-KIR2DL5-m2	125	76	0.21	1.23	0.13	1.63	1.02E-01	0.15	1.17	0.13	1.21	2.28E-01
131	B*13:02-KIR2DL5-m1	10	4	-0.39	0.68	0.71	-0.55	5.83E-01	-0.49	0.61	0.71	-0.69	4.91E-01
132	B*14:01-KIR2DL5-m1	6	2	-1.24	0.29	0.92	-1.35	1.77E-01	-1.00	0.37	0.92	-1.08	2.78E-01
133	B*14:02-KIR2DL5-m1	14	5	0.24	1.27	0.56	0.43	6.66E-01	0.28	1.33	0.56	0.51	6.11E-01
134	B*15:01-KIR2DL5-m2	85	53	0.18	1.20	0.15	1.25	2.10E-01	-0.06	0.95	0.15	-0.38	7.07E-01
135	B*15:18-KIR2DL5-m1	5	1	-1.68	0.19	1.16	-1.45	1.48E-01	-0.80	0.45	1.16	-0.69	4.89E-01
136	B*18:01-KIR2DL5-m2	38	31	0.41	1.51	0.19	2.20	2.80E-02	0.18	1.20	0.19	0.97	3.34E-01
137	B*27:05-KIR2DL5-m2	22	10	-0.18	0.83	0.32	-0.57	5.69E-01	-0.15	0.86	0.32	-0.47	6.41E-01
138	B*35:01-KIR2DL5-m1	23	18	0.12	1.13	0.39	0.31	7.57E-01	-0.12	0.88	0.39	-0.32	7.51E-01
139	B*35:03-KIR2DL5-m2	9	8	0.24	1.27	0.36	0.66	5.08E-01	0.23	1.26	0.36	0.65	5.13E-01
140	B*35:08-KIR2DL5-m1	4	1	-1.38	0.25	1.12	-1.23	2.19E-01	-0.60	0.55	1.12	-0.53	5.96E-01
141	B*37:01-KIR2DL5-m2	11	1	-1.53	0.22	1.00	-1.53	1.27E-01	-1.44	0.24	1.00	-1.44	1.51E-01
142	B*38:01-KIR2DL5-m1	12	9	-0.42	0.65	0.45	-0.94	3.50E-01	-0.58	0.56	0.45	-1.27	2.04E-01
143	B*39:01-KIR2DL5-m2	10	6	0.29	1.34	0.41	0.71	4.76E-01	0.72	2.06	0.41	1.75	8.04E-02
144	B*39:06-KIR2DL5-m1	10	7	-0.49	0.61	0.54	-0.90	3.69E-01	-0.45	0.64	0.55	-0.81	4.15E-01

145	B*40:01-KIR2DL5-m1	63	28	-0.29	0.75	0.28	-1.05	2.93E-01	-0.15	0.86	0.28	-0.56	5.77E-01
146	B*40:02-KIR2DL5-m1	9	3	0.99	2.68	1.16	0.85	3.94E-01	0.93	2.53	1.16	0.80	4.24E-01
147	B*41:01-KIR2DL5-m1	2	2	1.28	3.60	1.23	1.04	2.98E-01	1.28	3.61	1.23	1.04	2.97E-01
148	B*44:02-KIR2DL5-m1	70	28	-0.56	0.57	0.28	-2.00	4.57E-02	-0.26	0.77	0.28	-0.92	3.57E-01
149	B*44:03-KIR2DL5-m1	20	8	-0.55	0.58	0.48	-1.15	2.49E-01	-0.22	0.80	0.48	-0.46	6.42E-01
150	B*45:01-KIR2DL5-m2	6	1	-0.78	0.46	1.00	-0.78	4.35E-01	-1.21	0.30	1.00	-1.21	2.26E-01
151	B*49:01-KIR2DL5-m1	10	4	-0.25	0.78	0.71	-0.35	7.28E-01	-0.44	0.65	0.72	-0.61	5.42E-01
152	B*50:01-KIR2DL5-m2	9	6	0.58	1.78	0.41	1.40	1.61E-01	0.19	1.22	0.41	0.47	6.38E-01
153	B*51:01-KIR2DL5-m1	33	16	0.74	2.09	0.43	1.72	8.50E-02	0.68	1.97	0.43	1.57	1.15E-01
154	B*52:01-KIR2DL5-m2	7	2	-0.36	0.70	0.71	-0.50	6.15E-01	0.14	1.15	0.71	0.20	8.44E-01
155	B*53:01-KIR2DL5-m2	4	4	0.55	1.73	0.50	1.09	2.77E-01	0.66	1.93	0.50	1.31	1.91E-01
156	B*55:01-KIR2DL5-m1	17	5	-0.76	0.47	0.58	-1.31	1.90E-01	-0.95	0.39	0.58	-1.64	1.00E-01
157	B*56:01-KIR2DL5-m1	7	1	-1.11	0.33	1.16	-0.96	3.37E-01	-1.38	0.25	1.16	-1.19	2.36E-01
158	B*57:01-KIR2DL5-m1	8	2	-0.27	0.76	0.87	-0.31	7.53E-01	-0.37	0.69	0.87	-0.43	6.69E-01
159	B*58:01-KIR2DL5-m1	5	1	-0.90	0.41	1.16	-0.77	4.39E-01	-2.36	0.09	1.17	-2.02	4.33E-02
160	R10-KIR2DL5-m1	52	27	0.23	1.25	0.35	0.64	5.23E-01	0.44	1.55	0.35	1.24	2.17E-01
161	B*07:02-KIR2DS1-m1	29	17	0.39	1.47	0.32	1.19	2.33E-01	0.15	1.17	0.32	0.47	6.37E-01
162	B*08:01-KIR2DS1-m2	103	61	0.15	1.17	0.14	1.10	2.72E-01	0.11	1.12	0.14	0.81	4.17E-01
163	B*13:02-KIR2DS1-m2	7	3	-0.22	0.80	0.58	-0.38	7.02E-01	0.45	1.56	0.58	0.77	4.43E-01
164	B*14:01-KIR2DS1-m1	3	2	0.14	1.15	0.92	0.15	8.78E-01	0.97	2.64	0.92	1.05	2.93E-01
165	B*14:02-KIR2DS1-m1	10	5	0.44	1.56	0.56	0.79	4.27E-01	0.36	1.43	0.56	0.64	5.19E-01
166	B*15:01-KIR2DS1-m2	71	45	0.25	1.28	0.16	1.58	1.14E-01	-0.03	0.97	0.16	-0.20	8.44E-01
167	B*15:18-KIR2DS1-m2	3	1	-0.06	0.94	1.00	-0.06	9.51E-01	0.70	2.01	1.00	0.70	4.87E-01
168	B*18:01-KIR2DS1-m2	29	24	0.39	1.48	0.21	1.86	6.35E-02	0.16	1.17	0.21	0.74	4.62E-01
169	B*27:05-KIR2DS1-m1	15	9	0.21	1.23	0.45	0.47	6.37E-01	0.19	1.21	0.45	0.42	6.74E-01
170	B*35:01-KIR2DS1-m1	18	16	0.24	1.27	0.38	0.63	5.27E-01	-0.20	0.82	0.38	-0.53	5.95E-01
171	B*35:03-KIR2DS1-m1	8	5	-0.64	0.53	0.57	-1.12	2.63E-01	-0.35	0.70	0.57	-0.61	5.39E-01
172	B*35:08-KIR2DS1-m1	4	1	-1.44	0.24	1.12	-1.28	2.00E-01	-0.64	0.53	1.12	-0.57	5.71E-01
173	B*37:01-KIR2DS1-m2	9	1	-1.22	0.30	1.00	-1.22	2.24E-01	-0.85	0.43	1.00	-0.85	3.97E-01
174	B*38:01-KIR2DS1-m1	9	8	-0.37	0.69	0.46	-0.80	4.22E-01	-0.66	0.52	0.46	-1.42	1.55E-01
175	B*39:01-KIR2DS1-m2	4	4	1.05	2.86	0.50	2.09	3.68E-02	1.37	3.92	0.50	2.71	6.75E-03

176	B*39:06-KIR2DS1-m2	7	6	0.11	1.12	0.41	0.27	7.86E-01	0.42	1.52	0.41	1.01	3.13E-01
177	B*40:01-KIR2DS1-m2	43	23	0.09	1.10	0.21	0.43	6.70E-01	0.18	1.19	0.21	0.82	4.13E-01
178	B*40:02-KIR2DS1-m1	5	2	1.42	4.13	1.01	1.41	1.58E-01	1.09	2.97	1.01	1.08	2.79E-01
179	B*41:01-KIR2DS1-m2	1	1	1.02	2.79	1.00	1.02	3.06E-01	0.28	1.32	1.00	0.28	7.82E-01
180	B*44:02-KIR2DS1-m2	53	24	-0.19	0.83	0.21	-0.90	3.70E-01	0.21	1.24	0.21	1.00	3.16E-01
181	B*44:03-KIR2DS1-m1	16	6	-0.71	0.49	0.50	-1.40	1.62E-01	-0.22	0.81	0.51	-0.42	6.72E-01
182	B*45:01-KIR2DS1-m2	5	0										
183	B*49:01-KIR2DS1-m2	6	4	0.39	1.48	0.50	0.77	4.39E-01	0.34	1.40	0.50	0.67	5.05E-01
184	B*50:01-KIR2DS1-m2	10	6	0.54	1.71	0.41	1.30	1.94E-01	0.18	1.20	0.41	0.44	6.57E-01
185	B*51:01-KIR2DS1-m2	28	10	-0.19	0.83	0.32	-0.60	5.51E-01	-0.14	0.87	0.32	-0.45	6.54E-01
186	B*52:01-KIR2DS1-m1	5	1	-0.17	0.84	1.42	-0.12	9.05E-01	-0.18	0.83	1.42	-0.13	8.98E-01
187	B*53:01-KIR2DS1-m2	2	3	1.06	2.89	0.58	1.83	6.70E-02	0.66	1.93	0.58	1.13	2.58E-01
188	B*55:01-KIR2DS1-m1	14	3	-1.18	0.31	0.67	-1.77	7.63E-02	-1.34	0.26	0.67	-2.01	4.41E-02
189	B*56:01-KIR2DS1-m1	7	1	-1.41	0.25	1.16	-1.21	2.26E-01	-1.62	0.20	1.16	-1.39	1.63E-01
190	B*57:01-KIR2DS1-m1	7	2	-0.29	0.75	0.87	-0.34	7.36E-01	-0.42	0.66	0.87	-0.48	6.33E-01
191	B*58:01-KIR2DS1-m1	3	1	0.08	1.08	1.16	0.07	9.47E-01	-1.21	0.30	1.17	-1.03	3.01E-01
192	R10-KIR2DS1-m2	37	20	-0.15	0.86	0.23	-0.64	5.21E-01	-0.07	0.93	0.23	-0.31	7.53E-01
193	B*07:02-KIR2DS2-m2	44	23	-0.10	0.90	0.21	-0.47	6.36E-01	-0.22	0.80	0.22	-1.04	2.96E-01
194	B*08:01-KIR2DS2-m2	132	82	0.18	1.20	0.12	1.47	1.41E-01	0.09	1.10	0.12	0.77	4.44E-01
195	B*13:02-KIR2DS2-m1	9	2	-1.31	0.27	0.82	-1.59	1.13E-01	-1.20	0.30	0.82	-1.46	1.45E-01
196	B*14:01-KIR2DS2-m1	7	2	-1.81	0.16	0.92	-1.96	4.97E-02	-1.16	0.31	0.92	-1.26	2.08E-01
197	B*14:02-KIR2DS2-m1	17	4	-0.26	0.77	0.59	-0.43	6.66E-01	0.09	1.10	0.59	0.16	8.75E-01
198	B*15:01-KIR2DS2-m1	94	52	0.18	1.20	0.23	0.78	4.36E-01	0.23	1.26	0.23	1.00	3.18E-01
199	B*15:18-KIR2DS2-m1	7	1	-2.31	0.10	1.16	-1.99	4.66E-02	-1.58	0.21	1.16	-1.36	1.74E-01
200	B*18:01-KIR2DS2-m2	37	32	0.44	1.56	0.18	2.41	1.59E-02	0.14	1.15	0.18	0.74	4.59E-01
201	B*27:05-KIR2DS2-m1	29	9	-0.73	0.48	0.45	-1.64	1.02E-01	-0.65	0.52	0.45	-1.46	1.44E-01
202	B*35:01-KIR2DS2-m1	26	15	-0.28	0.75	0.38	-0.74	4.58E-01	-0.17	0.85	0.38	-0.44	6.60E-01
203	B*35:03-KIR2DS2-m2	8	10	0.41	1.51	0.32	1.29	1.97E-01	0.12	1.13	0.32	0.36	7.15E-01
204	B*35:08-KIR2DS2-m2	1	3	0.56	1.74	0.58	0.96	3.37E-01	0.56	1.76	0.58	0.97	3.30E-01
205	B*37:01-KIR2DS2-m2	11	1	-1.58	0.21	1.00	-1.58	1.14E-01	-1.62	0.20	1.00	-1.62	1.05E-01
206	B*38:01-KIR2DS2-m1	15	12	0.12	1.13	0.45	0.26	7.92E-01	0.17	1.18	0.46	0.37	7.11E-01

207	B*39:01-KIR2DS2-m2	10	6	0.34	1.40	0.41	0.82	4.10E-01	0.85	2.35	0.41	2.06	3.95E-02
208	B*39:06-KIR2DS2-m1	12	6	-0.56	0.57	0.55	-1.01	3.11E-01	-0.37	0.69	0.55	-0.67	5.05E-01
209	B*40:01-KIR2DS2-m2	56	28	-0.03	0.97	0.20	-0.13	8.93E-01	0.09	1.09	0.20	0.45	6.55E-01
210	B*40:02-KIR2DS2-m1	10	1	-0.71	0.49	1.16	-0.61	5.39E-01	-0.46	0.63	1.16	-0.40	6.90E-01
211	B*41:01-KIR2DS2-m1	6	1	-1.87	0.15	1.23	-1.52	1.28E-01	-1.22	0.29	1.23	-0.99	3.20E-01
212	B*44:02-KIR2DS2-m2	63	34	0.01	1.01	0.18	0.07	9.47E-01	0.07	1.07	0.18	0.40	6.89E-01
213	B*44:03-KIR2DS2-m1	19	12	0.71	2.04	0.49	1.46	1.43E-01	0.59	1.81	0.49	1.21	2.25E-01
214	B*45:01-KIR2DS2-m1	7	2	-0.41	0.67	1.01	-0.40	6.86E-01	-1.92	0.15	1.01	-1.91	5.64E-02
215	B*49:01-KIR2DS2-m2	8	3	0.72	2.06	0.58	1.24	2.15E-01	0.33	1.39	0.58	0.57	5.70E-01
216	B*50:01-KIR2DS2-m2	13	6	0.31	1.36	0.41	0.75	4.54E-01	0.05	1.05	0.41	0.11	9.09E-01
217	B*51:01-KIR2DS2-m2	34	13	-0.20	0.82	0.28	-0.72	4.71E-01	-0.19	0.83	0.28	-0.66	5.11E-01
218	B*52:01-KIR2DS2-m2	8	2	-0.33	0.72	0.71	-0.46	6.44E-01	0.42	1.53	0.71	0.59	5.53E-01
219	B*53:01-KIR2DS2-m2	5	3	0.34	1.41	0.58	0.59	5.54E-01	0.54	1.72	0.58	0.93	3.53E-01
220	B*55:01-KIR2DS2-m1	16	5	-0.84	0.43	0.58	-1.44	1.49E-01	-0.45	0.64	0.58	-0.78	4.36E-01
221	B*56:01-KIR2DS2-m2	5	1	-0.15	0.86	1.00	-0.15	8.82E-01	1.36	3.89	1.01	1.35	1.77E-01
222	B*57:01-KIR2DS2-m1	7	2	-0.77	0.46	0.87	-0.88	3.79E-01	-0.80	0.45	0.87	-0.92	3.59E-01
223	B*58:01-KIR2DS2-m1	6	2	-2.11	0.12	1.01	-2.10	3.61E-02	-3.78	0.02	1.02	-3.72	2.01E-04
224	R10-KIR2DS2-m2	48	20	-0.07	0.93	0.23	-0.29	7.68E-01	-0.16	0.85	0.23	-0.69	4.88E-01
225	B*07:02-KIR2DS3-m1	21	14	0.34	1.41	0.34	1.00	3.15E-01	0.43	1.54	0.34	1.25	2.10E-01
226	B*08:01-KIR2DS3-m2	63	40	0.21	1.23	0.17	1.23	2.18E-01	0.08	1.08	0.17	0.47	6.41E-01
227	B*13:02-KIR2DS3-m1	6	1	-1.31	0.27	1.08	-1.22	2.24E-01	-1.37	0.25	1.08	-1.27	2.03E-01
228	B*14:01-KIR2DS3-m2	6	0										
229	B*14:02-KIR2DS3-m1	8	3	0.48	1.62	0.66	0.73	4.63E-01	0.72	2.05	0.66	1.10	2.73E-01
230	B*15:01-KIR2DS3-m1	38	30	0.51	1.67	0.25	2.03	4.23E-02	0.36	1.43	0.25	1.42	1.55E-01
231	B*15:18-KIR2DS3-m2	3	0										
232	B*18:01-KIR2DS3-m2	17	15	0.29	1.33	0.26	1.09	2.78E-01	0.12	1.12	0.26	0.44	6.61E-01
233	B*27:05-KIR2DS3-m2	16	7	-0.33	0.72	0.38	-0.86	3.91E-01	-0.23	0.79	0.38	-0.61	5.45E-01
234	B*35:01-KIR2DS3-m1	15	7	-0.50	0.61	0.45	-1.12	2.64E-01	-0.24	0.79	0.45	-0.53	5.93E-01
235	B*35:03-KIR2DS3-m2	6	7	0.28	1.32	0.38	0.73	4.63E-01	0.40	1.49	0.38	1.05	2.93E-01
236	B*35:08-KIR2DS3-m2	1	0										
237	B*37:01-KIR2DS3-m2	6	0										

238	B*38:01-KIR2DS3-m1	9	5	-0.44	0.64	0.53	-0.84	4.01E-01	-0.74	0.48	0.53	-1.41	1.58E-01
239	B*39:01-KIR2DS3-m2	8	4	0.17	1.19	0.50	0.34	7.31E-01	0.64	1.90	0.50	1.28	2.02E-01
240	B*39:06-KIR2DS3-m1	7	4	-0.61	0.54	0.60	-1.01	3.13E-01	-0.72	0.49	0.60	-1.19	2.35E-01
241	B*40:01-KIR2DS3-m2	38	17	-0.08	0.92	0.25	-0.32	7.48E-01	0.08	1.08	0.25	0.31	7.55E-01
242	B*40:02-KIR2DS3-m2	6	1	-1.10	0.33	1.00	-1.09	2.74E-01	-0.17	0.84	1.00	-0.17	8.62E-01
243	B*41:01-KIR2DS3-m2	1	1	0.24	1.27	1.00	0.24	8.12E-01	1.46	4.31	1.01	1.45	1.47E-01
244	B*44:02-KIR2DS3-m1	32	14	-0.39	0.68	0.33	-1.20	2.31E-01	-0.39	0.68	0.33	-1.18	2.38E-01
245	B*44:03-KIR2DS3-m1	12	4	-0.30	0.74	0.57	-0.52	6.04E-01	-0.17	0.85	0.57	-0.29	7.71E-01
246	B*45:01-KIR2DS3-m2	1	1	0.90	2.46	1.00	0.90	3.69E-01	-0.45	0.63	1.00	-0.45	6.51E-01
247	B*49:01-KIR2DS3-m1	3	2	0.73	2.08	0.83	0.89	3.73E-01	-0.19	0.82	0.83	-0.23	8.16E-01
248	B*50:01-KIR2DS3-m2	5	2	0.19	1.21	0.71	0.27	7.91E-01	1.02	2.77	0.71	1.43	1.52E-01
249	B*51:01-KIR2DS3-m1	14	12	1.17	3.23	0.42	2.81	4.97E-03	0.84	2.32	0.42	2.02	4.33E-02
250	B*52:01-KIR2DS3-m2	4	2	-0.05	0.95	0.71	-0.08	9.39E-01	0.50	1.64	0.71	0.70	4.87E-01
251	B*53:01-KIR2DS3-m2	3	1	-0.25	0.78	1.00	-0.25	8.03E-01	0.66	1.93	1.00	0.66	5.12E-01
252	B*55:01-KIR2DS3-m1	10	4	-0.20	0.82	0.61	-0.33	7.43E-01	-0.39	0.68	0.61	-0.63	5.28E-01
253	B*56:01-KIR2DS3-m2	3	0										
254	B*57:01-KIR2DS3-m1	4	1	0.04	1.04	1.10	0.03	9.72E-01	-0.28	0.76	1.10	-0.25	8.01E-01
255	B*58:01-KIR2DS3-m2	3	0										
256	R10-KIR2DS3-m1	24	14	0.39	1.48	0.35	1.12	2.63E-01	0.50	1.64	0.35	1.41	1.58E-01
257	B*07:02-KIR2DS4-m2	98	42	-0.18	0.83	0.16	-1.13	2.56E-01	-0.25	0.78	0.16	-1.56	1.20E-01
258	B*08:01-KIR2DS4-m2	247	135	0.09	1.09	0.11	0.85	3.95E-01	0.04	1.04	0.11	0.34	7.36E-01
259	B*13:02-KIR2DS4-m2	20	8	-0.19	0.83	0.36	-0.52	6.00E-01	0.48	1.61	0.36	1.33	1.85E-01
260	B*14:01-KIR2DS4-m2	11	5	-0.09	0.92	0.45	-0.20	8.45E-01	-0.32	0.73	0.45	-0.70	4.82E-01
261	B*14:02-KIR2DS4-m2	34	15	0.06	1.06	0.26	0.21	8.31E-01	-0.04	0.96	0.26	-0.15	8.79E-01
262	B*15:01-KIR2DS4-m2	195	92	-0.10	0.90	0.12	-0.85	3.98E-01	-0.20	0.82	0.12	-1.72	8.51E-02
263	B*15:18-KIR2DS4-m2	8	4	0.45	1.57	0.50	0.89	3.73E-01	0.77	2.16	0.50	1.53	1.26E-01
264	B*18:01-KIR2DS4-m2	81	63	0.40	1.50	0.14	2.93	3.37E-03	0.19	1.21	0.14	1.40	1.61E-01
265	B*27:05-KIR2DS4-m2	46	21	-0.18	0.84	0.22	-0.80	4.26E-01	-0.13	0.88	0.22	-0.57	5.68E-01
266	B*35:01-KIR2DS4-m1	45	26	-0.53	0.59	0.60	-0.89	3.75E-01	0.08	1.09	0.60	0.14	8.91E-01
267	B*35:03-KIR2DS4-m1	14	13	1.32	3.76	1.06	1.24	2.14E-01	0.98	2.66	1.07	0.92	3.58E-01
268	B*35:08-KIR2DS4-m2	5	5	0.32	1.38	0.45	0.71	4.76E-01	0.52	1.69	0.45	1.16	2.48E-01

269	B*37:01-KIR2DS4-m2	19	3	-1.01	0.36	0.58	-1.74	8.13E-02	-1.25	0.29	0.58	-2.16	3.11E-02
270	B*38:01-KIR2DS4-m2	22	21	0.22	1.24	0.22	0.97	3.31E-01	0.00	1.00	0.23	0.00	9.98E-01
271	B*39:01-KIR2DS4-m2	19	9	-0.05	0.95	0.34	-0.15	8.78E-01	0.50	1.65	0.34	1.48	1.39E-01
272	B*39:06-KIR2DS4-m2	20	13	0.04	1.05	0.28	0.16	8.76E-01	0.16	1.17	0.28	0.56	5.78E-01
273	B*40:01-KIR2DS4-m1	106	61	1.23	3.41	0.76	1.62	1.06E-01	0.92	2.50	0.76	1.21	2.28E-01
274	B*40:02-KIR2DS4-m2	23	4	-0.76	0.47	0.50	-1.51	1.32E-01	-0.08	0.92	0.51	-0.16	8.76E-01
275	B*41:01-KIR2DS4-m2	7	2	-0.40	0.67	0.71	-0.56	5.76E-01	-0.06	0.94	0.71	-0.09	9.31E-01
276	B*44:02-KIR2DS4-m1	125	61	1.76	5.79	1.04	1.70	8.99E-02	1.23	3.41	1.04	1.18	2.37E-01
277	B*44:03-KIR2DS4-m2	37	18	0.01	1.01	0.24	0.06	9.51E-01	0.06	1.06	0.24	0.26	7.97E-01
278	B*45:01-KIR2DS4-m2	12	4	-0.06	0.94	0.50	-0.12	9.08E-01	-0.34	0.72	0.50	-0.67	5.06E-01
279	B*49:01-KIR2DS4-m2	19	7	0.30	1.36	0.38	0.79	4.27E-01	0.23	1.26	0.38	0.61	5.42E-01
280	B*50:01-KIR2DS4-m1	18	9	0.01	1.01	0.82	0.01	9.90E-01	-0.57	0.57	0.82	-0.70	4.87E-01
281	B*51:01-KIR2DS4-m2	72	25	-0.34	0.71	0.21	-1.64	1.02E-01	-0.30	0.74	0.21	-1.48	1.40E-01
282	B*52:01-KIR2DS4-m2	10	2	-0.50	0.61	0.71	-0.70	4.82E-01	0.11	1.12	0.71	0.16	8.73E-01
283	B*53:01-KIR2DS4-m2	8	4	0.31	1.36	0.50	0.61	5.39E-01	0.27	1.30	0.50	0.53	5.99E-01
284	B*55:01-KIR2DS4-m2	28	13	-0.02	0.98	0.28	-0.07	9.41E-01	0.03	1.03	0.28	0.11	9.16E-01
285	B*56:01-KIR2DS4-m2	10	3	-0.22	0.80	0.58	-0.39	7.00E-01	0.56	1.75	0.58	0.96	3.36E-01
286	B*57:01-KIR2DS4-m2	15	6	-0.11	0.89	0.41	-0.28	7.83E-01	0.05	1.05	0.41	0.12	9.03E-01
287	B*58:01-KIR2DS4-m2	6	4	0.17	1.19	0.50	0.34	7.33E-01	0.17	1.19	0.51	0.34	7.37E-01
288	R10-KIR2DS4-m1	87	36	-0.72	0.49	0.59	-1.23	2.19E-01	-0.95	0.38	0.59	-1.62	1.04E-01
289	B*07:02-KIR2DS5-m2	27	13	0.06	1.06	0.28	0.20	8.38E-01	-0.15	0.86	0.28	-0.53	5.95E-01
290	B*08:01-KIR2DS5-m2	87	54	0.24	1.27	0.15	1.65	9.84E-02	0.24	1.27	0.15	1.65	9.92E-02
291	B*13:02-KIR2DS5-m2	6	3	-0.20	0.82	0.58	-0.35	7.26E-01	0.46	1.59	0.58	0.79	4.27E-01
292	B*14:01-KIR2DS5-m1	1	2	0.62	1.85	0.92	0.67	5.03E-01	1.28	3.58	0.93	1.38	1.68E-01
293	B*14:02-KIR2DS5-m1	8	3	-0.13	0.88	0.65	-0.19	8.47E-01	-0.15	0.86	0.65	-0.23	8.17E-01
294	B*15:01-KIR2DS5-m1	60	35	0.20	1.22	0.24	0.82	4.13E-01	-0.06	0.95	0.24	-0.23	8.18E-01
295	B*15:18-KIR2DS5-m2	3	1	-0.06	0.94	1.00	-0.06	9.51E-01	0.70	2.01	1.00	0.70	4.87E-01
296	B*18:01-KIR2DS5-m2	25	21	0.56	1.75	0.22	2.48	1.30E-02	0.33	1.39	0.23	1.47	1.43E-01
297	B*27:05-KIR2DS5-m1	11	6	0.42	1.52	0.49	0.86	3.91E-01	0.32	1.38	0.49	0.66	5.11E-01
298	B*35:01-KIR2DS5-m2	13	14	0.24	1.27	0.27	0.86	3.87E-01	0.08	1.08	0.27	0.29	7.74E-01
299	B*35:03-KIR2DS5-m1	7	2	-1.20	0.30	0.77	-1.55	1.20E-01	-1.12	0.33	0.77	-1.44	1.48E-01

300	B*35:08-KIR2DS5-m1	4	1	-1.45	0.24	1.12	-1.29	1.98E-01	-0.68	0.51	1.12	-0.60	5.48E-01
301	B*37:01-KIR2DS5-m2	8	1	-1.13	0.32	1.00	-1.13	2.60E-01	-0.79	0.45	1.00	-0.79	4.27E-01
302	B*38:01-KIR2DS5-m1	6	6	-0.25	0.78	0.50	-0.50	6.18E-01	-0.41	0.67	0.50	-0.82	4.13E-01
303	B*39:01-KIR2DS5-m2	2	2	0.58	1.79	0.71	0.82	4.14E-01	0.88	2.42	0.71	1.24	2.13E-01
304	B*39:06-KIR2DS5-m2	4	6	0.60	1.81	0.41	1.44	1.49E-01	0.74	2.09	0.41	1.79	7.31E-02
305	B*40:01-KIR2DS5-m2	37	18	0.08	1.08	0.24	0.33	7.40E-01	0.18	1.20	0.24	0.76	4.49E-01
306	B*40:02-KIR2DS5-m1	5	2	1.31	3.72	1.01	1.31	1.92E-01	0.93	2.53	1.01	0.92	3.57E-01
307	B*41:01-KIR2DS5-m2	1	1	1.02	2.79	1.00	1.02	3.06E-01	0.28	1.32	1.00	0.28	7.82E-01
308	B*44:02-KIR2DS5-m1	45	16	-0.54	0.58	0.31	-1.74	8.24E-02	-0.19	0.82	0.31	-0.62	5.36E-01
309	B*44:03-KIR2DS5-m1	14	6	-0.54	0.58	0.51	-1.06	2.87E-01	-0.22	0.80	0.51	-0.44	6.59E-01
310	B*45:01-KIR2DS5-m2	5	0										
311	B*49:01-KIR2DS5-m1	7	2	-0.88	0.41	0.82	-1.07	2.83E-01	-0.48	0.62	0.83	-0.58	5.63E-01
312	B*50:01-KIR2DS5-m1	8	5	0.45	1.56	0.62	0.73	4.68E-01	-0.17	0.85	0.62	-0.27	7.88E-01
313	B*51:01-KIR2DS5-m1	23	7	-0.06	0.94	0.46	-0.14	8.92E-01	0.19	1.21	0.46	0.42	6.73E-01
314	B*52:01-KIR2DS5-m2	4	1	-0.29	0.75	1.00	-0.29	7.75E-01	0.15	1.16	1.00	0.15	8.79E-01
315	B*53:01-KIR2DS5-m2	2	3	1.06	2.89	0.58	1.83	6.70E-02	0.66	1.93	0.58	1.13	2.58E-01
316	B*55:01-KIR2DS5-m1	9	1	-1.66	0.19	1.05	-1.59	1.12E-01	-1.77	0.17	1.05	-1.69	9.06E-02
317	B*56:01-KIR2DS5-m1	6	1	-0.99	0.37	1.16	-0.86	3.92E-01	-1.42	0.24	1.16	-1.23	2.20E-01
318	B*57:01-KIR2DS5-m1	5	2	-0.19	0.83	0.87	-0.21	8.30E-01	-0.35	0.70	0.87	-0.40	6.87E-01
319	B*58:01-KIR2DS5-m1	4	1	-0.10	0.91	1.16	-0.09	9.32E-01	-1.25	0.29	1.17	-1.07	2.85E-01
320	R10-KIR2DS5-m1	34	17	-0.04	0.96	0.34	-0.12	9.02E-01	0.09	1.09	0.34	0.25	8.02E-01
321	B*07:02-KIR3DL1-m2	98	42	-0.18	0.83	0.16	-1.13	2.56E-01	-0.25	0.78	0.16	-1.56	1.20E-01
322	B*08:01-KIR3DL1-m2	247	135	0.09	1.09	0.11	0.85	3.95E-01	0.04	1.04	0.11	0.34	7.36E-01
323	B*13:02-KIR3DL1-m2	20	8	-0.19	0.83	0.36	-0.52	6.00E-01	0.48	1.61	0.36	1.33	1.85E-01
324	B*14:01-KIR3DL1-m2	11	5	-0.09	0.92	0.45	-0.20	8.45E-01	-0.32	0.73	0.45	-0.70	4.82E-01
325	B*14:02-KIR3DL1-m2	34	15	0.06	1.06	0.26	0.21	8.31E-01	-0.04	0.96	0.26	-0.15	8.79E-01
326	B*15:01-KIR3DL1-m2	195	92	-0.10	0.90	0.12	-0.85	3.98E-01	-0.20	0.82	0.12	-1.72	8.51E-02
327	B*15:18-KIR3DL1-m2	8	4	0.45	1.57	0.50	0.89	3.73E-01	0.77	2.16	0.50	1.53	1.26E-01
328	B*18:01-KIR3DL1-m2	81	63	0.40	1.50	0.14	2.93	3.37E-03	0.19	1.21	0.14	1.40	1.61E-01
329	B*27:05-KIR3DL1-m2	46	21	-0.18	0.84	0.22	-0.80	4.26E-01	-0.13	0.88	0.22	-0.57	5.68E-01
330	B*35:01-KIR3DL1-m1	45	26	-0.52	0.60	0.60	-0.87	3.87E-01	0.09	1.09	0.60	0.14	8.87E-01

331	B*35:03-KIR3DL1-m1	14	13	1.34	3.81	1.06	1.26	2.09E-01	0.98	2.67	1.07	0.92	3.57E-01
332	B*35:08-KIR3DL1-m2	6	5	0.27	1.31	0.45	0.60	5.47E-01	0.51	1.67	0.45	1.13	2.59E-01
333	B*37:01-KIR3DL1-m2	19	3	-1.01	0.36	0.58	-1.74	8.13E-02	-1.25	0.29	0.58	-2.16	3.11E-02
334	B*38:01-KIR3DL1-m2	22	21	0.22	1.24	0.22	0.97	3.31E-01	0.00	1.00	0.23	0.00	9.98E-01
335	B*39:01-KIR3DL1-m2	19	9	-0.05	0.95	0.34	-0.15	8.78E-01	0.50	1.65	0.34	1.48	1.39E-01
336	B*39:06-KIR3DL1-m2	20	13	0.04	1.05	0.28	0.16	8.76E-01	0.16	1.17	0.28	0.56	5.78E-01
337	B*40:01-KIR3DL1-m1	106	61	1.24	3.46	0.76	1.64	1.02E-01	0.92	2.51	0.76	1.21	2.26E-01
338	B*40:02-KIR3DL1-m2	23	4	-0.76	0.47	0.50	-1.51	1.32E-01	-0.08	0.92	0.51	-0.16	8.76E-01
339	B*41:01-KIR3DL1-m2	7	2	-0.40	0.67	0.71	-0.56	5.76E-01	-0.06	0.94	0.71	-0.09	9.31E-01
340	B*44:02-KIR3DL1-m1	125	61	1.77	5.88	1.04	1.71	8.72E-02	1.23	3.42	1.04	1.18	2.36E-01
341	B*44:03-KIR3DL1-m2	37	18	0.01	1.01	0.24	0.06	9.51E-01	0.06	1.06	0.24	0.26	7.97E-01
342	B*45:01-KIR3DL1-m2	12	4	-0.06	0.94	0.50	-0.12	9.08E-01	-0.34	0.72	0.50	-0.67	5.06E-01
343	B*49:01-KIR3DL1-m2	19	7	0.30	1.36	0.38	0.79	4.27E-01	0.23	1.26	0.38	0.61	5.42E-01
344	B*50:01-KIR3DL1-m1	18	9	0.02	1.02	0.82	0.03	9.78E-01	-0.57	0.57	0.82	-0.69	4.89E-01
345	B*51:01-KIR3DL1-m2	72	25	-0.34	0.71	0.21	-1.64	1.02E-01	-0.30	0.74	0.21	-1.48	1.40E-01
346	B*52:01-KIR3DL1-m2	10	2	-0.50	0.61	0.71	-0.70	4.82E-01	0.11	1.12	0.71	0.16	8.73E-01
347	B*53:01-KIR3DL1-m2	8	4	0.31	1.36	0.50	0.61	5.39E-01	0.27	1.30	0.50	0.53	5.99E-01
348	B*55:01-KIR3DL1-m2	28	13	-0.02	0.98	0.28	-0.07	9.41E-01	0.03	1.03	0.28	0.11	9.16E-01
349	B*56:01-KIR3DL1-m2	10	3	-0.22	0.80	0.58	-0.39	7.00E-01	0.56	1.75	0.58	0.96	3.36E-01
350	B*57:01-KIR3DL1-m2	15	6	-0.11	0.89	0.41	-0.28	7.83E-01	0.05	1.05	0.41	0.12	9.03E-01
351	B*58:01-KIR3DL1-m2	6	4	0.17	1.19	0.50	0.34	7.33E-01	0.17	1.19	0.51	0.34	7.37E-01
352	R10-KIR3DL1-m1	88	36	-0.81	0.44	0.59	-1.38	1.66E-01	-0.98	0.38	0.59	-1.66	9.65E-02
353	B*07:02-KIR3DL2-m2	103	46	-0.14	0.87	0.16	-0.87	3.85E-01	-0.21	0.81	0.16	-1.35	1.77E-01
354	B*08:01-KIR3DL2-m2	262	141	0.09	1.09	0.10	0.85	3.98E-01	0.05	1.05	0.10	0.46	6.43E-01
355	B*13:02-KIR3DL2-m2	21	8	-0.23	0.79	0.36	-0.65	5.18E-01	0.46	1.58	0.36	1.27	2.03E-01
356	B*14:01-KIR3DL2-m2	11	5	-0.09	0.92	0.45	-0.20	8.45E-01	-0.32	0.73	0.45	-0.70	4.82E-01
357	B*14:02-KIR3DL2-m2	34	15	0.06	1.06	0.26	0.21	8.31E-01	-0.04	0.96	0.26	-0.15	8.79E-01
358	B*15:01-KIR3DL2-m2	203	101	-0.02	0.98	0.11	-0.21	8.38E-01	-0.15	0.86	0.11	-1.28	2.02E-01
359	B*15:18-KIR3DL2-m2	8	4	0.45	1.57	0.50	0.89	3.73E-01	0.77	2.16	0.50	1.53	1.26E-01
360	B*18:01-KIR3DL2-m2	83	64	0.40	1.49	0.14	2.93	3.35E-03	0.16	1.18	0.14	1.20	2.30E-01
361	B*27:05-KIR3DL2-m2	47	22	-0.15	0.86	0.22	-0.68	4.99E-01	-0.10	0.91	0.22	-0.45	6.50E-01

362	B*35:01-KIR3DL2-m2	48	30	-0.06	0.94	0.19	-0.30	7.65E-01	0.04	1.04	0.19	0.22	8.25E-01
363	B*35:03-KIR3DL2-m2	17	14	0.14	1.15	0.27	0.52	6.06E-01	0.07	1.07	0.27	0.25	8.06E-01
364	B*35:08-KIR3DL2-m2	6	5	0.27	1.31	0.45	0.60	5.47E-01	0.51	1.67	0.45	1.13	2.59E-01
365	B*37:01-KIR3DL2-m2	19	3	-1.01	0.36	0.58	-1.74	8.13E-02	-1.25	0.29	0.58	-2.16	3.11E-02
366	B*38:01-KIR3DL2-m2	22	21	0.22	1.24	0.22	0.97	3.31E-01	0.00	1.00	0.23	0.00	9.98E-01
367	B*39:01-KIR3DL2-m2	19	9	-0.05	0.95	0.34	-0.15	8.78E-01	0.50	1.65	0.34	1.48	1.39E-01
368	B*39:06-KIR3DL2-m2	20	14	0.12	1.12	0.27	0.43	6.66E-01	0.22	1.25	0.27	0.81	4.20E-01
369	B*40:01-KIR3DL2-m2	114	63	0.02	1.02	0.14	0.12	9.05E-01	0.09	1.10	0.14	0.67	5.02E-01
370	B*40:02-KIR3DL2-m2	24	4	-0.78	0.46	0.50	-1.56	1.20E-01	-0.08	0.92	0.51	-0.16	8.71E-01
371	B*41:01-KIR3DL2-m2	8	3	-0.10	0.90	0.58	-0.18	8.58E-01	0.04	1.04	0.58	0.07	9.46E-01
372	B*44:02-KIR3DL2-m2	134	62	-0.12	0.89	0.14	-0.88	3.78E-01	0.06	1.06	0.14	0.42	6.77E-01
373	B*44:03-KIR3DL2-m2	37	19	0.03	1.03	0.24	0.13	8.98E-01	0.10	1.10	0.24	0.41	6.80E-01
374	B*45:01-KIR3DL2-m2	13	4	-0.14	0.87	0.50	-0.27	7.88E-01	-0.45	0.64	0.50	-0.90	3.68E-01
375	B*49:01-KIR3DL2-m2	19	8	0.32	1.38	0.36	0.89	3.73E-01	0.21	1.23	0.36	0.58	5.59E-01
376	B*50:01-KIR3DL2-m2	20	11	0.24	1.27	0.31	0.78	4.34E-01	0.03	1.03	0.31	0.11	9.12E-01
377	B*51:01-KIR3DL2-m2	75	25	-0.35	0.70	0.21	-1.72	8.59E-02	-0.31	0.73	0.21	-1.50	1.34E-01
378	B*52:01-KIR3DL2-m2	10	2	-0.50	0.61	0.71	-0.70	4.82E-01	0.11	1.12	0.71	0.16	8.73E-01
379	B*53:01-KIR3DL2-m2	8	4	0.31	1.36	0.50	0.61	5.39E-01	0.27	1.30	0.50	0.53	5.99E-01
380	B*55:01-KIR3DL2-m2	28	13	-0.02	0.98	0.28	-0.07	9.41E-01	0.03	1.03	0.28	0.11	9.16E-01
381	B*56:01-KIR3DL2-m2	12	4	-0.04	0.96	0.50	-0.07	9.43E-01	0.76	2.14	0.50	1.51	1.32E-01
382	B*57:01-KIR3DL2-m2	15	6	-0.11	0.89	0.41	-0.28	7.83E-01	0.05	1.05	0.41	0.12	9.03E-01
383	B*58:01-KIR3DL2-m2	7	4	0.15	1.16	0.50	0.29	7.69E-01	0.16	1.18	0.50	0.32	7.48E-01
384	R10-KIR3DL2-m2	92	40	-0.14	0.87	0.17	-0.84	4.02E-01	-0.14	0.87	0.17	-0.83	4.09E-01
385	B*07:02-KIR3DS1-m1	28	17	0.50	1.65	0.32	1.55	1.22E-01	0.22	1.25	0.33	0.68	5.00E-01
386	B*08:01-KIR3DS1-m2	104	57	0.08	1.08	0.14	0.57	5.72E-01	0.07	1.08	0.14	0.51	6.08E-01
387	B*13:02-KIR3DS1-m2	6	3	-0.20	0.82	0.58	-0.35	7.26E-01	0.46	1.59	0.58	0.79	4.27E-01
388	B*14:01-KIR3DS1-m1	3	2	0.20	1.22	0.92	0.22	8.29E-01	0.99	2.69	0.92	1.07	2.84E-01
389	B*14:02-KIR3DS1-m2	10	4	0.26	1.30	0.50	0.53	5.99E-01	0.10	1.10	0.50	0.19	8.49E-01
390	B*15:01-KIR3DS1-m2	69	43	0.23	1.26	0.16	1.42	1.56E-01	-0.05	0.96	0.16	-0.28	7.79E-01
391	B*15:18-KIR3DS1-m2	3	1	-0.06	0.94	1.00	-0.06	9.51E-01	0.70	2.01	1.00	0.70	4.87E-01
392	B*18:01-KIR3DS1-m2	27	23	0.48	1.62	0.22	2.24	2.54E-02	0.21	1.24	0.22	0.98	3.25E-01

393	B*27:05-KIR3DS1-m1	16	10	0.29	1.34	0.44	0.67	5.05E-01	0.11	1.11	0.44	0.24	8.07E-01
394	B*35:01-KIR3DS1-m2	17	15	0.10	1.11	0.26	0.39	6.98E-01	0.07	1.08	0.26	0.28	7.79E-01
395	B*35:03-KIR3DS1-m1	9	5	-0.64	0.53	0.57	-1.13	2.57E-01	-0.34	0.71	0.57	-0.59	5.52E-01
396	B*35:08-KIR3DS1-m1	4	1	-1.38	0.25	1.12	-1.23	2.18E-01	-0.62	0.54	1.12	-0.55	5.81E-01
397	B*37:01-KIR3DS1-m2	9	1	-1.22	0.30	1.00	-1.22	2.24E-01	-0.85	0.43	1.00	-0.85	3.97E-01
398	B*38:01-KIR3DS1-m1	8	6	-0.46	0.63	0.49	-0.93	3.52E-01	-0.49	0.61	0.50	-1.00	3.18E-01
399	B*39:01-KIR3DS1-m2	4	4	1.05	2.86	0.50	2.09	3.68E-02	1.37	3.92	0.50	2.71	6.75E-03
400	B*39:06-KIR3DS1-m2	7	6	0.11	1.12	0.41	0.27	7.86E-01	0.42	1.52	0.41	1.01	3.13E-01
401	B*40:01-KIR3DS1-m2	43	23	0.10	1.10	0.21	0.46	6.45E-01	0.22	1.24	0.22	1.00	3.17E-01
402	B*40:02-KIR3DS1-m1	5	2	1.47	4.37	1.01	1.47	1.43E-01	1.10	3.01	1.01	1.10	2.73E-01
403	B*41:01-KIR3DS1-m2	1	1	1.02	2.79	1.00	1.02	3.06E-01	0.28	1.32	1.00	0.28	7.82E-01
404	B*44:02-KIR3DS1-m1	55	21	-0.56	0.57	0.29	-1.91	5.56E-02	-0.11	0.89	0.29	-0.39	6.96E-01
405	B*44:03-KIR3DS1-m1	17	5	-0.92	0.40	0.53	-1.73	8.35E-02	-0.60	0.55	0.53	-1.12	2.62E-01
406	B*45:01-KIR3DS1-m2	4	0										
407	B*49:01-KIR3DS1-m1	7	3	-0.44	0.65	0.74	-0.59	5.53E-01	-0.56	0.57	0.74	-0.76	4.49E-01
408	B*50:01-KIR3DS1-m2	8	4	0.61	1.84	0.50	1.21	2.25E-01	0.97	2.63	0.50	1.92	5.48E-02
409	B*51:01-KIR3DS1-m1	23	10	0.43	1.54	0.42	1.02	3.07E-01	0.28	1.32	0.42	0.65	5.13E-01
410	B*52:01-KIR3DS1-m1	4	1	0.33	1.39	1.42	0.23	8.17E-01	-0.24	0.78	1.42	-0.17	8.64E-01
411	B*53:01-KIR3DS1-m2	2	2	1.02	2.76	0.71	1.43	1.52E-01	0.73	2.08	0.71	1.03	3.02E-01
412	B*55:01-KIR3DS1-m1	14	3	-1.11	0.33	0.67	-1.66	9.64E-02	-1.30	0.27	0.67	-1.95	5.14E-02
413	B*56:01-KIR3DS1-m1	7	1	-1.12	0.33	1.16	-0.97	3.34E-01	-1.40	0.25	1.16	-1.21	2.27E-01
414	B*57:01-KIR3DS1-m1	7	2	-0.24	0.79	0.87	-0.27	7.86E-01	-0.40	0.67	0.87	-0.46	6.47E-01
415	B*58:01-KIR3DS1-m2	3	0										
416	R10-KIR3DS1-m1	37	19	-0.17	0.84	0.33	-0.52	6.03E-01	0.07	1.07	0.34	0.20	8.39E-01
417	B*07:02-KIR2DP1-m2	102	46	-0.13	0.87	0.16	-0.86	3.91E-01	-0.21	0.81	0.16	-1.35	1.77E-01
418	B*08:01-KIR2DP1-m1	258	135	-0.39	0.68	0.65	-0.60	5.49E-01	-0.61	0.54	0.65	-0.94	3.47E-01
419	B*13:02-KIR2DP1-m2	21	8	-0.23	0.79	0.36	-0.65	5.18E-01	0.46	1.58	0.36	1.27	2.03E-01
420	B*14:01-KIR2DP1-m2	11	5	-0.09	0.92	0.45	-0.20	8.45E-01	-0.32	0.73	0.45	-0.70	4.82E-01
421	B*14:02-KIR2DP1-m2	30	15	0.10	1.10	0.26	0.38	7.05E-01	0.00	1.00	0.26	0.01	9.94E-01
422	B*15:01-KIR2DP1-m2	200	100	-0.02	0.98	0.12	-0.20	8.43E-01	-0.15	0.86	0.12	-1.29	1.96E-01
423	B*15:18-KIR2DP1-m2	7	4	0.52	1.69	0.50	1.04	2.99E-01	0.89	2.44	0.50	1.77	7.64E-02

424	B*18:01-KIR2DP1-m2	82	62	0.39	1.48	0.14	2.84	4.54E-03	0.15	1.16	0.14	1.09	2.76E-01
425	B*27:05-KIR2DP1-m2	47	21	-0.18	0.83	0.22	-0.81	4.16E-01	-0.13	0.88	0.22	-0.58	5.63E-01
426	B*35:01-KIR2DP1-m2	47	30	-0.05	0.96	0.19	-0.24	8.10E-01	0.06	1.06	0.19	0.32	7.52E-01
427	B*35:03-KIR2DP1-m2	17	14	0.14	1.15	0.27	0.52	6.06E-01	0.07	1.07	0.27	0.25	8.06E-01
428	B*35:08-KIR2DP1-m2	6	5	0.27	1.31	0.45	0.60	5.47E-01	0.51	1.67	0.45	1.13	2.59E-01
429	B*37:01-KIR2DP1-m2	18	3	-0.94	0.39	0.58	-1.62	1.04E-01	-1.17	0.31	0.58	-2.02	4.36E-02
430	B*38:01-KIR2DP1-m1	22	17	-0.96	0.38	0.69	-1.39	1.65E-01	-0.24	0.79	0.69	-0.34	7.31E-01
431	B*39:01-KIR2DP1-m2	19	9	-0.05	0.95	0.34	-0.15	8.78E-01	0.50	1.65	0.34	1.48	1.39E-01
432	B*39:06-KIR2DP1-m2	19	13	0.11	1.12	0.28	0.40	6.91E-01	0.16	1.18	0.28	0.58	5.61E-01
433	B*40:01-KIR2DP1-m2	114	63	0.02	1.02	0.14	0.12	9.05E-01	0.09	1.10	0.14	0.67	5.02E-01
434	B*40:02-KIR2DP1-m2	24	4	-0.78	0.46	0.50	-1.56	1.20E-01	-0.08	0.92	0.51	-0.16	8.71E-01
435	B*41:01-KIR2DP1-m2	6	3	0.17	1.18	0.58	0.29	7.71E-01	0.30	1.35	0.58	0.52	6.05E-01
436	B*44:02-KIR2DP1-m2	130	61	-0.12	0.89	0.14	-0.84	4.03E-01	0.05	1.06	0.14	0.39	6.94E-01
437	B*44:03-KIR2DP1-m1	34	18	0.51	1.66	1.08	0.47	6.40E-01	0.83	2.29	1.08	0.77	4.43E-01
438	B*45:01-KIR2DP1-m2	12	3	-0.34	0.71	0.58	-0.59	5.56E-01	-0.65	0.52	0.58	-1.12	2.64E-01
439	B*49:01-KIR2DP1-m2	18	8	0.34	1.40	0.36	0.95	3.42E-01	0.22	1.24	0.36	0.61	5.44E-01
440	B*50:01-KIR2DP1-m2	20	10	0.22	1.24	0.32	0.68	4.99E-01	0.09	1.10	0.32	0.28	7.78E-01
441	B*51:01-KIR2DP1-m2	71	25	-0.34	0.71	0.21	-1.63	1.03E-01	-0.30	0.74	0.21	-1.47	1.42E-01
442	B*52:01-KIR2DP1-m2	9	2	-0.39	0.68	0.71	-0.54	5.86E-01	0.15	1.16	0.71	0.21	8.31E-01
443	B*53:01-KIR2DP1-m2	8	4	0.31	1.36	0.50	0.61	5.39E-01	0.27	1.30	0.50	0.53	5.99E-01
444	B*55:01-KIR2DP1-m2	27	13	0.04	1.05	0.28	0.16	8.75E-01	0.09	1.09	0.28	0.30	7.61E-01
445	B*56:01-KIR2DP1-m2	12	4	-0.04	0.96	0.50	-0.07	9.43E-01	0.76	2.14	0.50	1.51	1.32E-01
446	B*57:01-KIR2DP1-m2	15	6	-0.11	0.89	0.41	-0.28	7.83E-01	0.05	1.05	0.41	0.12	9.03E-01
447	B*58:01-KIR2DP1-m2	7	4	0.15	1.16	0.50	0.29	7.69E-01	0.16	1.18	0.50	0.32	7.48E-01
448	R10-KIR2DP1-m2	91	40	-0.14	0.87	0.17	-0.83	4.07E-01	-0.14	0.87	0.17	-0.82	4.12E-01
449	B*07:02-KIR3DP1-m2	103	46	-0.14	0.87	0.16	-0.87	3.85E-01	-0.21	0.81	0.16	-1.35	1.77E-01
450	B*08:01-KIR3DP1-m2	262	141	0.09	1.09	0.10	0.85	3.98E-01	0.05	1.05	0.10	0.46	6.43E-01
451	B*13:02-KIR3DP1-m2	21	8	-0.23	0.79	0.36	-0.65	5.18E-01	0.46	1.58	0.36	1.27	2.03E-01
452	B*14:01-KIR3DP1-m2	11	5	-0.09	0.92	0.45	-0.20	8.45E-01	-0.32	0.73	0.45	-0.70	4.82E-01
453	B*14:02-KIR3DP1-m2	34	15	0.06	1.06	0.26	0.21	8.31E-01	-0.04	0.96	0.26	-0.15	8.79E-01
454	B*15:01-KIR3DP1-m2	203	101	-0.02	0.98	0.11	-0.21	8.38E-01	-0.15	0.86	0.11	-1.28	2.02E-01

455	B*15:18-KIR3DP1-m2	8	4	0.45	1.57	0.50	0.89	3.73E-01	0.77	2.16	0.50	1.53	1.26E-01
456	B*18:01-KIR3DP1-m2	83	64	0.40	1.49	0.14	2.93	3.35E-03	0.16	1.18	0.14	1.20	2.30E-01
457	B*27:05-KIR3DP1-m2	47	22	-0.15	0.86	0.22	-0.68	4.99E-01	-0.10	0.91	0.22	-0.45	6.50E-01
458	B*35:01-KIR3DP1-m2	48	30	-0.06	0.94	0.19	-0.30	7.65E-01	0.04	1.04	0.19	0.22	8.25E-01
459	B*35:03-KIR3DP1-m2	17	14	0.14	1.15	0.27	0.52	6.06E-01	0.07	1.07	0.27	0.25	8.06E-01
460	B*35:08-KIR3DP1-m2	6	5	0.27	1.31	0.45	0.60	5.47E-01	0.51	1.67	0.45	1.13	2.59E-01
461	B*37:01-KIR3DP1-m2	19	3	-1.01	0.36	0.58	-1.74	8.13E-02	-1.25	0.29	0.58	-2.16	3.11E-02
462	B*38:01-KIR3DP1-m2	22	21	0.22	1.24	0.22	0.97	3.31E-01	0.00	1.00	0.23	0.00	9.98E-01
463	B*39:01-KIR3DP1-m2	19	9	-0.05	0.95	0.34	-0.15	8.78E-01	0.50	1.65	0.34	1.48	1.39E-01
464	B*39:06-KIR3DP1-m2	20	14	0.12	1.12	0.27	0.43	6.66E-01	0.22	1.25	0.27	0.81	4.20E-01
465	B*40:01-KIR3DP1-m2	114	63	0.02	1.02	0.14	0.12	9.05E-01	0.09	1.10	0.14	0.67	5.02E-01
466	B*40:02-KIR3DP1-m2	24	4	-0.78	0.46	0.50	-1.56	1.20E-01	-0.08	0.92	0.51	-0.16	8.71E-01
467	B*41:01-KIR3DP1-m2	8	3	-0.10	0.90	0.58	-0.18	8.58E-01	0.04	1.04	0.58	0.07	9.46E-01
468	B*44:02-KIR3DP1-m2	134	62	-0.12	0.89	0.14	-0.88	3.78E-01	0.06	1.06	0.14	0.42	6.77E-01
469	B*44:03-KIR3DP1-m2	37	19	0.03	1.03	0.24	0.13	8.98E-01	0.10	1.10	0.24	0.41	6.80E-01
470	B*45:01-KIR3DP1-m2	13	4	-0.14	0.87	0.50	-0.27	7.88E-01	-0.45	0.64	0.50	-0.90	3.68E-01
471	B*49:01-KIR3DP1-m2	19	8	0.32	1.38	0.36	0.89	3.73E-01	0.21	1.23	0.36	0.58	5.59E-01
472	B*50:01-KIR3DP1-m2	20	11	0.24	1.27	0.31	0.78	4.34E-01	0.03	1.03	0.31	0.11	9.12E-01
473	B*51:01-KIR3DP1-m2	75	25	-0.35	0.70	0.21	-1.72	8.59E-02	-0.31	0.73	0.21	-1.50	1.34E-01
474	B*52:01-KIR3DP1-m2	10	2	-0.50	0.61	0.71	-0.70	4.82E-01	0.11	1.12	0.71	0.16	8.73E-01
475	B*53:01-KIR3DP1-m2	8	4	0.31	1.36	0.50	0.61	5.39E-01	0.27	1.30	0.50	0.53	5.99E-01
476	B*55:01-KIR3DP1-m2	28	13	-0.02	0.98	0.28	-0.07	9.41E-01	0.03	1.03	0.28	0.11	9.16E-01
477	B*56:01-KIR3DP1-m2	12	4	-0.04	0.96	0.50	-0.07	9.43E-01	0.76	2.14	0.50	1.51	1.32E-01
478	B*57:01-KIR3DP1-m2	15	6	-0.11	0.89	0.41	-0.28	7.83E-01	0.05	1.05	0.41	0.12	9.03E-01
479	B*58:01-KIR3DP1-m2	7	4	0.15	1.16	0.50	0.29	7.69E-01	0.16	1.18	0.50	0.32	7.48E-01
480	R10-KIR3DP1-m2	92	40	-0.14	0.87	0.17	-0.84	4.02E-01	-0.14	0.87	0.17	-0.83	4.09E-01

ESM Table 9. Interaction of HLA-C with KIR ligand numbers with the disease progression without (left panel) and with (right panel) adjustment for confounding effects: allelic frequency (left panel) or interactive frequency (right panel), estimated coefficient, hazard ratio, standard error, Z-score and p-value. P-values, if less than 0.05, are highlighted brown and light blue, if corresponding HR is less than 1 or greater than 1, respectively.

seq	C-KIR-model	Eff. Size		Unadjusted					Adjusted				
		n0	n1	coef	HR	SE	z	p	coef	HR	SE	z	p
1	C*01:02-KIR2DL1-m2	50	21	-0.23	0.79	0.22	-1.05	2.95E-01	0.00	1.00	0.22	-0.01	9.90E-01
2	C*02:02-KIR2DL1-m2	62	18	-0.45	0.64	0.24	-1.85	6.43E-02	-0.35	0.70	0.24	-1.46	1.43E-01
3	C*03:03-KIR2DL1-m1	101	55	0.25	1.28	1.06	0.23	8.17E-01	-0.10	0.91	1.07	-0.09	9.28E-01
4	C*03:04-KIR2DL1-m2	237	122	0.00	1.00	0.11	0.00	9.96E-01	-0.05	0.95	0.11	-0.44	6.58E-01
5	C*04:01-KIR2DL1-m2	87	43	-0.14	0.87	0.16	-0.89	3.74E-01	-0.07	0.93	0.16	-0.42	6.73E-01
6	C*05:01-KIR2DL1-m1	154	86	-0.03	0.97	0.80	-0.03	9.73E-01	-0.37	0.69	0.80	-0.46	6.44E-01
7	C*06:02-KIR2DL1-m1	88	36	-0.27	0.76	0.81	-0.33	7.40E-01	0.43	1.54	0.81	0.53	5.94E-01
8	C*07:01-KIR2DL1-m2	300	157	0.14	1.15	0.10	1.35	1.78E-01	0.11	1.12	0.10	1.12	2.62E-01
9	C*07:02-KIR2DL1-m1	138	64	-0.12	0.88	1.06	-0.12	9.06E-01	-1.44	0.24	1.06	-1.35	1.76E-01
10	C*07:04-KIR2DL1-m2	24	11	-0.07	0.93	0.31	-0.24	8.12E-01	0.30	1.35	0.31	0.98	3.28E-01
11	C*08:02-KIR2DL1-m2	39	20	0.06	1.07	0.23	0.27	7.84E-01	-0.07	0.93	0.23	-0.31	7.56E-01
12	C*12:02-KIR2DL1-m2	9	2	-0.39	0.68	0.71	-0.54	5.86E-01	0.15	1.16	0.71	0.21	8.31E-01
13	C*12:03-KIR2DL1-m2	59	50	0.30	1.34	0.15	1.95	5.07E-02	0.20	1.22	0.15	1.31	1.91E-01
14	C*14:02-KIR2DL1-m2	15	9	0.18	1.20	0.34	0.55	5.85E-01	0.18	1.20	0.34	0.54	5.88E-01
15	C*15:02-KIR2DL1-m2	34	13	-0.46	0.63	0.28	-1.63	1.04E-01	-0.25	0.78	0.28	-0.88	3.79E-01
16	C*15:05-KIR2DL1-m2	9	4	-0.27	0.76	0.50	-0.53	5.94E-01	-0.69	0.50	0.50	-1.37	1.71E-01
17	C*16:01-KIR2DL1-m1	36	15	0.14	1.15	1.09	0.13	8.98E-01	0.55	1.74	1.09	0.51	6.10E-01
18	C*16:02-KIR2DL1-m2	4	6	0.64	1.89	0.41	1.55	1.21E-01	0.60	1.82	0.41	1.45	1.47E-01
19	R10-KIR2DL1-m2	36	20	0.16	1.17	0.23	0.69	4.92E-01	0.15	1.16	0.23	0.65	5.17E-01
20	C*01:02-KIR2DL2-m1	25	6	-0.74	0.48	0.49	-1.51	1.31E-01	-0.49	0.61	0.49	-1.00	3.16E-01

21	C*02:02-KIR2DL2-m2	28	9	-0.35	0.70	0.34	-1.05	2.93E-01	-0.25	0.78	0.34	-0.74	4.57E-01
22	C*03:03-KIR2DL2-m1	54	27	-0.40	0.67	0.29	-1.37	1.71E-01	-0.21	0.81	0.29	-0.72	4.71E-01
23	C*03:04-KIR2DL2-m1	111	61	0.25	1.28	0.22	1.15	2.50E-01	0.34	1.40	0.22	1.56	1.19E-01
24	C*04:01-KIR2DL2-m2	43	20	-0.16	0.85	0.23	-0.70	4.84E-01	-0.06	0.94	0.23	-0.25	7.99E-01
25	C*05:01-KIR2DL2-m2	71	46	0.15	1.16	0.16	0.93	3.54E-01	-0.04	0.96	0.16	-0.25	8.06E-01
26	C*06:02-KIR2DL2-m2	46	18	-0.14	0.87	0.24	-0.58	5.64E-01	-0.24	0.79	0.24	-0.98	3.28E-01
27	C*07:01-KIR2DL2-m2	154	88	0.16	1.17	0.12	1.31	1.89E-01	0.09	1.09	0.12	0.72	4.73E-01
28	C*07:02-KIR2DL2-m2	68	33	-0.10	0.90	0.18	-0.56	5.75E-01	-0.03	0.97	0.18	-0.16	8.71E-01
29	C*07:04-KIR2DL2-m1	14	4	-0.58	0.56	0.64	-0.91	3.60E-01	-0.55	0.58	0.64	-0.86	3.90E-01
30	C*08:02-KIR2DL2-m2	22	6	-0.34	0.71	0.41	-0.84	4.04E-01	-0.30	0.74	0.41	-0.73	4.66E-01
31	C*12:02-KIR2DL2-m2	7	2	-0.19	0.82	0.71	-0.27	7.85E-01	0.48	1.61	0.71	0.67	5.05E-01
32	C*12:03-KIR2DL2-m2	32	29	0.45	1.57	0.19	2.34	1.94E-02	0.18	1.20	0.19	0.95	3.44E-01
33	C*14:02-KIR2DL2-m2	11	6	0.05	1.05	0.41	0.11	9.11E-01	0.21	1.23	0.41	0.50	6.17E-01
34	C*15:02-KIR2DL2-m2	10	4	-0.36	0.70	0.50	-0.71	4.79E-01	-0.37	0.69	0.50	-0.74	4.58E-01
35	C*15:05-KIR2DL2-m2	7	3	-0.38	0.68	0.58	-0.65	5.14E-01	-0.84	0.43	0.58	-1.45	1.48E-01
36	C*16:01-KIR2DL2-m1	17	9	0.72	2.06	0.51	1.40	1.61E-01	0.59	1.81	0.51	1.15	2.48E-01
37	C*16:02-KIR2DL2-m1	4	3	-0.45	0.64	0.82	-0.54	5.89E-01	-0.79	0.45	0.82	-0.96	3.39E-01
38	R10-KIR2DL2-m1	30	10	-0.79	0.46	0.46	-1.72	8.62E-02	-1.29	0.27	0.46	-2.80	5.07E-03
39	C*01:02-KIR2DL3-m1	48	21	-0.18	0.84	1.04	-0.17	8.62E-01	-0.43	0.65	1.04	-0.41	6.82E-01
40	C*02:02-KIR2DL3-m2	59	17	-0.45	0.64	0.25	-1.82	6.93E-02	-0.38	0.69	0.25	-1.52	1.27E-01
41	C*03:03-KIR2DL3-m1	95	52	0.47	1.61	0.55	0.86	3.88E-01	0.34	1.41	0.55	0.62	5.34E-01
42	C*03:04-KIR2DL3-m2	227	111	-0.04	0.96	0.11	-0.40	6.91E-01	-0.08	0.92	0.11	-0.76	4.49E-01
43	C*04:01-KIR2DL3-m1	77	42	1.48	4.39	1.03	1.44	1.49E-01	1.57	4.83	1.03	1.53	1.25E-01
44	C*05:01-KIR2DL3-m1	144	82	0.44	1.56	0.46	0.96	3.39E-01	0.38	1.46	0.46	0.82	4.13E-01
45	C*06:02-KIR2DL3-m2	83	33	-0.06	0.94	0.18	-0.35	7.29E-01	-0.10	0.91	0.18	-0.54	5.90E-01
46	C*07:01-KIR2DL3-m2	286	148	0.10	1.11	0.10	0.99	3.20E-01	0.08	1.09	0.10	0.82	4.14E-01
47	C*07:02-KIR2DL3-m1	127	57	-0.27	0.76	0.42	-0.65	5.17E-01	-0.78	0.46	0.42	-1.85	6.41E-02
48	C*07:04-KIR2DL3-m2	22	11	0.02	1.02	0.31	0.06	9.53E-01	0.39	1.48	0.31	1.28	2.02E-01
49	C*08:02-KIR2DL3-m2	37	18	0.01	1.01	0.24	0.03	9.76E-01	-0.14	0.87	0.24	-0.59	5.54E-01
50	C*12:02-KIR2DL3-m2	9	1	-1.08	0.34	1.00	-1.08	2.82E-01	-0.51	0.60	1.00	-0.51	6.11E-01
51	C*12:03-KIR2DL3-m2	50	44	0.33	1.39	0.16	2.04	4.09E-02	0.21	1.24	0.16	1.32	1.88E-01

52	C*14:02-KIR2DL3-m1	13	8	0.08	1.09	1.08	0.08	9.39E-01	-0.67	0.51	1.08	-0.62	5.32E-01
53	C*15:02-KIR2DL3-m2	33	12	-0.46	0.63	0.29	-1.57	1.16E-01	-0.19	0.83	0.29	-0.64	5.25E-01
54	C*15:05-KIR2DL3-m2	9	3	-0.41	0.66	0.58	-0.71	4.75E-01	-0.84	0.43	0.58	-1.45	1.46E-01
55	C*16:01-KIR2DL3-m1	35	15	0.19	1.21	1.05	0.18	8.58E-01	0.66	1.94	1.05	0.63	5.26E-01
56	C*16:02-KIR2DL3-m2	4	5	0.48	1.61	0.45	1.06	2.91E-01	0.43	1.53	0.45	0.95	3.44E-01
57	R10-KIR2DL3-m2	29	18	0.29	1.34	0.24	1.21	2.27E-01	0.14	1.15	0.24	0.59	5.58E-01
58	C*01:02-KIR2DL4-m2	50	22	-0.20	0.82	0.22	-0.91	3.62E-01	0.03	1.03	0.22	0.11	9.09E-01
59	C*02:02-KIR2DL4-m2	62	18	-0.45	0.64	0.24	-1.85	6.43E-02	-0.35	0.70	0.24	-1.46	1.43E-01
60	C*03:03-KIR2DL4-m2	103	56	0.06	1.06	0.14	0.39	6.94E-01	-0.01	0.99	0.14	-0.07	9.45E-01
61	C*03:04-KIR2DL4-m2	241	122	-0.02	0.98	0.11	-0.17	8.63E-01	-0.06	0.95	0.11	-0.52	6.04E-01
62	C*04:01-KIR2DL4-m2	89	43	-0.16	0.86	0.16	-0.96	3.36E-01	-0.08	0.92	0.16	-0.53	5.99E-01
63	C*05:01-KIR2DL4-m2	158	88	0.07	1.07	0.12	0.58	5.63E-01	-0.02	0.98	0.12	-0.17	8.62E-01
64	C*06:02-KIR2DL4-m2	91	38	-0.05	0.95	0.17	-0.28	7.80E-01	-0.09	0.91	0.17	-0.54	5.88E-01
65	C*07:01-KIR2DL4-m2	309	163	0.13	1.14	0.10	1.31	1.90E-01	0.12	1.13	0.10	1.20	2.30E-01
66	C*07:02-KIR2DL4-m2	141	65	-0.11	0.90	0.14	-0.79	4.31E-01	-0.04	0.96	0.14	-0.32	7.52E-01
67	C*07:04-KIR2DL4-m2	25	11	-0.09	0.92	0.31	-0.29	7.72E-01	0.27	1.32	0.31	0.89	3.72E-01
68	C*08:02-KIR2DL4-m2	43	20	0.03	1.03	0.23	0.14	8.92E-01	-0.10	0.90	0.23	-0.44	6.59E-01
69	C*12:02-KIR2DL4-m2	9	2	-0.39	0.68	0.71	-0.54	5.86E-01	0.15	1.16	0.71	0.21	8.31E-01
70	C*12:03-KIR2DL4-m2	59	54	0.34	1.40	0.15	2.30	2.15E-02	0.20	1.22	0.15	1.35	1.76E-01
71	C*14:02-KIR2DL4-m2	17	9	0.14	1.15	0.34	0.41	6.83E-01	0.17	1.18	0.34	0.49	6.23E-01
72	C*15:02-KIR2DL4-m2	34	13	-0.46	0.63	0.28	-1.63	1.04E-01	-0.25	0.78	0.28	-0.88	3.79E-01
73	C*15:05-KIR2DL4-m2	9	4	-0.27	0.76	0.50	-0.53	5.94E-01	-0.69	0.50	0.50	-1.37	1.71E-01
74	C*16:01-KIR2DL4-m2	38	16	-0.05	0.95	0.26	-0.19	8.52E-01	0.00	1.00	0.26	0.02	9.87E-01
75	C*16:02-KIR2DL4-m2	5	6	0.61	1.83	0.41	1.47	1.41E-01	0.59	1.81	0.41	1.44	1.50E-01
76	R10-KIR2DL4-m2	38	20	0.13	1.14	0.23	0.57	5.72E-01	0.14	1.15	0.23	0.62	5.37E-01
77	C*01:02-KIR2DL5-m1	26	8	-0.40	0.67	0.45	-0.88	3.76E-01	-0.70	0.50	0.46	-1.54	1.24E-01
78	C*02:02-KIR2DL5-m2	29	10	-0.34	0.71	0.32	-1.07	2.84E-01	-0.18	0.83	0.32	-0.57	5.68E-01
79	C*03:03-KIR2DL5-m2	47	33	0.21	1.23	0.18	1.13	2.58E-01	0.01	1.01	0.18	0.07	9.41E-01
80	C*03:04-KIR2DL5-m1	116	56	-0.12	0.89	0.22	-0.54	5.88E-01	-0.15	0.86	0.22	-0.67	5.02E-01
81	C*04:01-KIR2DL5-m2	47	25	-0.09	0.92	0.21	-0.43	6.71E-01	-0.06	0.94	0.21	-0.28	7.78E-01
82	C*05:01-KIR2DL5-m1	74	40	-0.29	0.75	0.24	-1.18	2.38E-01	-0.17	0.84	0.24	-0.72	4.73E-01

83	C*06:02-KIR2DL5-m1	44	18	-0.11	0.90	0.34	-0.32	7.51E-01	0.05	1.05	0.34	0.15	8.85E-01
84	C*07:01-KIR2DL5-m2	147	88	0.26	1.29	0.12	2.13	3.36E-02	0.23	1.26	0.12	1.94	5.29E-02
85	C*07:02-KIR2DL5-m1	68	31	-0.14	0.87	0.27	-0.53	5.94E-01	-0.06	0.94	0.27	-0.22	8.24E-01
86	C*07:04-KIR2DL5-m1	15	5	-0.78	0.46	0.61	-1.27	2.06E-01	-0.38	0.69	0.61	-0.61	5.39E-01
87	C*08:02-KIR2DL5-m2	19	7	-0.06	0.94	0.38	-0.15	8.80E-01	-0.14	0.87	0.38	-0.36	7.20E-01
88	C*12:02-KIR2DL5-m2	6	2	-0.23	0.80	0.71	-0.32	7.50E-01	0.18	1.20	0.71	0.25	8.01E-01
89	C*12:03-KIR2DL5-m2	31	26	0.29	1.34	0.20	1.43	1.54E-01	0.14	1.15	0.20	0.70	4.86E-01
90	C*14:02-KIR2DL5-m2	9	7	0.32	1.37	0.38	0.83	4.08E-01	0.37	1.45	0.38	0.98	3.29E-01
91	C*15:02-KIR2DL5-m1	12	10	1.51	4.53	0.67	2.27	2.33E-02	1.17	3.23	0.67	1.76	7.80E-02
92	C*15:05-KIR2DL5-m2	6	3	-0.19	0.83	0.58	-0.33	7.41E-01	-0.64	0.53	0.58	-1.10	2.70E-01
93	C*16:01-KIR2DL5-m1	17	6	-0.46	0.63	0.53	-0.87	3.83E-01	-0.10	0.91	0.53	-0.19	8.52E-01
94	C*16:02-KIR2DL5-m1	3	3	-0.14	0.87	0.82	-0.17	8.67E-01	-0.67	0.51	0.82	-0.81	4.17E-01
95	R10-KIR2DL5-m1	23	10	-0.62	0.54	0.46	-1.36	1.74E-01	-0.40	0.67	0.46	-0.88	3.78E-01
96	C*01:02-KIR2DS1-m1	21	6	-0.65	0.52	0.49	-1.33	1.84E-01	-0.75	0.47	0.49	-1.52	1.28E-01
97	C*02:02-KIR2DS1-m2	20	7	-0.31	0.73	0.38	-0.81	4.16E-01	-0.23	0.79	0.38	-0.61	5.44E-01
98	C*03:03-KIR2DS1-m1	35	24	0.22	1.25	0.29	0.76	4.46E-01	-0.10	0.90	0.29	-0.36	7.20E-01
99	C*03:04-KIR2DS1-m1	91	46	-0.07	0.93	0.22	-0.31	7.55E-01	-0.22	0.81	0.22	-0.97	3.32E-01
100	C*04:01-KIR2DS1-m1	39	21	0.00	1.00	0.32	-0.01	9.90E-01	-0.19	0.83	0.32	-0.59	5.57E-01
101	C*05:01-KIR2DS1-m2	54	34	0.12	1.13	0.18	0.68	4.99E-01	0.13	1.14	0.18	0.72	4.72E-01
102	C*06:02-KIR2DS1-m1	36	14	-0.13	0.88	0.35	-0.37	7.13E-01	0.10	1.10	0.35	0.28	7.81E-01
103	C*07:01-KIR2DS1-m2	119	71	0.19	1.21	0.13	1.48	1.39E-01	0.21	1.23	0.13	1.57	1.17E-01
104	C*07:02-KIR2DS1-m2	51	24	-0.03	0.97	0.21	-0.16	8.75E-01	0.03	1.03	0.21	0.14	8.87E-01
105	C*07:04-KIR2DS1-m1	11	4	-0.57	0.57	0.64	-0.89	3.72E-01	-0.25	0.78	0.64	-0.39	6.94E-01
106	C*08:02-KIR2DS1-m1	12	7	0.37	1.44	0.48	0.76	4.45E-01	0.62	1.85	0.48	1.28	2.02E-01
107	C*12:02-KIR2DS1-m2	4	1	-0.29	0.75	1.00	-0.29	7.75E-01	0.15	1.16	1.00	0.15	8.79E-01
108	C*12:03-KIR2DS1-m2	20	21	0.38	1.46	0.22	1.70	8.94E-02	0.17	1.19	0.22	0.76	4.49E-01
109	C*14:02-KIR2DS1-m1	7	4	-0.24	0.79	0.68	-0.35	7.24E-01	-0.36	0.70	0.68	-0.53	5.98E-01
110	C*15:02-KIR2DS1-m1	10	8	1.24	3.44	0.58	2.13	3.31E-02	0.95	2.59	0.58	1.64	1.00E-01
111	C*15:05-KIR2DS1-m1	3	3	1.10	3.01	1.16	0.95	3.41E-01	0.97	2.63	1.16	0.83	4.05E-01
112	C*16:01-KIR2DS1-m1	14	3	-1.19	0.30	0.65	-1.83	6.73E-02	-0.63	0.53	0.65	-0.97	3.33E-01
113	C*16:02-KIR2DS1-m2	2	2	0.59	1.80	0.71	0.83	4.08E-01	0.78	2.18	0.71	1.10	2.73E-01

114	R10-KIR2DS1-m1	14	8	-0.11	0.90	0.47	-0.23	8.17E-01	-0.17	0.84	0.47	-0.36	7.19E-01
115	C*01:02-KIR2DS2-m1	25	6	-0.76	0.47	0.49	-1.56	1.20E-01	-0.52	0.59	0.49	-1.07	2.86E-01
116	C*02:02-KIR2DS2-m2	30	9	-0.42	0.65	0.34	-1.26	2.09E-01	-0.30	0.74	0.34	-0.89	3.76E-01
117	C*03:03-KIR2DS2-m1	54	27	-0.42	0.66	0.29	-1.46	1.44E-01	-0.24	0.79	0.29	-0.84	4.01E-01
118	C*03:04-KIR2DS2-m1	111	62	0.25	1.29	0.22	1.17	2.43E-01	0.34	1.40	0.22	1.55	1.20E-01
119	C*04:01-KIR2DS2-m1	44	21	-0.06	0.94	0.32	-0.20	8.41E-01	0.06	1.07	0.32	0.20	8.44E-01
120	C*05:01-KIR2DS2-m2	72	47	0.15	1.16	0.16	0.95	3.40E-01	-0.03	0.97	0.16	-0.16	8.71E-01
121	C*06:02-KIR2DS2-m2	46	18	-0.14	0.87	0.24	-0.58	5.64E-01	-0.24	0.79	0.24	-0.98	3.28E-01
122	C*07:01-KIR2DS2-m2	156	91	0.19	1.21	0.12	1.57	1.15E-01	0.12	1.13	0.12	0.99	3.20E-01
123	C*07:02-KIR2DS2-m2	68	33	-0.10	0.90	0.18	-0.56	5.75E-01	-0.03	0.97	0.18	-0.16	8.71E-01
124	C*07:04-KIR2DS2-m1	14	4	-0.60	0.55	0.64	-0.95	3.42E-01	-0.58	0.56	0.64	-0.91	3.64E-01
125	C*08:02-KIR2DS2-m2	22	6	-0.34	0.71	0.41	-0.84	4.04E-01	-0.30	0.74	0.41	-0.73	4.66E-01
126	C*12:02-KIR2DS2-m2	7	2	-0.19	0.82	0.71	-0.27	7.85E-01	0.48	1.61	0.71	0.67	5.05E-01
127	C*12:03-KIR2DS2-m2	34	30	0.45	1.57	0.19	2.39	1.67E-02	0.20	1.22	0.19	1.05	2.93E-01
128	C*14:02-KIR2DS2-m2	11	6	0.05	1.05	0.41	0.11	9.11E-01	0.21	1.23	0.41	0.50	6.17E-01
129	C*15:02-KIR2DS2-m2	10	4	-0.36	0.70	0.50	-0.71	4.79E-01	-0.37	0.69	0.50	-0.74	4.58E-01
130	C*15:05-KIR2DS2-m2	7	3	-0.40	0.67	0.58	-0.68	4.95E-01	-0.83	0.44	0.58	-1.43	1.53E-01
131	C*16:01-KIR2DS2-m1	16	9	0.82	2.28	0.51	1.60	1.10E-01	0.71	2.03	0.51	1.38	1.69E-01
132	C*16:02-KIR2DS2-m1	4	3	-0.47	0.63	0.82	-0.57	5.70E-01	-0.82	0.44	0.82	-0.99	3.22E-01
133	R10-KIR2DS2-m1	30	10	-0.81	0.44	0.46	-1.77	7.72E-02	-1.32	0.27	0.46	-2.87	4.11E-03
134	C*01:02-KIR2DS3-m1	13	4	-0.39	0.68	0.56	-0.69	4.89E-01	-0.46	0.63	0.57	-0.82	4.11E-01
135	C*02:02-KIR2DS3-m2	19	5	-0.53	0.59	0.45	-1.19	2.35E-01	-0.34	0.71	0.45	-0.75	4.54E-01
136	C*03:03-KIR2DS3-m1	29	21	0.06	1.06	0.30	0.20	8.39E-01	-0.14	0.87	0.30	-0.48	6.33E-01
137	C*03:04-KIR2DS3-m1	57	36	0.37	1.45	0.24	1.54	1.23E-01	0.37	1.45	0.24	1.55	1.22E-01
138	C*04:01-KIR2DS3-m2	26	11	-0.32	0.72	0.31	-1.06	2.89E-01	-0.13	0.88	0.31	-0.42	6.75E-01
139	C*05:01-KIR2DS3-m1	34	19	-0.37	0.69	0.29	-1.29	1.96E-01	-0.35	0.70	0.29	-1.23	2.19E-01
140	C*06:02-KIR2DS3-m2	23	7	-0.30	0.74	0.38	-0.79	4.27E-01	-0.18	0.84	0.38	-0.47	6.42E-01
141	C*07:01-KIR2DS3-m2	69	46	0.28	1.33	0.16	1.81	7.05E-02	0.17	1.19	0.16	1.10	2.73E-01
142	C*07:02-KIR2DS3-m2	39	17	-0.24	0.79	0.25	-0.96	3.39E-01	0.01	1.01	0.25	0.05	9.64E-01
143	C*07:04-KIR2DS3-m1	8	3	-0.60	0.55	0.69	-0.88	3.78E-01	-0.23	0.80	0.69	-0.33	7.40E-01
144	C*08:02-KIR2DS3-m1	14	3	-0.83	0.44	0.64	-1.30	1.94E-01	-0.76	0.47	0.64	-1.20	2.30E-01

145	C*12:02-KIR2DS3-m2	3	2	0.13	1.13	0.71	0.18	8.59E-01	0.55	1.74	0.71	0.77	4.39E-01
146	C*12:03-KIR2DS3-m1	20	16	0.09	1.09	0.32	0.26	7.91E-01	-0.16	0.86	0.32	-0.49	6.28E-01
147	C*14:02-KIR2DS3-m2	5	6	0.64	1.90	0.41	1.56	1.19E-01	0.63	1.89	0.41	1.54	1.23E-01
148	C*15:02-KIR2DS3-m1	5	5	1.05	2.86	0.58	1.81	7.03E-02	0.82	2.27	0.58	1.41	1.59E-01
149	C*15:05-KIR2DS3-m2	3	1	-0.63	0.53	1.00	-0.63	5.30E-01	-0.97	0.38	1.00	-0.97	3.31E-01
150	C*16:01-KIR2DS3-m2	7	4	0.19	1.21	0.50	0.38	7.04E-01	0.39	1.47	0.50	0.77	4.44E-01
151	C*16:02-KIR2DS3-m1	2	1	-0.31	0.74	1.10	-0.28	7.80E-01	-1.30	0.27	1.10	-1.18	2.38E-01
152	R10-KIR2DS3-m1	16	4	-0.95	0.39	0.57	-1.67	9.57E-02	-0.42	0.66	0.57	-0.74	4.62E-01
153	C*01:02-KIR2DS4-m1	46	21	-0.30	0.74	1.05	-0.28	7.79E-01	-1.35	0.26	1.05	-1.28	2.01E-01
154	C*02:02-KIR2DS4-m2	61	18	-0.44	0.65	0.24	-1.81	6.98E-02	-0.35	0.71	0.24	-1.44	1.49E-01
155	C*03:03-KIR2DS4-m1	100	52	-0.99	0.37	0.58	-1.72	8.56E-02	-0.65	0.52	0.58	-1.12	2.62E-01
156	C*03:04-KIR2DS4-m2	230	115	-0.04	0.97	0.11	-0.32	7.47E-01	-0.07	0.93	0.11	-0.65	5.16E-01
157	C*04:01-KIR2DS4-m2	82	39	-0.19	0.82	0.17	-1.15	2.51E-01	-0.10	0.91	0.17	-0.58	5.65E-01
158	C*05:01-KIR2DS4-m1	149	86	1.24	3.44	0.76	1.64	1.02E-01	1.14	3.11	0.76	1.50	1.33E-01
159	C*06:02-KIR2DS4-m2	87	35	-0.08	0.93	0.18	-0.42	6.71E-01	-0.13	0.87	0.18	-0.75	4.53E-01
160	C*07:01-KIR2DS4-m2	292	155	0.13	1.13	0.10	1.22	2.22E-01	0.10	1.11	0.10	1.00	3.19E-01
161	C*07:02-KIR2DS4-m2	134	62	-0.12	0.89	0.14	-0.83	4.04E-01	-0.06	0.94	0.14	-0.45	6.56E-01
162	C*07:04-KIR2DS4-m2	24	11	-0.05	0.95	0.31	-0.16	8.72E-01	0.31	1.37	0.31	1.01	3.10E-01
163	C*08:02-KIR2DS4-m2	43	20	0.03	1.03	0.23	0.14	8.92E-01	-0.10	0.90	0.23	-0.44	6.59E-01
164	C*12:02-KIR2DS4-m2	9	2	-0.39	0.68	0.71	-0.54	5.86E-01	0.15	1.16	0.71	0.21	8.31E-01
165	C*12:03-KIR2DS4-m2	58	52	0.32	1.38	0.15	2.15	3.12E-02	0.20	1.22	0.15	1.35	1.79E-01
166	C*14:02-KIR2DS4-m2	17	9	0.14	1.15	0.34	0.41	6.83E-01	0.17	1.18	0.34	0.49	6.23E-01
167	C*15:02-KIR2DS4-m2	32	12	-0.49	0.61	0.29	-1.66	9.71E-02	-0.30	0.74	0.29	-1.02	3.09E-01
168	C*15:05-KIR2DS4-m2	9	3	-0.41	0.66	0.58	-0.71	4.75E-01	-0.84	0.43	0.58	-1.45	1.46E-01
169	C*16:01-KIR2DS4-m2	38	15	-0.07	0.93	0.26	-0.26	7.92E-01	-0.04	0.96	0.26	-0.15	8.81E-01
170	C*16:02-KIR2DS4-m2	4	6	0.63	1.88	0.41	1.54	1.24E-01	0.63	1.88	0.41	1.53	1.26E-01
171	R10-KIR2DS4-m2	35	18	0.09	1.10	0.24	0.39	6.94E-01	0.12	1.13	0.24	0.49	6.23E-01
172	C*01:02-KIR2DS5-m1	17	4	-0.52	0.60	0.56	-0.92	3.59E-01	-0.68	0.51	0.56	-1.21	2.26E-01
173	C*02:02-KIR2DS5-m1	15	6	0.31	1.37	0.51	0.61	5.40E-01	0.35	1.41	0.51	0.68	4.98E-01
174	C*03:03-KIR2DS5-m2	28	16	0.23	1.26	0.26	0.91	3.61E-01	0.09	1.10	0.26	0.37	7.13E-01
175	C*03:04-KIR2DS5-m1	75	36	-0.14	0.87	0.23	-0.60	5.47E-01	-0.23	0.79	0.23	-1.00	3.18E-01

176	C*04:01-KIR2DS5-m1	33	17	-0.03	0.97	0.33	-0.09	9.28E-01	-0.22	0.80	0.33	-0.68	4.99E-01
177	C*05:01-KIR2DS5-m2	46	23	0.04	1.04	0.21	0.17	8.68E-01	0.06	1.06	0.22	0.28	7.82E-01
178	C*06:02-KIR2DS5-m2	30	14	0.05	1.05	0.27	0.18	8.61E-01	0.06	1.07	0.27	0.23	8.15E-01
179	C*07:01-KIR2DS5-m2	104	62	0.27	1.31	0.14	1.93	5.41E-02	0.30	1.35	0.14	2.15	3.18E-02
180	C*07:02-KIR2DS5-m2	43	20	0.03	1.03	0.23	0.12	9.04E-01	0.07	1.07	0.23	0.30	7.65E-01
181	C*07:04-KIR2DS5-m1	10	3	-0.73	0.48	0.69	-1.06	2.89E-01	-0.60	0.55	0.69	-0.88	3.81E-01
182	C*08:02-KIR2DS5-m1	8	5	0.13	1.14	0.53	0.24	8.09E-01	0.36	1.44	0.53	0.69	4.93E-01
183	C*12:02-KIR2DS5-m2	4	1	-0.29	0.75	1.00	-0.29	7.75E-01	0.15	1.16	1.00	0.15	8.79E-01
184	C*12:03-KIR2DS5-m1	16	16	-0.15	0.86	0.32	-0.46	6.43E-01	-0.23	0.79	0.32	-0.73	4.65E-01
185	C*14:02-KIR2DS5-m2	5	2	-0.12	0.88	0.71	-0.17	8.62E-01	0.17	1.19	0.71	0.24	8.11E-01
186	C*15:02-KIR2DS5-m1	9	7	0.97	2.63	0.57	1.71	8.75E-02	0.62	1.85	0.57	1.08	2.78E-01
187	C*15:05-KIR2DS5-m1	3	3	1.10	3.00	1.16	0.95	3.44E-01	0.92	2.52	1.16	0.80	4.26E-01
188	C*16:01-KIR2DS5-m1	12	3	-0.85	0.43	0.65	-1.31	1.91E-01	-0.48	0.62	0.65	-0.74	4.62E-01
189	C*16:02-KIR2DS5-m2	2	2	0.59	1.80	0.71	0.83	4.08E-01	0.78	2.18	0.71	1.10	2.73E-01
190	R10-KIR2DS5-m2	13	8	0.16	1.18	0.36	0.46	6.47E-01	0.14	1.14	0.36	0.38	7.06E-01
191	C*01:02-KIR3DL1-m1	47	21	-0.57	0.57	1.05	-0.54	5.88E-01	-1.53	0.22	1.05	-1.45	1.47E-01
192	C*02:02-KIR3DL1-m2	61	18	-0.44	0.65	0.24	-1.81	6.98E-02	-0.35	0.71	0.24	-1.44	1.49E-01
193	C*03:03-KIR3DL1-m1	100	52	-0.98	0.37	0.58	-1.70	8.96E-02	-0.65	0.52	0.58	-1.12	2.64E-01
194	C*03:04-KIR3DL1-m2	230	115	-0.04	0.97	0.11	-0.32	7.47E-01	-0.07	0.93	0.11	-0.65	5.16E-01
195	C*04:01-KIR3DL1-m2	83	39	-0.20	0.82	0.17	-1.17	2.40E-01	-0.10	0.91	0.17	-0.58	5.61E-01
196	C*05:01-KIR3DL1-m1	149	86	1.25	3.50	0.76	1.66	9.76E-02	1.14	3.12	0.76	1.51	1.32E-01
197	C*06:02-KIR3DL1-m2	87	35	-0.08	0.93	0.18	-0.42	6.71E-01	-0.13	0.87	0.18	-0.75	4.53E-01
198	C*07:01-KIR3DL1-m2	292	155	0.13	1.13	0.10	1.22	2.22E-01	0.10	1.11	0.10	1.00	3.19E-01
199	C*07:02-KIR3DL1-m2	134	62	-0.12	0.89	0.14	-0.83	4.04E-01	-0.06	0.94	0.14	-0.45	6.56E-01
200	C*07:04-KIR3DL1-m2	24	11	-0.05	0.95	0.31	-0.16	8.72E-01	0.31	1.37	0.31	1.01	3.10E-01
201	C*08:02-KIR3DL1-m2	43	20	0.03	1.03	0.23	0.14	8.92E-01	-0.10	0.90	0.23	-0.44	6.59E-01
202	C*12:02-KIR3DL1-m2	9	2	-0.39	0.68	0.71	-0.54	5.86E-01	0.15	1.16	0.71	0.21	8.31E-01
203	C*12:03-KIR3DL1-m2	58	52	0.32	1.38	0.15	2.15	3.12E-02	0.20	1.22	0.15	1.35	1.79E-01
204	C*14:02-KIR3DL1-m2	17	9	0.14	1.15	0.34	0.41	6.83E-01	0.17	1.18	0.34	0.49	6.23E-01
205	C*15:02-KIR3DL1-m2	32	12	-0.49	0.61	0.29	-1.66	9.71E-02	-0.30	0.74	0.29	-1.02	3.09E-01
206	C*15:05-KIR3DL1-m2	9	3	-0.41	0.66	0.58	-0.71	4.75E-01	-0.84	0.43	0.58	-1.45	1.46E-01

207	C*16:01-KIR3DL1-m2	38	15	-0.07	0.93	0.26	-0.26	7.92E-01	-0.04	0.96	0.26	-0.15	8.81E-01
208	C*16:02-KIR3DL1-m2	4	6	0.63	1.88	0.41	1.54	1.24E-01	0.63	1.88	0.41	1.53	1.26E-01
209	R10-KIR3DL1-m2	35	18	0.09	1.10	0.24	0.39	6.94E-01	0.12	1.13	0.24	0.49	6.23E-01
210	C*01:02-KIR3DL2-m2	50	22	-0.20	0.82	0.22	-0.91	3.62E-01	0.03	1.03	0.22	0.11	9.09E-01
211	C*02:02-KIR3DL2-m2	62	18	-0.45	0.64	0.24	-1.85	6.43E-02	-0.35	0.70	0.24	-1.46	1.43E-01
212	C*03:03-KIR3DL2-m2	103	56	0.06	1.06	0.14	0.39	6.94E-01	-0.01	0.99	0.14	-0.07	9.45E-01
213	C*03:04-KIR3DL2-m2	241	122	-0.02	0.98	0.11	-0.17	8.63E-01	-0.06	0.95	0.11	-0.52	6.04E-01
214	C*04:01-KIR3DL2-m2	89	43	-0.16	0.86	0.16	-0.96	3.36E-01	-0.08	0.92	0.16	-0.53	5.99E-01
215	C*05:01-KIR3DL2-m2	158	88	0.07	1.07	0.12	0.58	5.63E-01	-0.02	0.98	0.12	-0.17	8.62E-01
216	C*06:02-KIR3DL2-m2	91	38	-0.05	0.95	0.17	-0.28	7.80E-01	-0.09	0.91	0.17	-0.54	5.88E-01
217	C*07:01-KIR3DL2-m2	309	163	0.13	1.14	0.10	1.31	1.90E-01	0.12	1.13	0.10	1.20	2.30E-01
218	C*07:02-KIR3DL2-m2	141	65	-0.11	0.90	0.14	-0.79	4.31E-01	-0.04	0.96	0.14	-0.32	7.52E-01
219	C*07:04-KIR3DL2-m2	25	11	-0.09	0.92	0.31	-0.29	7.72E-01	0.27	1.32	0.31	0.89	3.72E-01
220	C*08:02-KIR3DL2-m2	43	20	0.03	1.03	0.23	0.14	8.92E-01	-0.10	0.90	0.23	-0.44	6.59E-01
221	C*12:02-KIR3DL2-m2	9	2	-0.39	0.68	0.71	-0.54	5.86E-01	0.15	1.16	0.71	0.21	8.31E-01
222	C*12:03-KIR3DL2-m2	59	54	0.34	1.40	0.15	2.30	2.15E-02	0.20	1.22	0.15	1.35	1.76E-01
223	C*14:02-KIR3DL2-m2	17	9	0.14	1.15	0.34	0.41	6.83E-01	0.17	1.18	0.34	0.49	6.23E-01
224	C*15:02-KIR3DL2-m2	34	13	-0.46	0.63	0.28	-1.63	1.04E-01	-0.25	0.78	0.28	-0.88	3.79E-01
225	C*15:05-KIR3DL2-m2	9	4	-0.27	0.76	0.50	-0.53	5.94E-01	-0.69	0.50	0.50	-1.37	1.71E-01
226	C*16:01-KIR3DL2-m2	38	16	-0.05	0.95	0.26	-0.19	8.52E-01	0.00	1.00	0.26	0.02	9.87E-01
227	C*16:02-KIR3DL2-m2	5	6	0.61	1.83	0.41	1.47	1.41E-01	0.59	1.81	0.41	1.44	1.50E-01
228	R10-KIR3DL2-m2	38	20	0.13	1.14	0.23	0.57	5.72E-01	0.14	1.15	0.23	0.62	5.37E-01
229	C*01:02-KIR3DS1-m1	23	7	-0.49	0.61	0.47	-1.04	2.98E-01	-0.81	0.44	0.47	-1.72	8.56E-02
230	C*02:02-KIR3DS1-m2	20	8	-0.22	0.80	0.36	-0.62	5.36E-01	-0.25	0.78	0.36	-0.70	4.86E-01
231	C*03:03-KIR3DS1-m1	35	23	0.23	1.26	0.29	0.79	4.30E-01	-0.09	0.91	0.29	-0.32	7.48E-01
232	C*03:04-KIR3DS1-m1	88	45	0.01	1.01	0.22	0.03	9.78E-01	-0.18	0.84	0.22	-0.80	4.24E-01
233	C*04:01-KIR3DS1-m2	39	20	-0.11	0.90	0.23	-0.48	6.31E-01	-0.07	0.93	0.23	-0.29	7.69E-01
234	C*05:01-KIR3DS1-m2	56	30	0.00	1.00	0.19	-0.03	9.79E-01	0.04	1.05	0.19	0.23	8.15E-01
235	C*06:02-KIR3DS1-m1	32	11	-0.18	0.83	0.37	-0.49	6.21E-01	0.39	1.47	0.38	1.03	3.05E-01
236	C*07:01-KIR3DS1-m2	120	66	0.11	1.12	0.13	0.84	3.98E-01	0.15	1.16	0.13	1.12	2.65E-01
237	C*07:02-KIR3DS1-m2	51	24	-0.03	0.97	0.21	-0.16	8.75E-01	0.03	1.03	0.21	0.14	8.87E-01

238	C*07:04-KIR3DS1-m1	11	4	-0.51	0.60	0.64	-0.80	4.22E-01	-0.23	0.79	0.64	-0.37	7.13E-01
239	C*08:02-KIR3DS1-m1	12	6	0.21	1.24	0.50	0.43	6.70E-01	0.43	1.53	0.50	0.85	3.96E-01
240	C*12:02-KIR3DS1-m1	4	1	0.13	1.14	1.42	0.09	9.26E-01	-0.33	0.72	1.42	-0.23	8.17E-01
241	C*12:03-KIR3DS1-m2	18	19	0.42	1.52	0.24	1.77	7.64E-02	0.26	1.29	0.24	1.10	2.73E-01
242	C*14:02-KIR3DS1-m1	6	4	0.17	1.19	0.68	0.26	7.98E-01	-0.28	0.75	0.68	-0.42	6.77E-01
243	C*15:02-KIR3DS1-m1	10	8	1.29	3.65	0.58	2.23	2.57E-02	0.97	2.64	0.58	1.67	9.51E-02
244	C*15:05-KIR3DS1-m1	2	3	1.53	4.64	1.16	1.32	1.86E-01	1.26	3.52	1.16	1.09	2.78E-01
245	C*16:01-KIR3DS1-m1	14	2	-1.50	0.22	0.76	-1.97	4.91E-02	-1.09	0.34	0.76	-1.43	1.53E-01
246	C*16:02-KIR3DS1-m2	1	2	0.69	1.99	0.71	0.97	3.33E-01	0.80	2.22	0.71	1.12	2.62E-01
247	R10-KIR3DS1-m2	12	6	0.03	1.04	0.41	0.08	9.33E-01	0.44	1.55	0.41	1.06	2.91E-01
248	C*01:02-KIR2DP1-m2	50	21	-0.23	0.79	0.22	-1.05	2.95E-01	0.00	1.00	0.22	-0.01	9.90E-01
249	C*02:02-KIR2DP1-m2	62	18	-0.45	0.64	0.24	-1.85	6.43E-02	-0.35	0.70	0.24	-1.46	1.43E-01
250	C*03:03-KIR2DP1-m2	101	55	0.06	1.06	0.15	0.42	6.73E-01	-0.01	0.99	0.15	-0.08	9.34E-01
251	C*03:04-KIR2DP1-m2	239	122	-0.01	0.99	0.11	-0.10	9.24E-01	-0.05	0.95	0.11	-0.45	6.51E-01
252	C*04:01-KIR2DP1-m2	87	43	-0.14	0.87	0.16	-0.89	3.74E-01	-0.07	0.93	0.16	-0.42	6.73E-01
253	C*05:01-KIR2DP1-m1	154	86	0.09	1.09	0.80	0.11	9.14E-01	-0.36	0.70	0.80	-0.45	6.54E-01
254	C*06:02-KIR2DP1-m1	89	36	-0.27	0.76	0.81	-0.34	7.35E-01	0.42	1.52	0.81	0.52	6.06E-01
255	C*07:01-KIR2DP1-m2	301	157	0.13	1.14	0.10	1.27	2.02E-01	0.11	1.12	0.10	1.12	2.64E-01
256	C*07:02-KIR2DP1-m1	138	64	-0.02	0.98	1.06	-0.02	9.82E-01	-1.43	0.24	1.06	-1.34	1.79E-01
257	C*07:04-KIR2DP1-m2	24	11	-0.07	0.93	0.31	-0.24	8.12E-01	0.30	1.35	0.31	0.98	3.28E-01
258	C*08:02-KIR2DP1-m2	39	20	0.06	1.07	0.23	0.27	7.84E-01	-0.07	0.93	0.23	-0.31	7.56E-01
259	C*12:02-KIR2DP1-m2	9	2	-0.39	0.68	0.71	-0.54	5.86E-01	0.15	1.16	0.71	0.21	8.31E-01
260	C*12:03-KIR2DP1-m2	59	50	0.30	1.34	0.15	1.95	5.07E-02	0.20	1.22	0.15	1.31	1.91E-01
261	C*14:02-KIR2DP1-m2	15	9	0.18	1.20	0.34	0.55	5.85E-01	0.18	1.20	0.34	0.54	5.88E-01
262	C*15:02-KIR2DP1-m2	34	13	-0.46	0.63	0.28	-1.63	1.04E-01	-0.25	0.78	0.28	-0.88	3.79E-01
263	C*15:05-KIR2DP1-m2	9	4	-0.27	0.76	0.50	-0.53	5.94E-01	-0.69	0.50	0.50	-1.37	1.71E-01
264	C*16:01-KIR2DP1-m1	36	15	0.24	1.27	1.09	0.22	8.23E-01	0.57	1.76	1.09	0.52	6.03E-01
265	C*16:02-KIR2DP1-m2	4	6	0.64	1.89	0.41	1.55	1.21E-01	0.60	1.82	0.41	1.45	1.47E-01
266	R10-KIR2DP1-m2	36	20	0.16	1.17	0.23	0.69	4.92E-01	0.15	1.16	0.23	0.65	5.17E-01
267	C*01:02-KIR3DP1-m2	50	22	-0.20	0.82	0.22	-0.91	3.62E-01	0.03	1.03	0.22	0.11	9.09E-01
268	C*02:02-KIR3DP1-m2	62	18	-0.45	0.64	0.24	-1.85	6.43E-02	-0.35	0.70	0.24	-1.46	1.43E-01

269	C*03:03-KIR3DP1-m2	103	56	0.06	1.06	0.14	0.39	6.94E-01	-0.01	0.99	0.14	-0.07	9.45E-01
270	C*03:04-KIR3DP1-m2	241	122	-0.02	0.98	0.11	-0.17	8.63E-01	-0.06	0.95	0.11	-0.52	6.04E-01
271	C*04:01-KIR3DP1-m2	89	43	-0.16	0.86	0.16	-0.96	3.36E-01	-0.08	0.92	0.16	-0.53	5.99E-01
272	C*05:01-KIR3DP1-m2	158	88	0.07	1.07	0.12	0.58	5.63E-01	-0.02	0.98	0.12	-0.17	8.62E-01
273	C*06:02-KIR3DP1-m2	91	38	-0.05	0.95	0.17	-0.28	7.80E-01	-0.09	0.91	0.17	-0.54	5.88E-01
274	C*07:01-KIR3DP1-m2	309	163	0.13	1.14	0.10	1.31	1.90E-01	0.12	1.13	0.10	1.20	2.30E-01
275	C*07:02-KIR3DP1-m2	141	65	-0.11	0.90	0.14	-0.79	4.31E-01	-0.04	0.96	0.14	-0.32	7.52E-01
276	C*07:04-KIR3DP1-m2	25	11	-0.09	0.92	0.31	-0.29	7.72E-01	0.27	1.32	0.31	0.89	3.72E-01
277	C*08:02-KIR3DP1-m2	43	20	0.03	1.03	0.23	0.14	8.92E-01	-0.10	0.90	0.23	-0.44	6.59E-01
278	C*12:02-KIR3DP1-m2	9	2	-0.39	0.68	0.71	-0.54	5.86E-01	0.15	1.16	0.71	0.21	8.31E-01
279	C*12:03-KIR3DP1-m2	59	54	0.34	1.40	0.15	2.30	2.15E-02	0.20	1.22	0.15	1.35	1.76E-01
280	C*14:02-KIR3DP1-m2	17	9	0.14	1.15	0.34	0.41	6.83E-01	0.17	1.18	0.34	0.49	6.23E-01
281	C*15:02-KIR3DP1-m2	34	13	-0.46	0.63	0.28	-1.63	1.04E-01	-0.25	0.78	0.28	-0.88	3.79E-01
282	C*15:05-KIR3DP1-m2	9	4	-0.27	0.76	0.50	-0.53	5.94E-01	-0.69	0.50	0.50	-1.37	1.71E-01
283	C*16:01-KIR3DP1-m2	38	16	-0.05	0.95	0.26	-0.19	8.52E-01	0.00	1.00	0.26	0.02	9.87E-01
284	C*16:02-KIR3DP1-m2	5	6	0.61	1.83	0.41	1.47	1.41E-01	0.59	1.81	0.41	1.44	1.50E-01
285	R10-KIR3DP1-m2	38	20	0.13	1.14	0.23	0.57	5.72E-01	0.14	1.15	0.23	0.62	5.37E-01

ESM Figure 1. Crystal structure of the interacting HLA-A*11:01 loaded with a viral peptide, with the KIR2DS2 activating receptor [6]. This is the approximate orientation that all three known HLA-I–KIR structures assume. The NK cell bearing the KIR receptor molecules, and the nucleated target-cell bearing HLA-I molecules have their cell membrane planes nearly perpendicular to the plane of the screen/paper. For details, full molecular coordinates and access to a simple software that allows rotation of the complex, see 4N8V.pdb in www.rcsb.org.

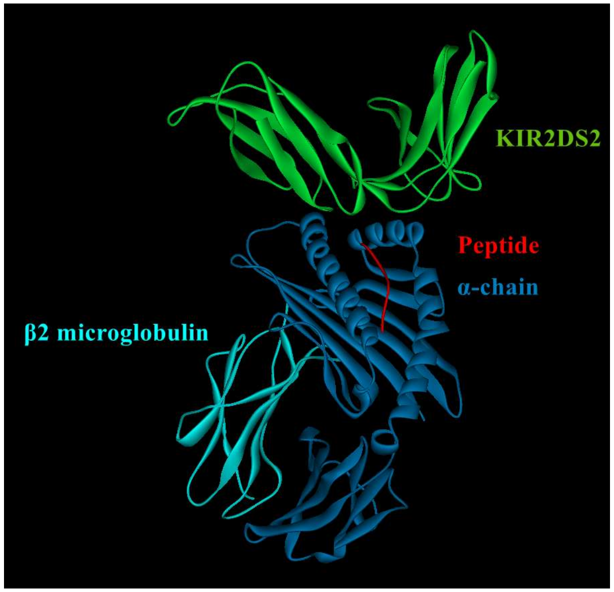

ESM Figure **2**. Up to four different HLA-I molecules from heterozygous genotype with and without a KIR receptor.

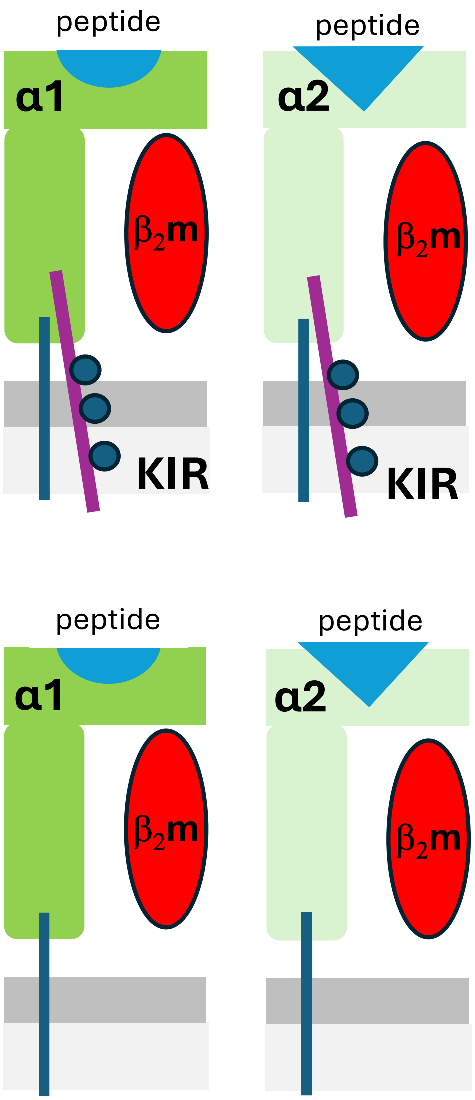

ESM Figure 3. Visual display ligand-receptor interactions of KIR genes with A) HLA-A, B) HLA-B and C) HLA-C

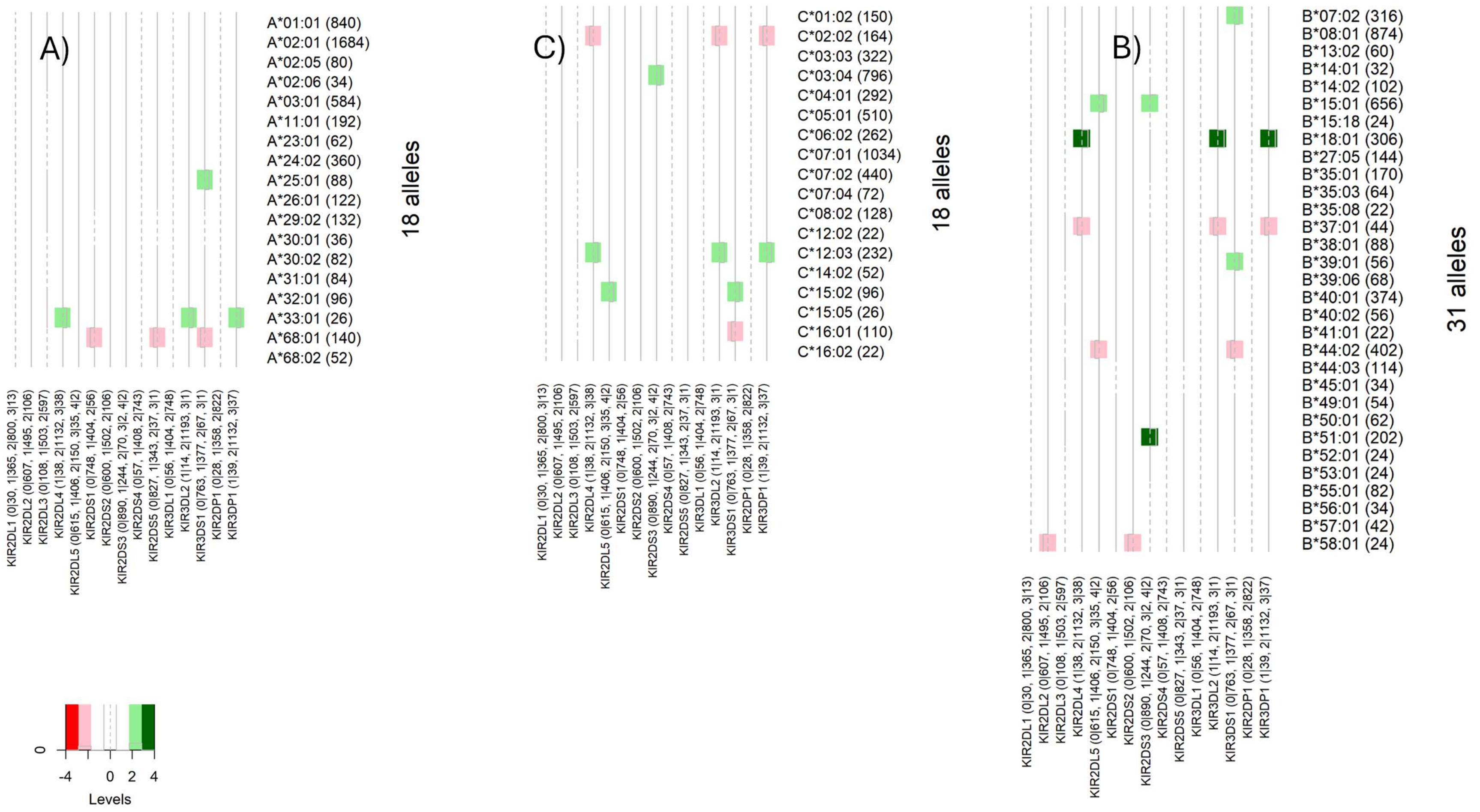

[1] Hsu JC (1996) Multiple comparisons: theory and methods. Chapman & Hall, London
[2] Yang JHM, Khatri L, Mickunas M, et al. (2019) Phenotypic Analysis of Human Lymph Nodes in Subjects With New-Onset Type 1 Diabetes and Healthy Individuals by Flow Cytometry. Front Immunol 10: 2547. 10.3389/fimmu.2019.02547
[3] Boughter CT, Meier-Schellersheim M (2023) Conserved biophysical compatibility among the highly variable germline-encoded regions shapes TCR-MHC interactions. Elife 12. 10.7554/eLife.90681
[4] Waterhouse A, Bertoni M, Bienert S, et al. (2018) SWISS-MODEL: homology modelling of protein structures and complexes. Nucleic Acids Res 46(W1): W296-W303. 10.1093/nar/gky427
[5] Pende D, Falco M, Vitale M, et al. (2019) Killer Ig-Like Receptors (KIRs): Their Role in NK Cell Modulation and Developments Leading to Their Clinical Exploitation. Front Immunol 10: 1179. 10.3389/fimmu.2019.01179
[6] Liu J, Xiao Z, Ko HL, Shen M, Ren EC (2014) Activating killer cell immunoglobulin-like receptor 2DS2 binds to HLA-A*11. Proc Natl Acad Sci U S A 111(7): 2662-2667. 10.1073/pnas.1322052111
[7] Yang Y, Bai H, Wu Y, et al. (2022) Activating receptor KIR2DS2 bound to HLA-C1 reveals the novel recognition features of activating receptor. Immunology 165(3): 341-354. 10.1111/imm.13439
[8] Pymm P, Illing PT, Ramarathinam SH, et al. (2017) MHC-I peptides get out of the groove and enable a novel mechanism of HIV-1 escape. Nat Struct Mol Biol 24(4): 387-394. 10.1038/nsmb.3381
[9] Sukhwal A, Sowdhamini R (2013) Oligomerisation status and evolutionary conservation of interfaces of protein structural domain superfamilies. Mol Biosyst 9(7): 1652-1661. 10.1039/c3mb25484d
